# Drug-induced Sweet’s syndrome: pharmacovigilance insights from FAERS with a cross-database consistency assessment in VigiBase via LASSO and multivariable logistic regression

**DOI:** 10.3389/fimmu.2025.1622736

**Published:** 2025-09-23

**Authors:** Yuhan Xie, Qinxiao Li, Jing Zhou, Xiangqi Qin, Zhe Zhang, Ruimin Bai

**Affiliations:** ^1^ Department of Dermatology, the First Affiliated Hospital of Xi’an Jiaotong University, Xi’an, Shaanxi, China; ^2^ Department of Dermatology, the Second Affiliated Hospital of Xi’an Jiaotong University, Xi’an, Shaanxi, China; ^3^ Xi’an Jiaotong University, Xi’an, Shaanxi, China

**Keywords:** drug-induced Sweet’s syndrome, FAERS, VigiBase, pharmacovigilance, disproportionality analysis, LASSO regression, multivariable logistic regression

## Abstract

**Background:**

Drug-induced Sweet’s syndrome (DISS), a rare but serious adverse drug reaction characterized by acute febrile neutrophilic dermatosis, remains difficult to identify due to its low incidence and diverse drug triggers.

**Methods:**

Drugs associated with DISS were systematically identified and characterized using data from the U.S. Food and Drug Administration Adverse Event Reporting System (FAERS; Q1 2004–Q4 2024). Reports were analyzed for baseline characteristics, comorbidities, time-to-onset, drug class distributions, and polypharmacy patterns assessed through drug co-occurrence network analysis. Disproportionality analysis identified candidate drugs, which were refined using the least absolute shrinkage and selection operator (LASSO) regression and multivariable logistic regression. The main analysis excluded malignancy- and/or immune-related indications, with two sensitivity analyses to assess robustness. A cross-database consistency assessment was conducted in VigiBase, supplemented by PubMed literature review and product label examination.

**Results:**

A total of 2,018 DISS cases involving 342 drugs were identified. The median time to onset was 22 (interquartile range: 7–98) days, with 55.60% occurring within 30 days. Ninety drugs demonstrated positive disproportionality signals; a similar pattern was observed in the subset of reports submitted by medical doctors. Of these, 24 remained significant in the main model (area under the curve = 0.815, 95% confidence interval: 0.775–0.856), predominantly comprising antineoplastic and anti-infective agents. Sensitivity analyses produced comparable results. Cross-database assessment in VigiBase identified overlap for 10 signals, while literature review supported associations for 15 drugs and 9 were documented as associated with SS in the product labels.

**Conclusion:**

This study provides a comprehensive evaluation of drugs associated with DISS using real-world pharmacovigilance data. The results reveal both established and previously unrecognized drug triggers, offering important insights to support early detection, clinical management, and improved drug safety monitoring from statistical and clinical perspectives.

## 1 Introduction

Sweet’s syndrome (SS), also known as acute febrile neutrophilic dermatosis, was first described by Dr. Robert Douglas Sweet in 1964 (1). It is a rare inflammatory dermatosis characterized clinically by the abrupt onset of fever and painful erythematous plaques or nodules, typically affecting the upper limbs, trunk, and head or neck, and histopathologically by a dense neutrophilic infiltrate in the upper dermis (2). The pathogenesis of SS is considered multifactorial, involving dysregulated cytokine signaling, immune dysregulation, genetic susceptibility, and various external triggers, including infections, malignancies (particularly hematologic), autoimmune disorders, and pharmacologic agents (3). Based on etiologic factors, SS is classified into three clinical subtypes: classical (idiopathic), malignancy-associated, and drug-induced Sweet’s syndrome (DISS). Among these, DISS represents an especially important yet underrecognized subtype that presents unique diagnostic challenges and distinct therapeutic implications. A definitive diagnosis of DISS requires fulfillment of all of the following criteria: (A) abrupt onset of painful erythematous skin lesions; (B) histopathologic evidence of dense neutrophilic infiltration without leukocytoclastic vasculitis; (C) fever >38°C; (D) a clear temporal relationship between drug exposure and symptom onset, or recurrence upon rechallenge; and (E) resolution of lesions following drug withdrawal or treatment with systemic corticosteroids (4). To date, an increasing number of drugs have been implicated in the development of DISS, with granulocyte colony-stimulating factors (G-CSFs), azathioprine, all-trans retinoic acid (ATRA), and Fms-like tyrosine kinase 3 (FLT3) inhibitors among the most frequently reported (3, 5). Notably, clinical improvement and symptom resolution can often be achieved by discontinuation of the suspected agent alone. However, the current understanding of drug-related risk in DISS remains limited, as most data are derived from individual case reports or small case series (3, 5–7). The absence of large-scale, systematic investigations limits accurate assessment of the incidence and causality of DISS across different drug classes. Therefore, real-world pharmacovigilance data are urgently needed to expand our knowledge of drug-induced triggers and to improve early recognition and clinical management.

The U.S. Food and Drug Administration Adverse Event Reporting System (FAERS) is one of the largest publicly available pharmacovigilance databases, designed to collect reports on adverse drug events (ADEs), medication errors, and product quality issues related to both pharmaceutical drugs and biologic therapies. FAERS plays a critical role in post-marketing surveillance, supporting signal detection, risk evaluation, and regulatory decision-making (8). It enables identification of potential drug safety concerns that may not be evident during pre-approval clinical trials.

To our knowledge, this is the first FAERS-based study to systematically evaluate drugs associated with DISS while accounting for polypharmacy and key confounding factors. Disproportionality analysis, least absolute shrinkage and selection operator (LASSO) regression, and multivariable logistic regression were applied, supplemented by sensitivity analyses, external assessment using VigiBase, targeted literature review, and product label examination, to generate robust real-world evidence. These findings provide valuable evidence to support early identification and prevention of DISS and offer critical insights to inform clinical risk stratification and pharmacovigilance strategies.

## 2 Methods

### 2.1 Data source

Data were extracted from FAERS via publicly available ASCII files provided on the OpenFDA platform (https://open.fda.gov/data/faers/). FAERS consists of seven relational tables: Demographics (DEMO), Drug Information (DRUG), Adverse Reactions (REAC), Outcomes (OUTC), Report Sources (RPSR), Therapy Dates (THER), and Indications (INDI).

All quarterly FAERS datasets from the first quarter of 2004 (Q1 2004) to the fourth quarter of 2024 (Q4 2024) were included. Duplicate reports were removed in accordance with U.S. Food and Drug Administration (FDA) deduplication guidelines: for entries with the same CASEID, the record with the most recent FDA_DT (date of FDA receipt) was retained; if both CASEID and FDA_DT were identical, the record with the highest PRIMARYID was selected to ensure data integrity.

As FAERS is a publicly available, de-identified, and anonymized dataset, the use of these data does not require ethical approval or institutional review board oversight.

### 2.2 Definition of adverse events and suspect drugs

Cases of DISS were identified using the Preferred Term (PT) “Sweet syndrome” (MedDRA code: 10042458) from the REAC table. To minimize confounding from concomitant or non-implicated medications, only drugs labeled as the Primary Suspect (role code: PS) were included.

Drug names were standardized using the World Health Organization (WHO) Drug Dictionary to ensure consistency across reports. Standardized names were further mapped to the Anatomical Therapeutic Chemical (ATC) Classification System at both Level 1 (anatomical main group) and Level 2 (therapeutic subgroup).

### 2.3 Statistical analyses

#### 2.3.1 Time-to-onset analysis

For reports with complete information on both therapy start and adverse event onset dates, time-to-onset was calculated as the number of days from the initiation of the suspected drug to the occurrence of SS. Results were summarized using medians and interquartile ranges (IQRs).

#### 2.3.2 Signal detection via disproportionality analysis

Disproportionality analysis was conducted using the reporting odds ratio (ROR) and its 95% confidence interval (CI) to identify potential pharmacovigilance signals (8). ROR compares the frequency of a specific adverse event for a given drug with that for all other drugs using a 2×2 contingency table (**Supplementary Table S1**). The ROR was calculated as: $ROR=\frac{a/c}{b/d}$ . The 95% CI for the ROR was computed as: $\;ROR_{95\%CI}=e^{ln(ROR)\;\pm \;1.96\sqrt{(\frac{1}{a}+\frac{1}{b}+\frac{1}{c}+\frac{1}{d})}}$ . A signal was considered positive if the number of reported cases exceeded 3 and the lower limit of the 95% CI of the ROR was greater than 1. *P*-values were calculated using Fisher’s exact test, and multiple comparisons were adjusted using the false discovery rate (FDR) method (9).

#### 2.3.3 Identification of risk factors

To address potential indication-related confounding from malignancy- or immune-associated SS subtypes, three analytical datasets were predefined: The main analysis excluded all reports with malignancy-related and/or immune-related indications; Sensitivity analysis A excluded only malignancy-related indications; Sensitivity analysis B excluded only immune-related indications. For each dataset, disproportionality analysis was conducted independently to identify candidate drug signals, applying the same predefined criteria (≥3 reports and a lower 95% CI of the ROR > 1). Demographic variables (age and sex) and drug variables identified as positive signals in the disproportionality analysis were considered candidate predictors. The LASSO was employed for variable selection and to prevent overfitting. Ten-fold cross-validation was used to determine the optimal regularization parameter (λ), with two criteria evaluated: λ_min [yielding the highest cross-validated area under the curve (AUC)] and λ_1se (the most parsimonious model within one standard error of the minimum AUC). Variables retained by the LASSO model were subsequently included in a multivariable logistic regression analysis, with overall model discrimination assessed by AUC.

#### 2.3.4 Cross-database and external evidence assessment of drug signals

Disproportionality analysis was replicated in VigiBase, the WHO’s global pharmacovigilance database. A signal was considered positive if the drug had at least three reported cases and the lower bound of the 95% CI of the ROR exceeded 1. In parallel, a targeted literature review was conducted in PubMed by combining each drug name with terms such as “*Sweet’s syndrome*” or “*acute febrile neutrophilic dermatosis*”. Finally, official product labels were examined to assess whether SS was listed as an adverse reaction. Label information was sourced from the FDA database and European Medicines Agency (EMA). A drug was considered labeled if either condition, or a synonymous term, appeared in the adverse reactions section of the prescribing information.

All data processing and statistical analyses were performed using PostgreSQL (version 14.4) and R software (version 4.4.2). LASSO regression was conducted using the “glmnet” package, and multivariable logistic regression was implemented with the base R function “glm()” (family = binomial). A two-sided adjusted *P* value < 0.05 was considered statistically significant.

## 3 Results

### 3.1 Descriptive overview of DISS reports in FAERS

From Q1 2004 to Q4 2024, a total of 21,964,449 adverse event reports were recorded in the FAERS database, of which 18,278,243 unique cases were remained after deduplication. Among these, 2,018 cases were identified as associated with DISS. The cohort included 772 (38.26%) males and 1,038 (51.44%) females, with sex information missing in 208 cases (10.31%). After excluding 22.60% of cases with missing age data, the median age was 55 years (IQR: 41–68). Following the exclusion of 84.54% of cases lacking body weight data, the median body weight was 70 kg (IQR: 60.95–85.29). The majority of reports were submitted by medical doctors (41.23%), followed by health professionals (19.47%) and consumers (9.71%). The majority of cases originated from the United States (35.68%), followed by France (12.04%) and Canada (7.09%) (**Table 1**).

**Table 1:** Demographic and clinical characteristics of drug-induced Sweet’s syndrome reports in the FAERS database.

Characteristic	Number	Proportion (%)
Number of Cases	2018	100%
Sex		
Male	772	38.26%
Female	1038	51.44%
Missing	208	10.31%
Age (year)		
Mean (SD)	53.37 (18.31)	
Median [Q1, Q3]	55 [41, 68]	
Missing	456	22.60%
Weight (kg)		
Mean (SD)	72.85 (20.05)	
Median [Q1, Q3]	70 [60.95, 85.29]	
Missing	1706	84.54%
Reported Person		
Medical Doctor	832	41.23%
Health Professional	393	19.47%
Consumer	196	9.71%
Pharmacist	77	3.82%
Lawyer	2	0.10%
Registered Nurse	1	0.05%
Others	425	21.06%
Missing	92	4.56%
Reported Country (Top Six)		
United States	720	35.68%
France	243	12.04%
Canada	143	7.09%
Spain	102	5.05%
Japan	94	4.66%
United Kingdom	92	4.56%
Others	564	27.95%
Missing	60	2.97%
Indications		
Observed	1890	93.66%
Missing	128	6.34%
Outcome		
Observed	1989	98.56%
Missing	29	1.44%

FAERS, U.S. Food and Drug Administration adverse event reporting system.

**Figure 1:**
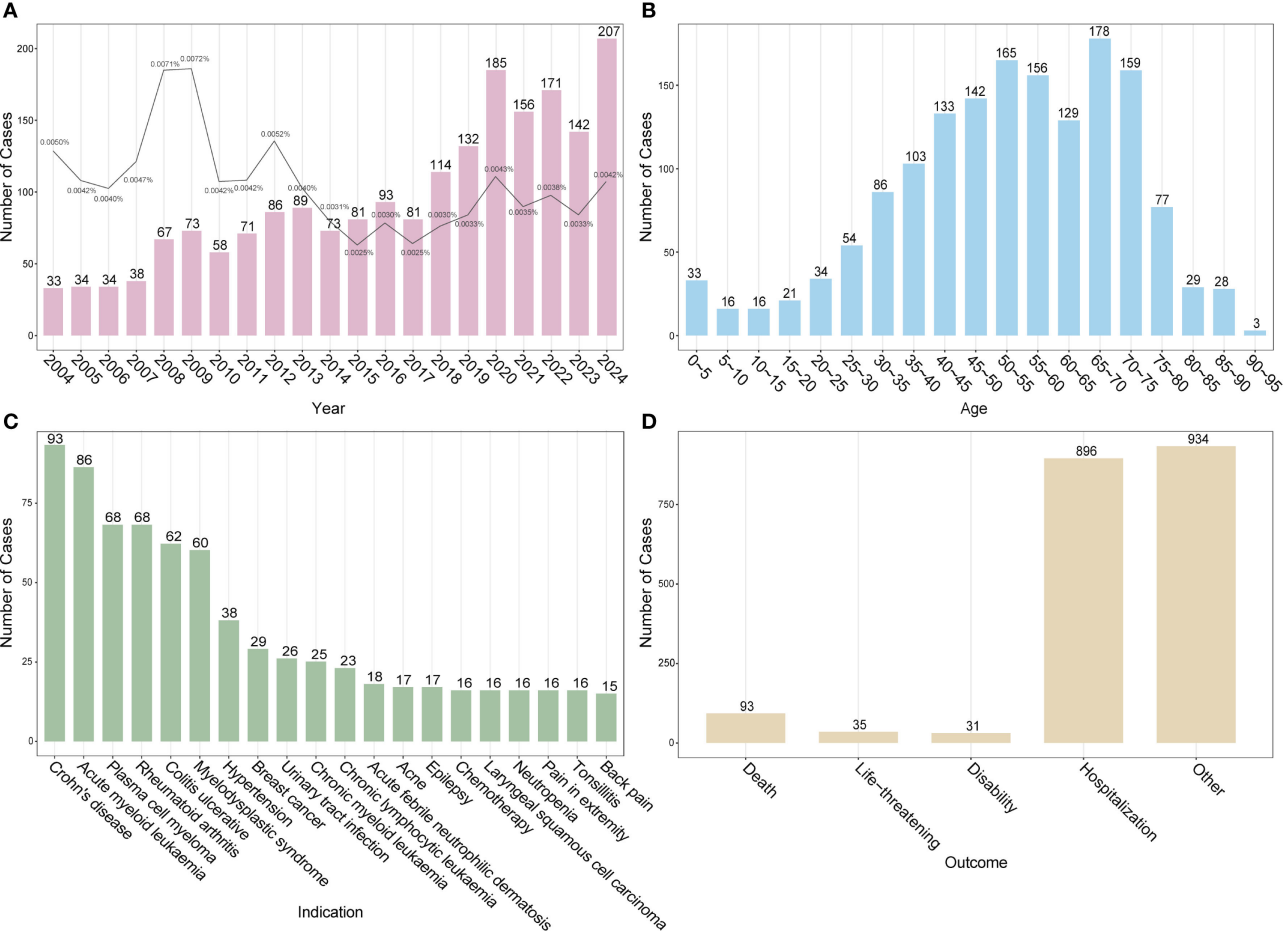
Baseline characteristics of drug-induced Sweet’s syndrome reports in the FAERS database. **(A)** Annual number of reported DISS cases (bars) with the proportion among all adverse event reports in FAERS (line). **(B)** Age distribution of affected individuals. **(C)** Top 20 underlying indications associated with DISS. **(D)** Clinical outcomes of reported adverse events. DISS, drug-induced Sweet’s syndrome; FAERS, FDA adverse event reporting system.

Temporal trends revealed a steady rise in DISS reports over the past two decades, with the number of cases in 2024 more than six times that in 2004. The proportion of DISS cases among all FAERS reports remained extremely low and fluctuated over time without a clear increasing or decreasing trend (**Figure 1A**). Age-stratified analysis indicated that reports were most frequent among individuals aged 40–75 years (**Figure 1B**). Among the underlying indications, neoplastic diseases accounted for the largest proportion (37.0%), followed by autoimmune/rheumatic conditions (13.2%) and infectious diseases (10.7%) (**Supplementary Figure S1**). The most commonly reported underlying indications included Crohn’s disease (CD), acute myeloid leukemia (AML), plasma cell myeloma, rheumatoid arthritis (RA), ulcerative colitis (UC), and myelodysplastic syndrome (MDS) (**Figure 1C**). In terms of clinical outcomes, hospitalization was the most frequently reported consequence, whereas death, life-threatening conditions, and disability were relatively uncommon (**Figure 1D**). Among the top 10 drugs reported in the six countries with the highest case counts, adalimumab was implicated in five countries, excluding Japan. Other commonly reported drugs included infliximab and azacitidine. Notably, in Canada, infliximab and methotrexate accounted for 17.48% and 16.08% of DISS reports, respectively. In Spain, gabapentin was the most frequently implicated agent (14.71%), whereas in Japan, azacitidine was the leading drug (12.77%) (**Supplementary Table S2**).

### 3.2 Time-to-onset distribution of DISS

**Figure 2:**
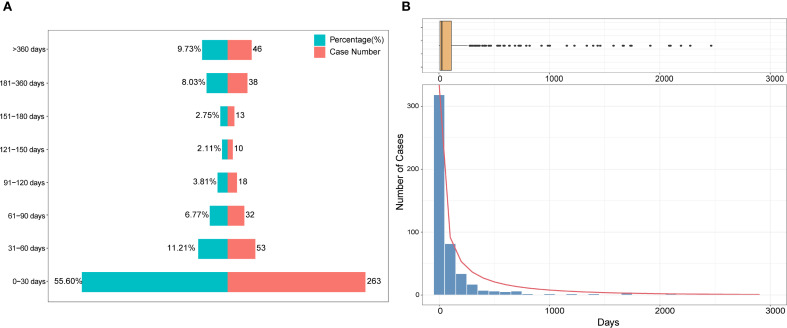
Time-to-onset distribution of drug-induced Sweet’s syndrome. **(A)** Stacked bar chart showing the proportion (%) and number of cases across defined latency intervals. **(B)** Histogram with an overlaid density curve illustrating the overall distribution of latency durations, accompanied by a boxplot above to indicate the spread and potential outliers. A Weibull model was fitted to the data to characterize the temporal pattern.

Among the 2018 reported cases of DISS, time-to-onset data were available for 473 cases (23.4%). The median time to onset was 22 days (IQR: 7–98 days). Of these, 55.60% occurred within the first 30 days following drug exposure, 11.21% between 31 and 60 days, and 6.77% between 61 and 90 days (**Figure 2A**). Notably, the frequency of reported cases declined substantially with increasing time to onset. A Weibull model was fitted to the time-to-onset data, revealing a right-skewed distribution consistent with acute onset in most cases (**Figure 2B**). Additionally, a small number of outliers exhibited markedly delayed onset, with intervals exceeding 1,000 days.

### 3.3 Descriptive analysis of drug spectrum and therapeutic classifications

From Q1 2004 to Q4 2024, a total of 342 distinct drugs were reported in association with SS in the FAERS database. The most frequently implicated agents included azathioprine (n = 84), sulfamethoxazole/trimethoprim (n = 79), adalimumab (n = 76), infliximab (n = 76), azacitidine (n = 69), hydralazine (n = 50), lenalidomide (n = 41), filgrastim (n = 36), pegfilgrastim (n = 35), and methotrexate (n = 32) (**Supplementary Table S3**). A drug co-occurrence network was constructed to illustrate commonly co-reported medications in DISS cases, reflecting real-world polypharmacy patterns (**Supplementary Figure S2**). Frequent combinations included azathioprine or infliximab with corticosteroids (e.g., prednisone, methylprednisolone), celecoxib with cyclobenzaprine, ciprofloxacin with nitrofurantoin, and the chemotherapeutic pair carboplatin with docetaxel. Other commonly co-reported agents included acetylsalicylic acid, amoxicillin/clavulanic acid, allopurinol, and cisplatin.

Reports submitted by medical doctors were analyzed separately, identifying 215 distinct drugs. The most frequently reported agents included sulfamethoxazole/trimethoprim (n = 52), infliximab (n = 34), adalimumab (n = 33), azacitidine (n = 27), bortezomib (n = 18), lenalidomide (n = 18), pegfilgrastim (n = 18), azathioprine (n = 16), hydralazine (n = 16), and levofloxacin (n = 16). Among these, 7 of the top 10 drugs overlapped with those in the overall dataset. Furthermore, 29 of the top 30 and 39 of the top 50 most frequently reported drugs were shared between the two datasets (**Supplementary Table S4**).

Based on the ATC classification system, the 342 identified drugs were categorized into 14 pharmacological groups: antineoplastic and immunomodulating agents (n = 135), anti-infectives for systemic use (n = 44), alimentary tract and metabolism drugs (n = 30), cardiovascular system drugs (n = 27), nervous system drugs (n = 26), dermatologicals (n = 23), musculoskeletal system drugs (n = 13), blood and blood-forming organ drugs (n = 11), genito-urinary system and sex hormones (n = 10), systemic hormonal preparations excluding sex hormones and insulins (n = 7), various (n = 7), respiratory system drugs (n = 6), antiparasitic products, insecticides, and repellents (n = 2), and sensory organ drugs (n = 1) (**Supplementary Table S5**). In terms of case distribution, antineoplastic and immunomodulating agents accounted for the highest proportion of DISS reports (1,083/2,018, 53.67%), with 549 reports attributed to antineoplastic agents and 405 to immunosuppressants. This was followed by anti-infectives for systemic use (257/2,018, 12.73%), the majority of which were antibacterials (221/257). Among cardiovascular system drugs (152/2,018, 7.53%), the most frequently reported subclasses included antihypertensives (53/152), cardiac therapy agents (31/152), and diuretics (27/152). For alimentary tract and metabolism drugs (152/2,018, 7.53%), the leading categories were stomatological preparations (68/152) and antidiarrheals, intestinal anti-inflammatory/anti-infective agents (56/152). In contrast, drugs targeting the respiratory system and sensory organs were least frequently reported (**Supplementary Table S5**).

**Figure 3:**
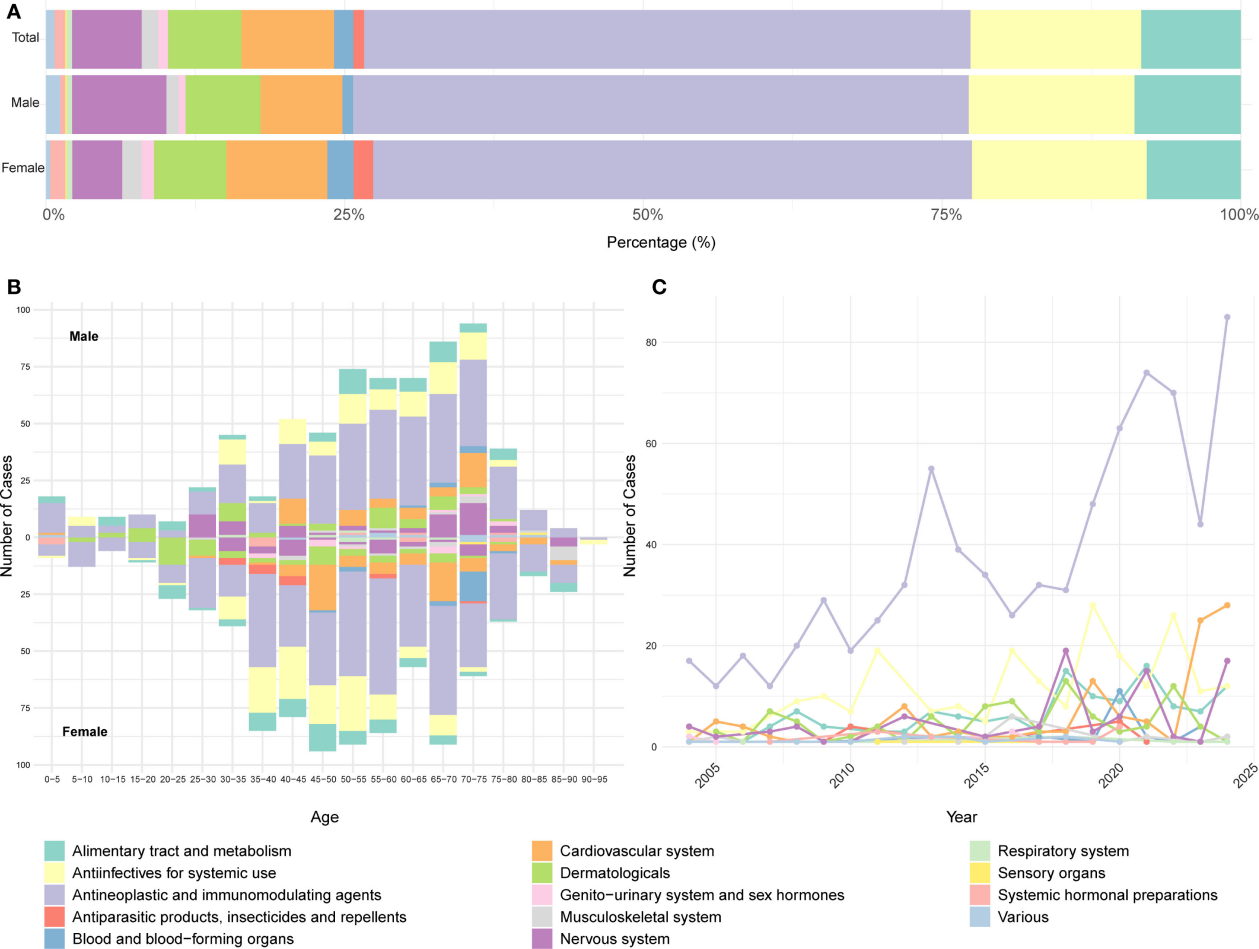
Distribution of drug classes in DISS reported in the FAERS database based on ATC classification. **(A)** Proportional distribution of reported drug classes. **(B)** Stratification of reported cases by age and sex. **(C)** Annual trend in the number of reports by drug class. A consistent color scheme is used across all panels. FAERS, U.S. Food and Drug Administration Adverse Event Reporting System; ATC, Anatomical Therapeutic Chemical.

After excluding cases with missing sex or age information, a total of 1,547 DISS reports were included in the stratified analysis. Antineoplastic and immunomodulating agents remained the most frequently implicated drug class in both males and females, followed by anti-infectives for systemic use, alimentary tract and metabolism drugs, and cardiovascular system drugs (**Figure 3A**). Chi-square analysis revealed a significant association between sex and drug class distribution (χ² = 34.97, df = 13, *P* < 0.001), with certain classes disproportionately reported by one sex over the other (**Supplementary Table S6**). Age- and sex-specific analysis revealed that DISS was most commonly reported in males aged 50–75 years and females aged 35–70 years. The association between drug class and age group was statistically significant (χ² = 714.1, df = 234, *P* < 2.2×10^-16^) (**Supplementary Table S7**). Antineoplastic and immunomodulating agents were consistently implicated across all age groups in both sexes. By contrast, anti-infectives for systemic use and nervous system drugs were mainly reported in individuals aged 25–75 years, while cardiovascular system drugs were more frequently reported in cases aged 40–75 years. Notably, antiparasitic products, insecticides, and repellents were disproportionately reported among younger and middle-aged females, with very few cases observed in males. Similarly, blood and blood-forming organ drugs were primarily reported in older females, with limited representation among males (**Figure 3B**). Linear regression analysis was performed to assess temporal trends over the 2004–2024 period. Antineoplastic and immunomodulating agents exhibited the most pronounced upward trend (slope = 2.87, R² = 0.704, *P* < 0.001), followed by drugs targeting the alimentary tract and metabolism (slope = 0.50, *P* < 0.001) and cardiovascular system (slope = 0.69, *P* = 0.007) (**Supplementary Table S8**). In contrast, the reporting frequency of other drug classes remained relatively stable and low throughout the study period (**Figure 3C**).

### 3.4 Disproportionality analysis

**Figure 4:**
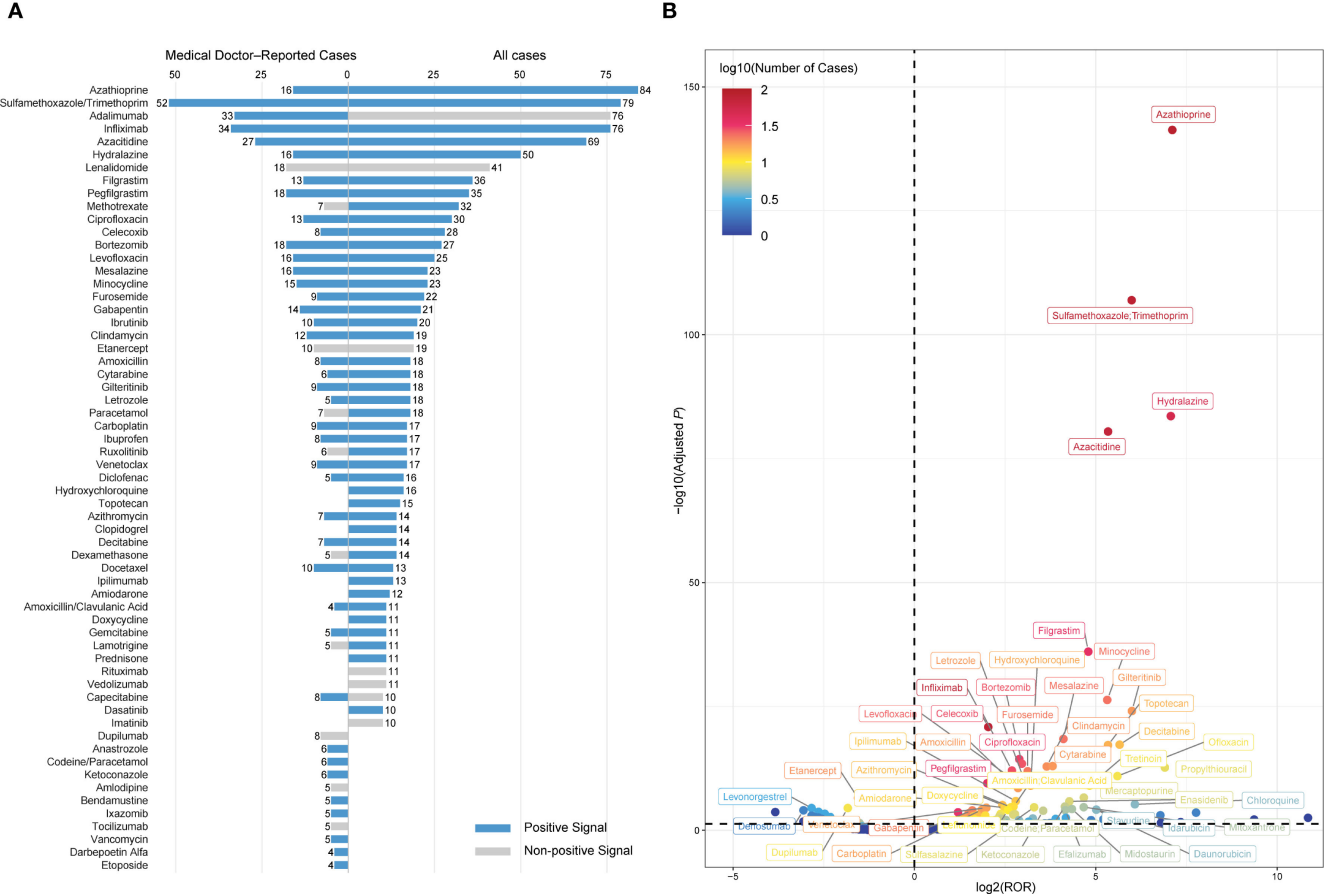
Drug signals associated with DISS identified through disproportionality analysis. **(A)** Top 50 drugs ranked by the number of reported cases. Each bar represents the number of reports submitted by medical doctors (left) or all reporters (right). Blue bars indicate drugs with a positive signal, defined as ≥3 cases and a lower bound of the 95% confidence interval for the ROR > 1, while grey bars represent drugs that did not meet the criteria. **(B)** Volcano plot visualizing the distribution of drug signals. The x-axis represents the log_2_-transformed ROR, and the y-axis displays the –log_10_-transformed adjusted *P* values. The color gradient reflects the log_10_-transformed number of cases. DISS, drug-induced Sweet’s syndrome; ROR, reporting odds ratio; adjusted *P*, false discovery rate-adjusted *P* value.

Disproportionality analysis was conducted to detect potential drug-related safety signals. A drug was identified as a positive signal if reported cases ≥3 and a lower bound of the 95% CI for ROR >1. Based on this criterion, 90 drugs were identified as significantly associated with SS (**Supplementary Table S3**), and blue shading in the table denotes drugs with a positive signal. Interestingly, several drugs with a relatively high number of reports—such as adalimumab (n = 76), lenalidomide (n = 41), etanercept (n = 19), rituximab (n = 11), and vedolizumab (n = 11)—did not meet the threshold for signal detection due to insufficient disproportionality (the lower bound of the 95% CI of the ROR ≤ 1) (**Figure 4A**). A volcano plot was generated based on disproportionality analysis. Each point represents a drug, with the x-axis indicating the base-2 logarithm of the ROR (log_2_ ROR) and the y-axis showing the negative logarithm of the FDR-adjusted *P* value. Among the drugs most strongly associated were azathioprine, sulfamethoxazole/trimethoprim, hydralazine, and azacitidine (**Figure 4B**).

A subset disproportionality analysis restricted to medical doctor–submitted reports identified 57 positive signals among 215 drugs. The most frequently implicated agents were sulfamethoxazole/trimethoprim (n = 52), infliximab (n = 34), adalimumab (n = 33), azacitidine (n = 27), bortezomib (n = 18), lenalidomide (n = 18), pegfilgrastim (n = 18), azathioprine (n = 16), hydralazine (n = 16), and levofloxacin (n = 16) (**Figure 4A**). Notably, several frequently reported drugs did not meet the disproportionality threshold for a positive signal, including lenalidomide (n = 18), etanercept (n = 10), dupilumab (n = 8), methotrexate (n = 7), acetaminophen (n = 7), and ruxolitinib (n = 6). A complementary external assessment was performed using disproportionality analysis in VigiBase, a targeted literature review, and examination of regulatory product labels. Lenalidomide demonstrated a positive signal in VigiBase. Lenalidomide, ruxolitinib, paracetamol, tocilizumab, lamotrigine, and dupilumab were supported by published case reports. However, none of the drugs were listed as associated with SS in product labels approved by the FDA or EMA (**Supplementary Table S9**).

### 3.5 Identification of risk-associated drugs via LASSO and multivariable regression

**Figure 5:**
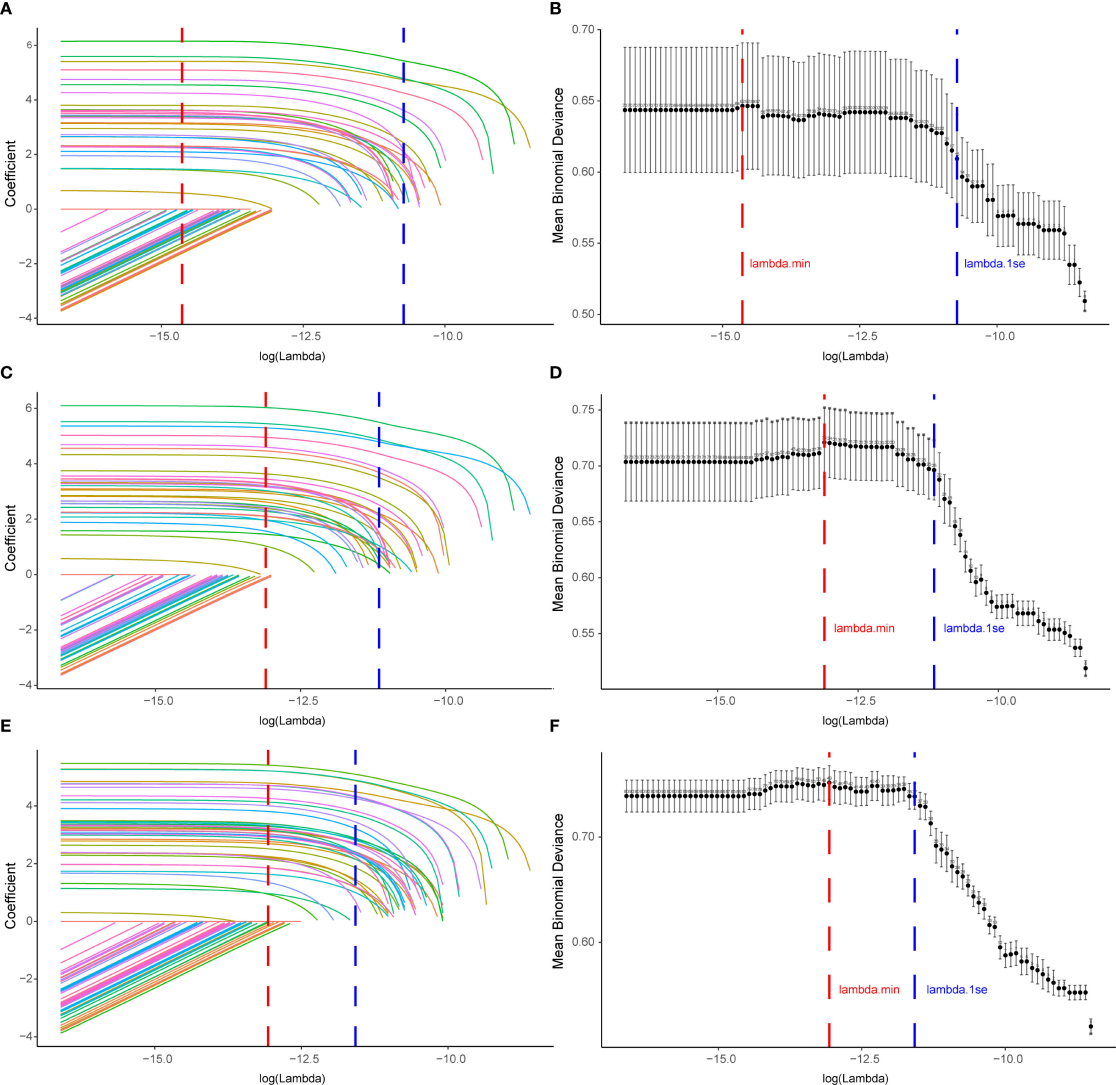
Variable selection for drug−induced Sweet’s syndrome using LASSO across the main and sensitivity analyses. **(A, C, E)** LASSO coefficient paths for all candidate variables; coefficients shrink toward zero as log(λ) increases. Vertical dashed lines denote λ_min (red; minimum cross−validated error) and λ_1se (blue; most regularized model within one SE of the minimum). **(B, D, F)** Ten−fold cross−validation deviance curves; points show mean binomial deviance with error bars for ±1 SE, and the numbers above the curve indicate the count of non−zero coefficients. Panels **(A, B)** correspond to the main analysis (excluding malignancy− and autoimmune−related indications), **(C, D)** to Sensitivity Analysis A (excluding malignancy only), and **(E, F)** to Sensitivity Analysis B (excluding autoimmune only). LASSO, least absolute shrinkage and selection operator; SE, standard error.

**Figure 6:**
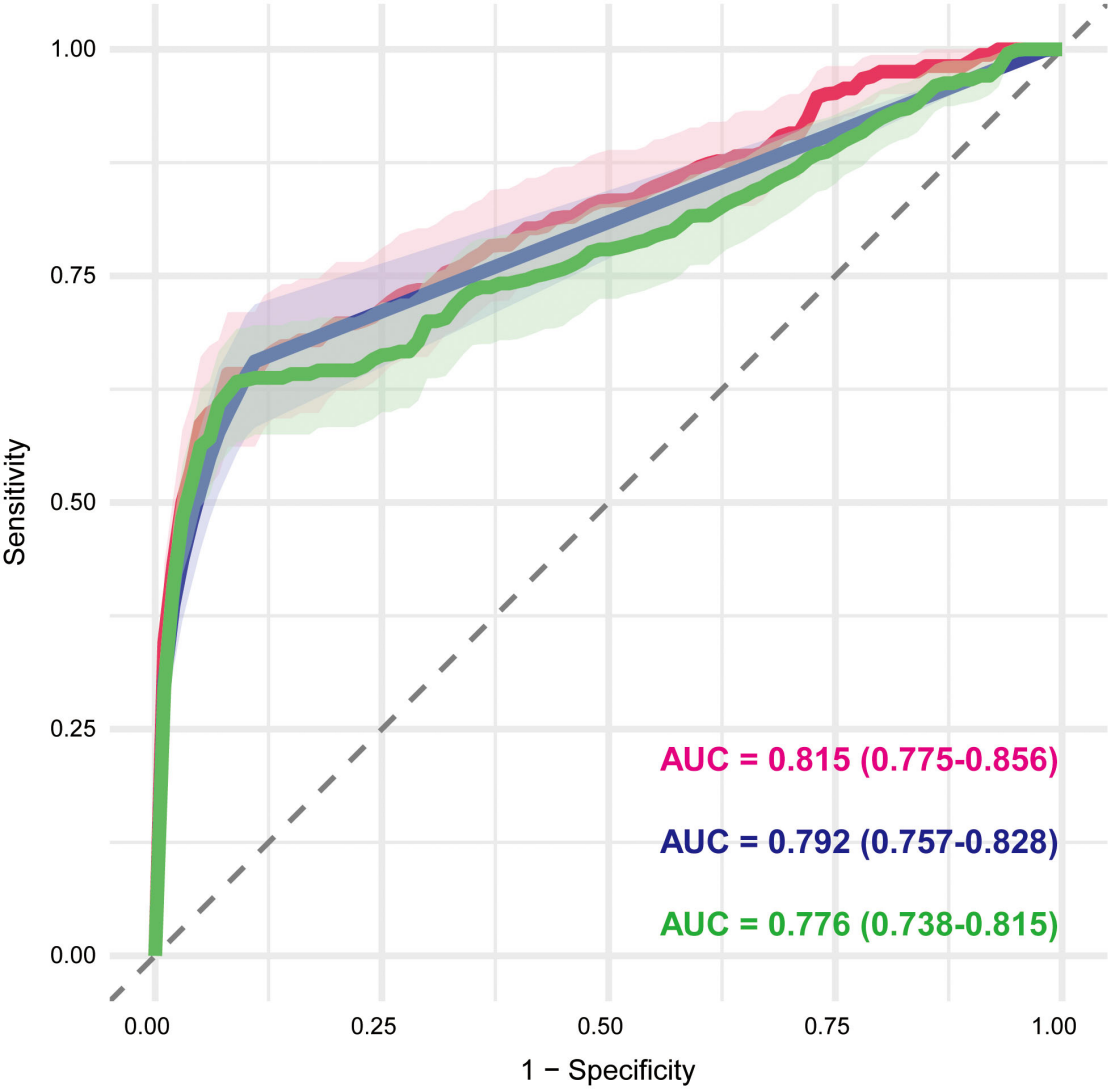
Receiver operating characteristic curves of multivariable logistic regression models based on LASSO-selected variables. The red, blue, and green curves represent the main analysis (excluding malignancy- and autoimmune-related indications), Sensitivity Analysis A (excluding malignancy-related indications), and Sensitivity Analysis B (excluding immune-related indications), respectively. The AUC and corresponding 95% CI are shown for each model, reflecting their discriminative performance. AUC, area under the curve; CI, confidence interval.

A total of 71 candidate variables—including patient age, sex, and 69 drugs identified as positive signals from disproportionality analysis—were included in the main analysis (**Supplementary Table S10**). LASSO regression was applied for variable selection. Using the optimal regularization parameter (λ_min), 62 variables were retained, yielding an AUC of 0.815 (95% CI: 0.775–0.856). Under the more conservative λ_1se criterion, 15 variables were retained with an AUC of 0.702 (95% CI: 0.664–0.741) (**Figures 5A, B**; **Supplementary Table S11**). The 62 variables selected by λ_min were subsequently included in a multivariable logistic regression model, maintaining identical predictive performance (AUC = 0.815, 95% CI: 0.775–0.856) (**Figure 6**; **Supplementary Table S12**).

To assess the robustness of our findings, two sensitivity analyses were conducted. Sensitivity analysis A, which excluded cases with malignancy-related indications, yielded 74 candidate variables (**Supplementary Table S13**). LASSO with λ_min retained 34 variables (AUC = 0.792, 95% CI: 0.757–0.828) (**Figures 5C, D**; **Supplementary Table S14**), whereas λ_1se retained 25 variables (AUC = 0.775, 95% CI: 0.739–0.812). The multivariable logistic regression based on the λ_min-selected variables yielded the same AUC of 0.792 (95% CI: 0.757–0.828) (**Figure 6**; **Supplementary Table S15**). Sensitivity analysis B, which excluded cases with immune-related indications, resulted in 91 candidate variables (**Supplementary Table S16**). Under λ_min, 49 variables were selected (AUC = 0.775, 95% CI: 0.737–0.814), while λ_1se retained 37 variables (AUC = 0.773, 95% CI: 0.741–0.806) (**Figures 5E, F**; **Supplementary Table S17**). Multivariable logistic regression based on the λ_min-selected variables achieved an AUC of 0.776 (95% CI: 0.738–0.815) (**Figure 6**; **Supplementary Table S18**).

**Figure 7:**
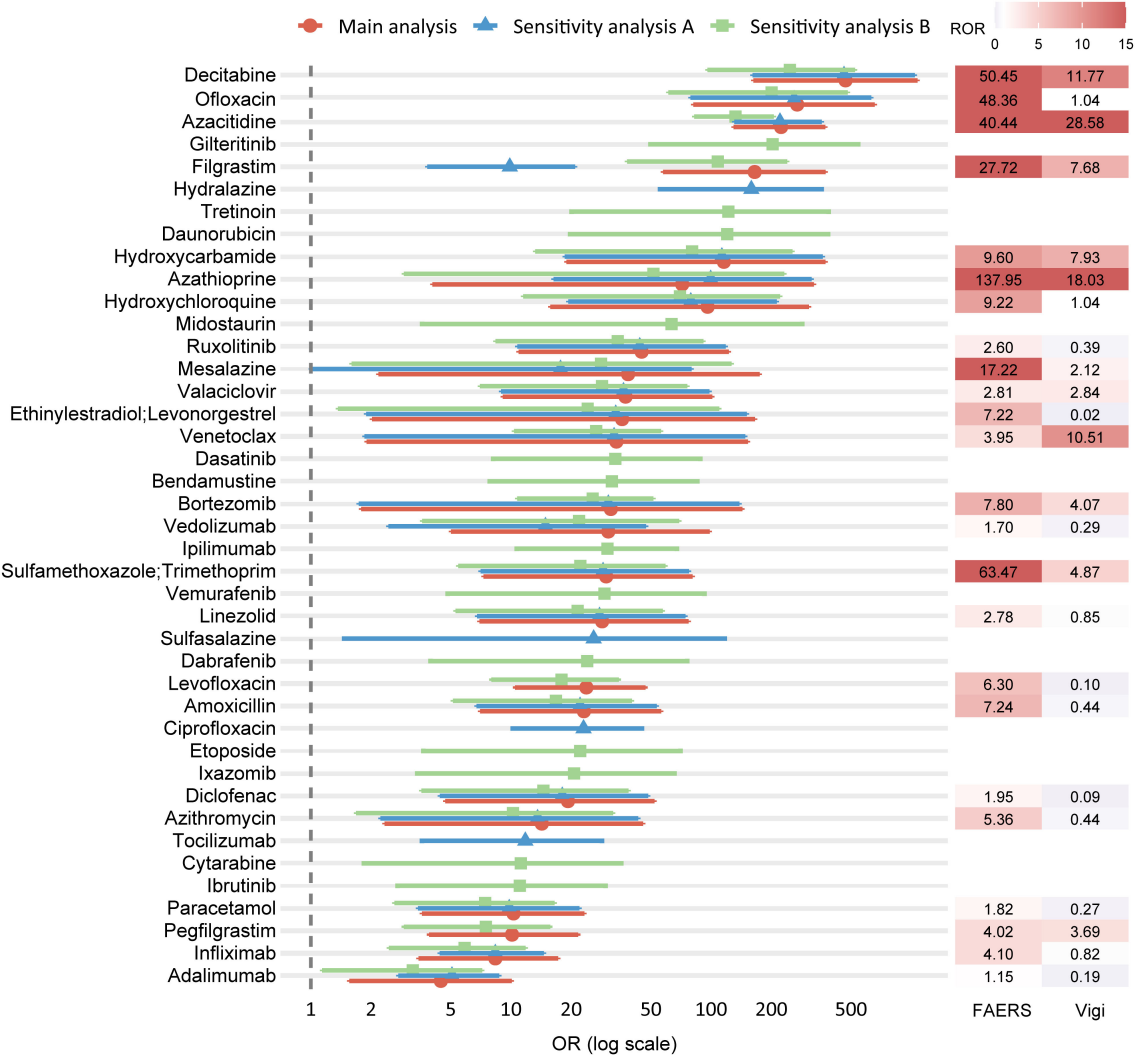
Forest plot of ORs from the main and sensitivity analyses, with corresponding RORs from FAERS and VigiBase. Each row represents a drug retained as statistically significant in multivariable logistic regression. The main analysis (red circles) excluded reports with malignancy- or autoimmune-related indications; Sensitivity analysis A (blue triangles) excluded only malignancy-related indications; Sensitivity analysis B (green squares) excluded only autoimmune-related indications. Horizontal bars indicate 95% CIs, and ORs are presented on a logarithmic scale. The right panel shows the corresponding ROR values from FAERS (left column) and VigiBase (right column), with color intensity proportional to magnitude. ORs, odds ratios; ROR, reporting odds ratio; CI, confidence interval; FAERS, U.S. Food and Drug Administration Adverse Event Reporting System.

Forest plots were constructed to visualize odds ratios (ORs) and 95% CIs for variables retained in the multivariable logistic regression models, highlighting those with statistically significant associations (adjusted *P* < 0.05 and lower bound of 95% CI ≥ 1). In the main analysis, 24 drugs were significantly associated with DISS, most of which belonged to antineoplastic and immunomodulating agents (11/24) and anti-infectives for systemic use (6/24) (**Figure 7**). These included immunosuppressants (azathioprine, adalimumab, infliximab), hematologic agents (azacitidine, decitabine, venetoclax), colony-stimulating factors (filgrastim, pegfilgrastim), and various anti-infectives (sulfamethoxazole/trimethoprim, levofloxacin, amoxicillin, azithromycin, ofloxacin). Additionally, common nonsteroidal anti-inflammatory drugs (NSAIDs) such as paracetamol and diclofenac were retained. Sensitivity analysis A (excluding malignancy-related indications) identified 26 significant drug signals, with a similar ATC class distribution, and additionally highlighted ciprofloxacin, sulfasalazine, tocilizumab, and hydralazine (**Figure 7**). Sensitivity analysis B (excluding immune-related indications) identified 37 significant drugs, further expanding the list to include cytarabine, gilteritinib, ibrutinib, ipilimumab, dasatinib, tretinoin, bendamustine, etoposide, and midostaurin. Notably, the forest plot demonstrates substantial overlap across the three analyses, with a consistent core group of drugs—decitabine, ofloxacin, azacitidine, filgrastim, hydroxycarbamide, hydroxychloroquine, azathioprine, ruxolitinib, mesalazine, valaciclovir, ethinylestradiol/levonorgestrel, venetoclax, bortezomib, vedolizumab, sulfamethoxazole/trimethoprim, linezolid, amoxicillin, diclofenac, azithromycin, paracetamol, infliximab, and adalimumab—retained as significant signals across all models (**Figure 7**). This high degree of concordance underscores the robustness of the identified associations despite variations in exclusion criteria. This consistency underscores the robustness of the identified signals despite varying exclusion criteria.

### 3.6 Cross-database consistency assessment, literature review, and label-based confirmation

To strengthen the credibility of the signals identified in the main analysis, external assessment was conducted using three independent sources. Disproportionality analysis in VigiBase identified 10 drugs as positive signals, including azacitidine, azathioprine, bortezomib, pegfilgrastim, sulfamethoxazole/trimethoprim, decitabine, filgrastim, mesalazine, hydroxycarbamide, valaciclovir, and venetoclax—as positive signals, defined by at least 3 reported cases and a lower 95% CI of ROR exceeding 1 (**Figure 7**; **Supplementary Table S19**). A targeted literature review was performed to assess prior documentation of DISS for each of the 24 drugs identified in the main analysis. Key contextual information was extracted, including the primary indication, time to onset, diagnostic certainty, concomitant medications, and publication type. 15 drugs had prior reports in case studies or observational research (**Table 2**). Finally, regulatory product labels from the FDA and EMA were reviewed. Among the 24 drugs, 9 included SS in the adverse reaction sections of their prescribing information (**Supplementary Table S20**).

**Table 2:** Summary of literature evidence for DISS-associated drug signals identified in the main analysis.

Drug name	Main disease	Time-to-onset	DISS or comorbid disease?	Concomitant medication	Study type	Reference
Sulfamethoxazole/Trimethoprim	Hodgkin’s lymphoma (post-autologous bone, marrow transplantation); Acute diarrhea with high fever	7 days	Confirmed DISS	None	Case report and literature review	(4)
Azacitidine	AML	4 months	Confirmed DISS	None	Case report	(10)
Adalimumab	CD	5 days after 2^nd^ injection	Temporal relationship suggests causality	Unclear	Case report	(11)
Infliximab	CD	Several months	Unclear	Azathioprine	Case report	(12)
Azathioprine	IBD (76% of cases)	5–28 days (mean 13.3 days)	Confirmed DISS	None	Retrospective analysis of 3 cases and literature review (17 cases)	(13)
Filgrastim	Not specified	Not specified	Confirmed DISS	None	Review	(14)
Pegfilgrastim	Neutropenia induced by ziprasidone	Not specified	Confirmed DISS	None	Case report	(15)
Levofloxacin	Respiratory tract infection	7 days	Confirmed DISS	None	Case report	(16)
Amoxicillin	Acute tonsillitis	7 days	Confirmed DISS	None	Case report	(16)
Paracetamol	Post-op facial fracture	7 days	Confirmed DISS	Codeine	Case report	(17)
Diclofenac	Right knee injury	8–10 hours	Confirmed DISS	None	Case report	(18)
Ruxolitinib	Post-essential thrombocythaemia myelofibrosis	Not specified	Unclear	None	Case report	(19)
Azithromycin	None	None	None	None	None	None
Mesalazine	IBD	3 days	Confirmed DISS	None	Case report	(20)
Decitabine	MDS (refractory anemia type)	14 days after the start of the second treatment cycle (5 days of administration in each 7-day cycle	Confirmed DISS	None	Case report	(21)
Hydroxychloroquine	HIV with chronic parvovirus B19; Pancytopenia; Arthralgia	14 days	Confirmed DISS	None	Case report and literature review	(22)
Ofloxacin	CD	3 days	Confirmed DISS	None	Case report	(23)
Vedolizumab	CD	2 hours	Unclear	None	Case report	(24)
Bortezomib	Multiple myeloma	Cycle 8 Day 1 of 21-day cycle (1; 3; 8;11 use drug)	Confirmed DISS	None	Case report	(25)
Hydroxycarbamide	None	None	None	None	None	None
Linezolid	None	None	None	None	None	None
Valaciclovir	MDS; AML; suspected herpes zoster	Unknown	Unclear	Thalidomide, antibiotics (unspecified)	Case report	(26)
Ethinylestradiol/Levonorgestrel	None	Several weeks	Confirmed DISS	None	Case report	(27)
Venetoclax	None	None	None	None	None	None

DISS, drug-induced Sweet’s syndrome; AML, acute myeloid leukemia; CD, Crohn’s disease; IBD, Inflammatory bowel disease; MDS, myelodysplastic syndrome.

## 4 Discussion

DISS is an uncommon but clinically significant neutrophilic dermatosis, typically characterized by acute febrile onset and potential systemic involvement (5). However, early recognition remains challenging due to its heterogeneous clinical manifestations and the broad spectrum of implicated drugs. In the present study, a comprehensive pharmacovigilance analysis was conducted using FAERS data from 2004 to 2024 to clarify drug-associated risks. A total of 2,018 eligible reports were identified, involving 342 suspected drugs. Disproportionality analysis was employed as the primary signal detection method, resulting in 90 drugs disproportionately reported in association with DISS. To reduce confounding from SS-related comorbidities, the main analysis was restricted to reports without malignancy- or immune-related indications. Sensitivity analyses were subsequently performed by reintroducing these conditions. Across all models, consistent results were obtained through LASSO regression and multivariable logistic analysis, with substantial overlap in identified signals, thereby reinforcing the robustness and specificity of the associations. External validation was further conducted using the VigiBase database, supplemented by a targeted literature review and product label examination. This multi-step approach not only confirmed previously recognized associations but also revealed underreported or novel drug-related risks, underscoring the utility of pharmacovigilance data in characterizing the safety profile of DISS.

Regarding the baseline characteristics of DISS, our analysis revealed that the majority of cases occurred in middle-aged and older adults, with a marked female predominance—an epidemiological pattern consistent with previous reports (28, 29). Due to a high proportion of missing data on body weight in the FAERS database, further evaluation of constitutional risk factors was limited. Nearly half of the reports were submitted by medical doctors, and a disproportionately number originated from the United States. This geographic bias likely reflects reporting bias inherent to the FAERS database, as well as differences in pharmacovigilance systems, drug accessibility, and healthcare practices across countries. The most common underlying indications were inflammatory bowel disease (IBD; including CD and UC), hematologic malignancies (such as AML, plasma cell myeloma, and MDS), and RA. These conditions have been well-documented: SS is recognized as a cutaneous extraintestinal manifestation (EIM) of IBD (30), AML and MDS are established predisposing factors (31), and SS manifestations have been observed in RA (32). Given these associations, the potential confounding effects of underlying diseases and their treatments should be carefully considered when interpreting positive drug signals in DISS. In line with the nature of SS as an idiopathic, chronic systemic inflammatory response syndrome, most DISS cases in our cohort were associated with prolonged hospitalization or other non-life-threatening complications, while fatal or disabling outcomes were rare. The majority of cases occurred within 0–30 days after drug exposure, and clinical improvement was commonly observed after discontinuation of the suspected agent (33). Systemic corticosteroids are generally considered the first-line therapy, whereas potassium iodide or colchicine may serve as alternative options for patients with contraindications to corticosteroids (3).

DISS-associated drugs span a broad spectrum of pharmacological categories. Antineoplastic and immunomodulating agents accounted for the highest number of cases and the greatest diversity of implicated drugs. These agents may contribute to the pathogenesis of SS through disruption of immune homeostasis, cytokine dysregulation, and enhanced neutrophil maturation and proliferation (6). Anti-infective agents, including those for systemic and gastrointestinal use, also constituted a substantial proportion of DISS cases. The proposed mechanism involves cytokine-mediated hypersensitivity reactions (26) triggered either by infectious pathogens, such as *Yersinia enterocolitica* (34), or by the pharmacological activity of certain antibiotics (33), including sulfamethoxazole/trimethoprim (4) and ciprofloxacin (35). However, distinguishing infection-related from drug-induced remains a challenge with spontaneous report data. Several other drug classes were also frequently reported, including the antihypertensive hydralazine (36), the diuretic furosemide (37), the gastrointestinal agent mesalazine (20), the antiepileptic drug gabapentin (38) and the analgesic acetaminophen (17). Despite unclear mechanisms, growing case-based evidence suggests their potential role in DISS and highlights the need for further investigation.

To address potential confounding factors, a structured analytical approach was employed, comprising a main analysis and two complementary sensitivity analyses. Following initial signal detection through disproportionality analysis, drugs with positive associations—along with age and sex—were entered into LASSO regression, followed by multivariable logistic regression. In the main analysis, which excluded reports involving malignancy- or immune-related indications, 24 drugs were found to be significantly associated with DISS. The two sensitivity analyses, which reintroduced each exclusion criterion independently, identified 26 and 37 significant signals, respectively. Notably, 22 drugs were consistently detected across all three models, indicating robust and stable associations unlikely to be driven by indication-related bias.

To further support our findings, a cross-database consistency assessment was performed in VigiBase, where 10 of the 24 drugs identified in the main model were observed as positive signals. This partial overlap may reflect complementary signal detection across pharmacovigilance systems, adding contextual value to signal interpretation. However, this consistency should be interpreted with caution, as previous comparative studies have reported considerable redundancy among international pharmacovigilance databases such as EudraVigilance Data Analysis System (EVDAS), FAERS, and VigiBase, with signal overlap rates ranging from 85% to 97% (39). In addition, 15 drugs had prior documentation in published case reports or reviews, and 9 drugs explicitly listed SS in the adverse reaction section of their regulatory product labels. Taken together, these external sources provide supportive—but not confirmatory—evidence, which enhances the plausibility of the detected associations and may help guide future mechanistic research and clinical risk evaluation.

In the main analysis, we identified 24 drugs associated with DISS. Among anti-infective agents for systemic use, sulfamethoxazole/trimethoprim, levofloxacin, and amoxicillin (16) have been consistently reported in the literature as associated with SS, while ofloxacin (23) has been sporadically implicated. Notably, valacyclovir, linezolid, and azithromycin were also identified as positive signals, suggesting previously unrecognized role in DISS. More than half of the identified drugs were classified as antineoplastic and immunomodulating agents. Among hypomethylating agents commonly used to treat hematologic malignancies such as MDS, azacitidine has frequently been associated with DISS (10), while decitabine has recently emerged as a potential trigger (21), with its underlying mechanisms still unclear. G-CSF agents, such as filgrastim and pegfilgrastim, are the most commonly reported drugs associated with DISS. They are thought to excessively amplify the inflammatory response by promoting the proliferation, maturation, and activation of neutrophils, thereby substantially increasing the risk of SS following exogenous administration (6). Ruxolitinib, a Janus kinase (JAK) 1/2 inhibitor, has also been recently implicated in the onset of SS, particularly in patients with myelofibrosis (19). Azathioprine has been widely recognized as a significant precipitating factor, especially in patients with steroid-dependent or refractory IBD (13). In these cases, SS is often misdiagnosed as a flare of the underlying disease (13); however, oral rechallenge tests have substantiated a causal relationship between azathioprine and DISS (40), although the exact immunopathological mechanisms remain unclear. Additionally, mesalamine, a first-line therapeutic agent for IBD, has been documented to induce SS cutaneous manifestations within just 3 days of treatment initiation, with subsequent rapid progression to myopericarditis, resulting in multi-system involvement (20). Proteasome inhibitors, such as bortezomib (25), have also been associated with SS, possibly via inhibition of the β5 and β5i proteasomal subunits, leading to dysregulation of IL-6 and IFN-γ signaling pathways (41). Other newly identified drugs with currently unclear mechanisms included hydroxycarbamide (an antimetabolite), venetoclax (a B-cell lymphoma-2 inhibitor). Within the category of antiparasitic products, insecticides, and repellents, hydroxychloroquine (22) has been sporadically reported, although the precise mechanism remains to be clarified. Among NSAIDs, diclofenac was identified in the present study. Although reports remain limited (18), association between other NSAIDs, such as celecoxib (42), and SS has been documented, suggesting a potential class effect. Paracetamol was also identified but only in isolated cases, and current evidence remains limited. Oral contraceptives containing ethinylestradiol and levonorgestrel have been previously reported to increase the risk of SS (27). The proposed mechanism involves reduced neutrophil apoptosis during hormonal treatment or pregnancy, resulting in elevated counts of neutrophils, monocytes, and lymphocytes (27).

This study identified several monoclonal antibodies associated with DISS, including adalimumab and infliximab (both targeting TNF-α) as well as vedolizumab (targeting integrins). Although multiple case reports have implicated adalimumab in the induction of SS (11, 43), other studies have suggested its potential therapeutic efficacy in refractory or immune-mediated cases—particularly those associated with IBD (44, 45). Similarly, Infliximab has also been associated with both the induction (12, 46) and treatment (47, 48) of SS in clinical settings. Moreover, vedolizumab has been reported to both induce (24) and treat (49) SS in patients with UC. This paradoxical “inductive/therapeutic dual role” appears to be particularly prominent among anti-TNF-α agents (5, 50). TNF-α is a key pro-inflammatory cytokine implicated in the pathogenesis of several autoimmune and inflammatory diseases. Elevated TNF-α levels contribute to chronic immune activation and tissue damage in IBD and RA, justifying the use of anti-TNF-α therapies (51). Furthermore, TNF-α has been shown to promote neutrophil recruitment and activation in SS, suggesting an overlap in inflammatory pathways (52, 53). Anti-TNF-α agents may influence SS development through mechanisms involving neutrophil dysfunction or imbalances in cell-mediated immune regulation (54). As such, the role of such agents in SS should be understood as complex and highly individualized clinical phenomenon. These findings highlight the challenges of translating pharmacovigilance data into clinical practice and underscore the importance of multifactorial risk assessments that consider disease background, treatment indications, and individual susceptibility when evaluating DISS signals.

Additionally, several drugs previously implicated in case reports or small series, including minocycline, celecoxib, and thalidomide (33), were not retained after LASSO selection and multivariable analysis. Potential explanations include low reporting frequencies, variations in patient characteristics, and the application of stricter model selection criteria.

To enhance clinical interpretability, we conducted a supplementary disproportionality analysis based on reports submitted by medical doctors, which are generally considered to possess higher diagnostic specificity and may reflect confirmed clinical assessments. Among 215 evaluated drugs, 57 met the disproportionality threshold, and the distribution of positive signals demonstrated strong concordance with the full dataset. Although several drugs with high reporting frequencies—such as lenalidomide, etanercept, ruxolitinib, dexamethasone, paracetamol, methotrexate, tocilizumab, lamotrigine, dupilumab, and amlodipine—did not meet the threshold for a positive signal in this subset, this may, in part, be attributable to the limited number of physician-submitted reports, which could reduce the statistical power to detect disproportionality. Notably, two of these drugs (lenalidomide and ruxolitinib) were confirmed as positive signals in the main analysis. Furthermore, six of the ten most frequently reported but non-signaling drugs in this subset had previously been documented in the literature, lending additional support to their clinical relevance. This observation suggests that drugs frequently reported by clinicians, even if not statistically flagged in restricted analyses, may still represent clinically suspected signals and warrant further attention. These findings underscore the potential value of medical doctor–reported data in guiding signal prioritization for pharmacovigilance and follow-up, particularly in the context of rare adverse events such as DISS.

This study has several notable strengths. It utilized a large, publicly available pharmacovigilance database (FAERS), capturing a broad and heterogeneous population over a 20-year period. The substantial sample size enabled the detection of rare adverse events such as DISS and supported robust signal estimation based on real-world reporting patterns. A key methodological strength lies in the implementation of a comprehensive, multi-step analytical framework. Disproportionality analysis served as an initial screening tool to identify candidate drug signals, which were then refined through LASSO regression and confirmed via multivariable logistic analysis. Specifically, LASSO regression facilitated efficient variable selection by reducing the number of candidate signals and minimizing the risk of overfitting. The subsequent multivariable analysis enabled adjustment for confounding factors and identification of independently associated drug signals, thereby strengthening the internal validity of the results. Moreover, to further reduce bias from underlying conditions commonly associated with SS, the main analysis excluded reports involving malignancy- or immune-related indications. Sensitivity analyses that reintroduced these exclusions yielded consistent findings, reinforcing the robustness and specificity of the associations. The validity of the results was further supported by external evidence. Cross-database validation using VigiBase enhanced the reliability of signal detection across independent pharmacovigilance systems. Literature review provided additional clinical support for many of the observed associations, while product label examination highlighted the potential of post-marketing surveillance data to uncover underrecognized or unlabeled adverse events. In addition, co-medication patterns were explored using network visualization to reflect real-world polypharmacy, and time-to-onset analysis offered clinically relevant insights into the temporal profile of DISS across different drug classes. Collectively, this study presents a rigorous, data-driven, and externally validated pharmacovigilance assessment, offering a scalable framework for investigating rare but clinically important drug-related safety signals.

Despite the strengths of this study, several limitations should be acknowledged. First, as with all analyses based on spontaneous reporting systems, the FAERS database is subject to well-established biases, including underreporting, duplicate and selective reporting, lack of denominator data, and the absence of standardized event adjudication. These limitations hinder accurate estimation of incidence rates and constrain the ability to assess the absolute risk of adverse events. Second, the dataset lacks detailed patient-level clinical information, such as comorbidities, concomitant medications, disease severity, and time-to-onset data. The absence or incompleteness of these variables reduced interpretability in certain domains and limited the ability to perform more granular subgroup analyses or risk stratification. In particular, cases not submitted by medical doctors may involve diagnostic uncertainty, as they are less likely to reflect confirmed clinical assessments. Third, the core analytical method—disproportionality analysis—detects statistical associations rather than establishing biological or causal relationships. Signal detection can be influenced by external factors such as reporting behavior, masking effects, and patterns of polypharmacy. This study is exploratory in nature and reflects the inherent limitations of pharmacovigilance research; the analyses were intended to generate hypotheses rather than to confirm causality between drug exposure and adverse outcomes. Although LASSO regression was used prior to multivariable modeling to reduce dimensionality and address multicollinearity, the selection of covariates remained constrained by the structure of the FAERS dataset. Fourth, while a subset analysis of medical doctor–submitted reports was performed to enhance clinical interpretability, this approach remains subject to the inherent limitations of spontaneous reporting data. Frequently reported drugs that did not meet the disproportionality threshold should be regarded as clinically suspected signals rather than confirmed associations, and their interpretation requires caution in the absence of corroborating evidence. Fifth, despite efforts to mitigate confounding through indication-based exclusions and sensitivity analyses, residual confounding—particularly confounding by indication—cannot be fully excluded. Finally, although external sources such as VigiBase were incorporated to support the main findings, potential overlap with FAERS case reports may limit the independence. Thus, cross-database consistency signals should be interpreted with caution. Looking ahead, future research should prioritize well-designed cohort studies and rigorously documented case series, which are essential for confirming causality, elucidating underlying mechanisms, and guiding clinical risk assessment.

## 5 Conclusion

This study systematically identified and characterized drugs associated with DISS by leveraging the large pharmacovigilance database FAERS, integrating disproportionality analysis, LASSO regression, and multivariable modeling. Several previously recognized drugs, such as sulfamethoxazole/trimethoprim; azacitidine; azathioprine; filgrastim; pegfilgrastim; levofloxacin; amoxicillin; paracetamol; diclofenac; mesalazine; decitabine; hydroxychloroquine; ofloxacin; bortezomib; ethinylestradiol and levonorgestrel were confirmed, while novel signals, including azithromycin; hydroxycarbamide; linezolid and venetoclax were newly identified. Despite the inherent limitations of spontaneous reporting systems and retrospective observational designs, these findings provide a valuable foundation for future prospective studies and mechanistic investigations. This study represents a comprehensive effort to delineate the drug risk landscape of DISS using real-world data, providing new insights into its pharmacological triggers and laying the groundwork for improving pharmacovigilance, early recognition, and management of DISS in clinical practice.

## Data Availability

All data from FAERS are publicly accessible through the FDA website (https://fis.fda.gov/extensions/FPD-QDE-FAERS/FPD-QDE-FAERS.html). Access to VigiBase data was obtained under license from the UMC. VigiBase is not publicly available; researchers interested in accessing VigiBase must apply through the UMC (https://who-umc.org/). Further information about the datasets used in this study is available from the corresponding author upon reasonable request.
