# Supplementary material for: Drug-induced Sweet’s syndrome: pharmacovigilance insights from FAERS with a cross-database consistency assessment in VigiBase via LASSO and multivariable logistic regression
Source: Front Immunol. 2025 Sep 23;16:1622736. doi: 10.3389/fimmu.2025.1622736 (PMC12502736; doi:10.3389/fimmu.2025.1622736)
Supplement: Supplementary file 1 [file Table1.docx]

Supplementary Material

# Supplementary Data

**Supplementary Figures**

**Supplementary Figure 1.** Distribution of reported primary indications among drug-induced Sweet’s syndrome cases based on system-level classification.

**Supplementary Figure 2.** Co-occurrence network of drugs reported in association with drug-induced Sweet’s syndrome.

**Supplementary Tables**

**Supplementary Table 1.** Two-by-two contingency table used for disproportionality analysis.

**Supplementary Table 2.** Most frequently reported drugs associated with drug-induced Sweet’s syndrome in the six leading reporting countries.

**Supplementary Table 3.** Disproportionality analysis of drugs reported in association with drug-induced Sweet’s syndrome in the FAERS database.

**Supplementary Table 4.** Disproportionality analysis of drugs reported in association with drug-induced Sweet’s syndrome in medical doctor–submitted cases from the FAERS database.

**Supplementary Table 5.** ATC Level 1 and Level 2 classification of drugs reported in association with drug-induced Sweet’s syndrome.

**Supplementary Table 6.** Chi‑square analysis of sex distribution across 14 ATC Level 1 drug classes associated with drug-induced Sweet’s syndrome.

**Supplementary Table 7.** Chi‑square analysis of age‑group distribution across 14 ATC Level 1 drug classes associated with drug-induced Sweet’s syndrome.

**Supplementary Table 8.** Linear regression trends (2004–2024) in drug-induced Sweet’s syndrome reports for 14 ATC Level 1 drug classes.

**Supplementary Table 9.** Frequently reported drugs by medical doctors without disproportionality signals in FAERS: supplementary assessment using VigiBase, published literature, and product labels.

**Supplementary Table 10.** Disproportionality analysis results of candidate drugs in the main analysis.

**Supplementary Table 11.** Main analysis (malignancy‑/immune‑related indications excluded): LASSO selections at λ_min and λ_1se.

**Supplementary Table 12.** Main analysis (malignancy‑/immune‑related indications excluded): Multivariable logistic regression results for λ_min‑selected variables.

**Supplementary Table 13.** Disproportionality analysis results of candidate drugs in the Sensitivity analysis A (excluding malignancy-related indications).

**Supplementary Table 14.** Sensitivity Analysis A (malignancy‑related indications excluded): LASSO selections at λ_min and λ_1se.

**Supplementary Table 15.** Sensitivity Analysis A (malignancy‑related indications excluded): Multivariable logistic regression results for λ_min‑selected variables.

**Supplementary Table 16.** Disproportionality analysis results of candidate drugs in the Sensitivity analysis B (excluding immune-related indications).

**Supplementary Table 17.** Sensitivity Analysis B (immune‑related indications excluded): LASSO selections at λ_min and λ_1se.

**Supplementary Table 18.** Sensitivity Analysis B (immune‑related indications excluded): Multivariable logistic regression results for λ_min‑selected variables.

**Supplementary Table 19.** VigiBase-based disproportionality analysis of drugs identified in the main analysis.

**Supplementary Table 20.** Product‑label documentation of Sweet’s syndrome for drugs identified in the main analysis (FDA/EMA sources).

# Supplementary Figures and Tables

## Supplementary Figures


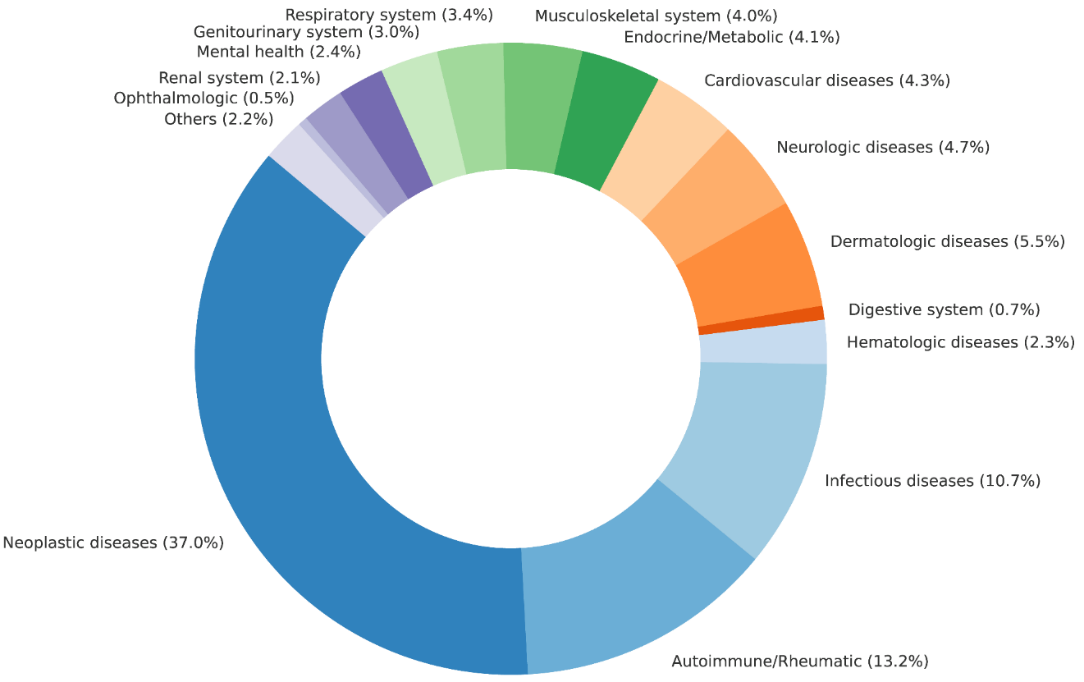


**Supplementary Figure 1. Distribution of reported primary indications among DISS cases based on system-level classification.** This donut chart illustrates the proportion of drug-induced Sweet's syndrome reports categorized by the primary indication according to system-level disease classification.


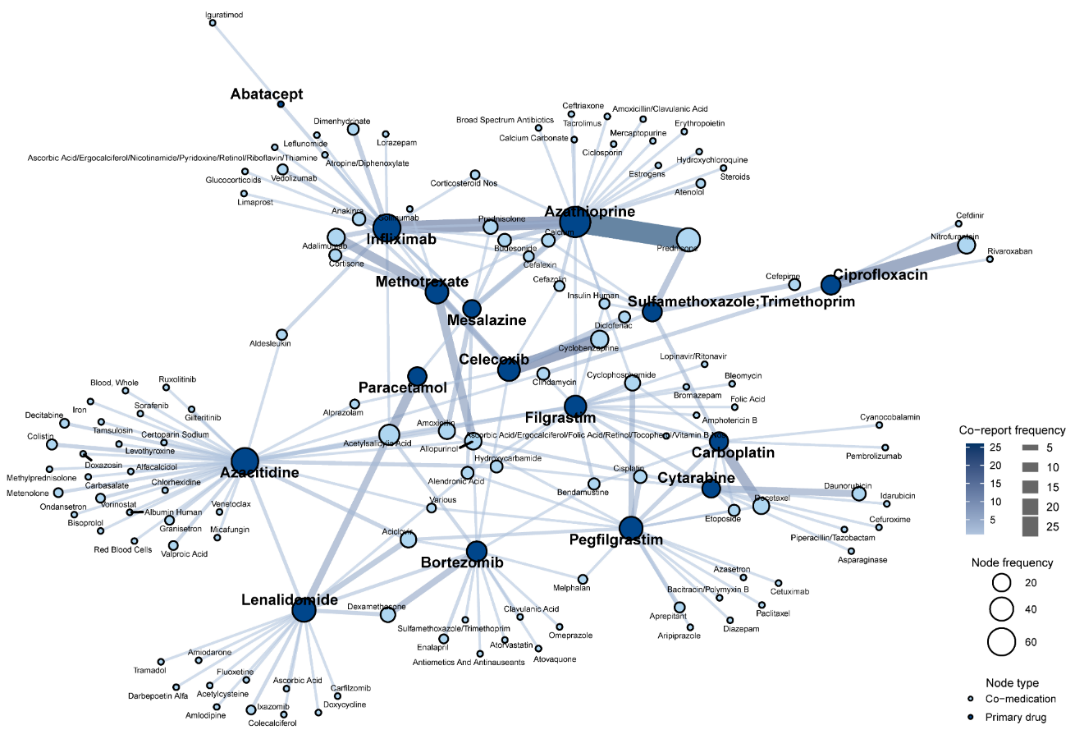


**Supplementary Figure 2. Co-occurrence network of drugs reported in association with drug-induced Sweet’s syndrome.** Each node represents an individual drug, with node size proportional to its frequency in DISS reports. Edges between nodes indicate that the drugs were co-reported within the same case.

## Supplementary Tables

**Supplementary Table 1.** Two-by-two contingency table used for disproportionality analysis.

|  | **Target adverse events** | **All other adverse events** |
| --- | --- | --- |
| **Target drug** | a | b |
| **All other drugs** | c | d |

**Supplementary Table 2.** Most frequently reported drugs associated with drug-induced Sweet’s syndrome in the six leading reporting countries.

| **Country** | **Drug** | **Number of Cases** |
| --- | --- | --- |
| United States | **Total Reports** | **720** |
|  | Sulfamethoxazole;Trimethoprim | 62 (8.61%) |
|  | Hydralazine | 44 (6.11%) |
|  | Azathioprine | 34 (4.72%) |
|  | Lenalidomide | 31 (4.31%) |
|  | Azacitidine | 25 (3.47%) |
|  | Furosemide | 19 (2.64%) |
|  | Pegfilgrastim | 19 (2.64%) |
|  | Adalimumab | 18 (2.50%) |
|  | Celecoxib | 17 (2.36%) |
|  | Ibuprofen | 15 (2.08%) |
| France | **Total Reports** | **243** |
|  | Hydroxychloroquine | 13 (5.35%) |
|  | Bortezomib | 11 (4.53%) |
|  | Infliximab | 11 (4.53%) |
|  | Paracetamol | 11 (4.53%) |
|  | Adalimumab | 10 (4.12%) |
|  | Aripiprazole | 8 (3.29%) |
|  | Azacitidine | 8 (3.29%) |
|  | Anastrozole | 6 (2.47%) |
|  | Lansoprazole | 6 (2.47%) |
|  | Amlodipine | 5 (2.06%) |
| Canada | **Total Reports** | **143** |
|  | Infliximab | 25 (17.48%) |
|  | Methotrexate | 23 (16.08%) |
|  | Ciprofloxacin | 13 (9.09%) |
|  | Leflunomide | 8 (5.59%) |
|  | Vedolizumab | 5 (3.50%) |
|  | Filgrastim | 4 (2.80%) |
|  | Gilteritinib | 4 (2.80%) |
|  | Prednisolone | 4 (2.80%) |
|  | Adalimumab | 3 (2.10%) |
|  | Doxycycline | 3 (2.10%) |
| Spain | **Total Reports** | **102** |
|  | Gabapentin | 15 (14.71%) |
|  | Adalimumab | 8 (7.84%) |
|  | Azithromycin | 7 (6.86%) |
|  | Bortezomib | 5 (4.90%) |
|  | Infliximab | 5 (4.90%) |
|  | Azacitidine | 4 (3.92%) |
|  | Enalapril | 4 (3.92%) |
|  | Hydrochlorothiazide | 4 (3.92%) |
|  | Glycopyrronium; Indacaterol | 3 (2.94%) |
|  | Cytarabine | 2 (1.96%) |
| Japan | **Total Reports** | **94** |
|  | Azacitidine | 12 (12.77%) |
|  | Gilteritinib | 5 (5.32%) |
|  | Ixazomib | 5 (5.32%) |
|  | Pegfilgrastim | 5 (5.32%) |
|  | Prednisolone | 4 (4.26%) |
|  | Tretinoin | 4 (4.26%) |
|  | Clindamycin | 3 (3.19%) |
|  | Darbepoetin Alfa | 3 (3.19%) |
|  | Dexamethasone | 3 (3.19%) |
|  | Lenalidomide | 3 (3.19%) |
| United Kingdom | **Total Reports** | **92** |
|  | Adalimumab | 10 (10.87%) |
|  | Sulfamethoxazole; Trimethoprim | 6 (6.52%) |
|  | Clozapine | 5 (5.43%) |
|  | Cytarabine | 5 (5.43%) |
|  | Propylthiouracil | 5 (5.43%) |
|  | Naproxen | 4 (4.35%) |
|  | Erlotinib | 3 (3.26%) |
|  | Infliximab | 3 (3.26%) |
|  | Minoxidil | 3 (3.26%) |
|  | Azathioprine | 2 (2.17%) |

**Supplementary Table 3**. Disproportionality analysis of drugs reported in association with drug-induced Sweet’s syndrome in the FAERS database.

| **Drug** | **a** | **b** | **c** | **d** | **ROR** | **95%CI** |
| --- | --- | --- | --- | --- | --- | --- |
| Azathioprine | 84 | 5858 | 1934 | 18606116 | 137.95 | 110.72-171.89 |
| Sulfamethoxazole; Trimethoprim | 79 | 11939 | 1939 | 18600035 | 63.47 | 50.65-79.54 |
| Adalimumab | 76 | 614099 | 1942 | 17997875 | 1.15 | 0.91-1.44 |
| Infliximab | 76 | 175891 | 1942 | 18436083 | 4.10 | 3.26-5.16 |
| Azacitidine | 69 | 16279 | 1949 | 18595695 | 40.44 | 31.79-51.44 |
| Hydralazine | 50 | 3527 | 1968 | 18608447 | 134.04 | 101.04-177.82 |
| Lenalidomide | 41 | 348520 | 1977 | 18263454 | 1.09 | 0.80-1.48 |
| Filgrastim | 36 | 12186 | 1982 | 18599788 | 27.72 | 19.93-38.57 |
| Pegfilgrastim | 35 | 81313 | 1983 | 18530661 | 4.02 | 2.88-5.62 |
| Methotrexate | 32 | 129729 | 1986 | 18482245 | 2.30 | 1.62-3.26 |
| Ciprofloxacin | 30 | 37851 | 1988 | 18574123 | 7.41 | 5.16-10.62 |
| Celecoxib | 28 | 40606 | 1990 | 18571368 | 6.44 | 4.43-9.35 |
| Bortezomib | 27 | 32306 | 1991 | 18579668 | 7.80 | 5.33-11.40 |
| Levofloxacin | 25 | 36991 | 1993 | 18574983 | 6.30 | 4.25-9.35 |
| Mesalazine | 23 | 12456 | 1995 | 18599518 | 17.22 | 11.41-25.98 |
| Minocycline | 23 | 5388 | 1995 | 18606586 | 39.81 | 26.37-60.11 |
| Furosemide | 22 | 23551 | 1996 | 18588423 | 8.70 | 5.71-13.25 |
| Gabapentin | 21 | 64576 | 1997 | 18547398 | 3.02 | 1.96-4.64 |
| Ibrutinib | 20 | 66162 | 1998 | 18545812 | 2.81 | 1.81-4.36 |
| Clindamycin | 19 | 14114 | 1999 | 18597860 | 12.52 | 7.97-19.68 |
| Etanercept | 19 | 510868 | 1999 | 18101106 | 0.34 | 0.21-0.53 |
| Paracetamol | 18 | 23114 | 2000 | 18588860 | 1.82 | 1.14-2.89 |
| Amoxicillin | 18 | 11936 | 2000 | 18600038 | 7.24 | 4.55-11.51 |
| Cytarabine | 18 | 2625 | 2000 | 18609349 | 14.02 | 8.81-22.31 |
| Gilteritinib | 18 | 20054 | 2000 | 18591920 | 63.80 | 40.05-101.64 |
| Letrozole | 18 | 91741 | 2000 | 18520233 | 8.34 | 5.24-13.27 |
| Carboplatin | 17 | 43562 | 2001 | 18568412 | 3.62 | 2.25-5.84 |
| Venetoclax | 17 | 79315 | 2001 | 18532659 | 3.95 | 2.45-6.37 |
| Ibuprofen | 17 | 60708 | 2001 | 18551266 | 1.99 | 1.23-3.20 |
| Ruxolitinib | 17 | 39924 | 2001 | 18572050 | 2.60 | 1.61-4.18 |
| Hydroxychloroquine | 16 | 75820 | 2002 | 18536154 | 9.22 | 5.63-15.08 |
| Diclofenac | 16 | 16125 | 2002 | 18595849 | 1.95 | 1.19-3.20 |
| Topotecan | 15 | 3449 | 2003 | 18608525 | 40.40 | 24.29-67.22 |
| Azithromycin | 14 | 24248 | 2004 | 18587726 | 5.36 | 3.17-9.06 |
| Decitabine | 14 | 39324 | 2004 | 18572650 | 50.45 | 29.78-85.46 |
| Clopidogrel | 14 | 2577 | 2004 | 18609397 | 3.30 | 1.95-5.58 |
| Dexamethasone | 14 | 41968 | 2004 | 18570006 | 3.09 | 1.83-5.23 |
| Docetaxel | 13 | 51044 | 2005 | 18560930 | 2.36 | 1.37-4.07 |
| Ipilimumab | 13 | 17402 | 2005 | 18594572 | 6.93 | 4.01-11.96 |
| Amiodarone | 12 | 19942 | 2006 | 18592032 | 5.58 | 3.16-9.84 |
| Amoxicillin; Clavulanic Acid | 11 | 15347 | 2007 | 18596627 | 6.64 | 3.67-12.01 |
| Prednisone | 11 | 16160 | 2007 | 18595814 | 2.89 | 1.60-5.22 |
| Lamotrigine | 11 | 26302 | 2007 | 18585672 | 1.95 | 1.08-3.53 |
| Gemcitabine | 11 | 52070 | 2007 | 18559904 | 3.87 | 2.14-7.01 |
| Rituximab | 11 | 35256 | 2007 | 18576718 | 0.98 | 0.54-1.77 |
| Vedolizumab | 11 | 103697 | 2007 | 18508277 | 1.70 | 0.94-3.08 |
| Doxycycline | 11 | 59735 | 2007 | 18552239 | 6.31 | 3.49-11.41 |
| Capecitabine | 10 | 69167 | 2008 | 18542807 | 1.63 | 0.88-3.04 |
| Dasatinib | 10 | 56585 | 2008 | 18555389 | 3.17 | 1.70-5.90 |
| Imatinib | 10 | 29189 | 2008 | 18582785 | 1.64 | 0.88-3.05 |
| Leflunomide | 10 | 56354 | 2008 | 18555620 | 5.97 | 3.20-11.11 |
| Tocilizumab | 10 | 15524 | 2008 | 18596450 | 1.62 | 0.87-3.02 |
| Aripiprazole | 10 | 56930 | 2008 | 18555044 | 1.34 | 0.72-2.49 |
| Isotretinoin | 9 | 100260 | 2009 | 18511714 | 1.60 | 0.83-3.08 |
| Tretinoin | 9 | 291692 | 2009 | 18320282 | 28.37 | 14.73-54.67 |
| Ofloxacin | 9 | 51891 | 2009 | 18560083 | 48.36 | 25.08-93.24 |
| Ixazomib | 9 | 16070 | 2009 | 18595904 | 5.18 | 2.69-9.98 |
| Mycophenolic acid | 9 | 59681 | 2009 | 18552293 | 1.39 | 0.72-2.68 |
| Dupilumab | 9 | 1724 | 2009 | 18610250 | 0.28 | 0.15-0.54 |
| Clozapine | 9 | 33464 | 2009 | 18578510 | 0.83 | 0.43-1.59 |
| Prednisolone | 9 | 2938 | 2009 | 18609036 | 2.49 | 1.29-4.79 |
| Sulfasalazine | 8 | 14512 | 2010 | 18597462 | 9.84 | 4.91-19.70 |
| Erlotinib | 8 | 40326 | 2010 | 18571648 | 1.83 | 0.92-3.67 |
| Anastrozole | 8 | 619 | 2010 | 18611355 | 5.10 | 2.55-10.22 |
| Propylthiouracil | 8 | 7528 | 2010 | 18604446 | 119.67 | 59.50-240.69 |
| Clarithromycin | 7 | 9585 | 2011 | 18602389 | 3.83 | 1.82-8.05 |
| Fluconazole | 7 | 9476 | 2011 | 18602498 | 6.61 | 3.14-13.88 |
| Allopurinol | 7 | 16884 | 2011 | 18595090 | 6.76 | 3.22-14.19 |
| Bendamustine | 7 | 12905 | 2011 | 18599069 | 6.83 | 3.25-14.36 |
| Dabrafenib | 7 | 3355 | 2011 | 18608619 | 5.02 | 2.39-10.54 |
| Enasidenib | 7 | 9801 | 2011 | 18602173 | 19.31 | 9.19-40.58 |
| Mercaptopurine | 7 | 27454 | 2011 | 18584520 | 25.44 | 12.10-53.49 |
| Lansoprazole | 7 | 2546 | 2011 | 18609428 | 2.36 | 1.12-4.95 |
| Codeine; Paracetamol | 6 | 49731 | 2012 | 18562243 | 17.72 | 7.94-39.52 |
| Vancomycin | 6 | 3132 | 2012 | 18608842 | 2.59 | 1.16-5.77 |
| Levetiracetam | 6 | 11873 | 2012 | 18600101 | 1.05 | 0.47-2.34 |
| Ketoconazole | 6 | 4744 | 2012 | 18607230 | 11.70 | 5.25-26.08 |
| Etoposide | 6 | 10433 | 2012 | 18601541 | 4.67 | 2.10-10.41 |
| Nilotinib | 6 | 52809 | 2012 | 18559165 | 2.15 | 0.97-4.80 |
| Nivolumab | 6 | 25146 | 2012 | 18586828 | 0.86 | 0.39-1.92 |
| Sorafenib | 6 | 69566 | 2012 | 18542408 | 2.95 | 1.32-6.58 |
| Lamivudine | 6 | 25734 | 2012 | 18586240 | 5.32 | 2.39-11.85 |
| Amlodipine | 6 | 64098 | 2012 | 18547876 | 1.11 | 0.50-2.48 |
| Methylprednisolone | 6 | 18791 | 2012 | 18593183 | 2.20 | 0.99-4.91 |
| Naproxen | 6 | 21409 | 2012 | 18590565 | 0.79 | 0.36-1.77 |
| Darbepoetin Alfa | 5 | 82705 | 2013 | 18529269 | 1.24 | 0.51-2.98 |
| Carbamazepine | 5 | 8034 | 2013 | 18603940 | 2.17 | 0.90-5.22 |
| Midostaurin | 5 | 10982 | 2013 | 18600992 | 20.29 | 8.43-48.85 |
| Mitoxantrone | 5 | 21289 | 2013 | 18590685 | 25.38 | 10.54-61.13 |
| Trastuzumab | 5 | 37292 | 2013 | 18574682 | 1.38 | 0.57-3.31 |
| Vemurafenib | 5 | 2595 | 2013 | 18609379 | 4.88 | 2.03-11.73 |
| Bisoprolol | 5 | 23616 | 2013 | 18588358 | 4.21 | 1.75-10.12 |
| Omeprazole | 5 | 70073 | 2013 | 18541901 | 1.19 | 0.49-2.86 |
| Metformin | 5 | 2278 | 2013 | 18609696 | 0.66 | 0.27-1.58 |
| Interferon Beta-1B | 5 | 1821 | 2013 | 18610153 | 1.96 | 0.81-4.70 |
| Peginterferon Alfa-2A | 5 | 38778 | 2013 | 18573196 | 1.47 | 0.61-3.54 |
| Abatacept | 5 | 31372 | 2013 | 18580602 | 0.56 | 0.23-1.34 |
| Anakinra | 5 | 129339 | 2013 | 18482635 | 5.75 | 2.39-13.84 |
| Efalizumab | 5 | 33559 | 2013 | 18578415 | 17.81 | 7.40-42.88 |
| Secukinumab | 5 | 51026 | 2013 | 18560948 | 0.35 | 0.15-0.85 |
| Upadacitinib | 5 | 9474 | 2013 | 18602500 | 0.90 | 0.38-2.17 |
| Enalapril | 4 | 136688 | 2014 | 18475286 | 7.37 | 2.76-19.65 |
| Ramipril | 4 | 10303 | 2014 | 18601671 | 2.09 | 0.79-5.59 |
| Valsartan | 4 | 80928 | 2014 | 18531046 | 1.50 | 0.56-3.99 |
| Deferasirox | 4 | 548 | 2014 | 18611426 | 1.65 | 0.62-4.41 |
| Ceftriaxone | 4 | 49800 | 2014 | 18562174 | 3.59 | 1.34-9.57 |
| Linezolid | 4 | 1146 | 2014 | 18610828 | 2.78 | 1.04-7.42 |
| Piperacillin; Tazobactam | 4 | 22328 | 2014 | 18589646 | 4.22 | 1.58-11.26 |
| Terbinafine | 4 | 32498 | 2014 | 18579476 | 4.14 | 1.55-11.06 |
| Daunorubicin | 4 | 5017 | 2014 | 18606957 | 32.25 | 12.07-86.17 |
| Doxorubicin | 4 | 37281 | 2014 | 18574693 | 1.14 | 0.43-3.03 |
| Everolimus | 4 | 50949 | 2014 | 18561025 | 0.99 | 0.37-2.64 |
| Hydroxycarbamide | 4 | 47002 | 2014 | 18564972 | 9.60 | 3.60-25.62 |
| Idarubicin | 4 | 8135 | 2014 | 18603839 | 32.37 | 12.12-86.47 |
| Pembrolizumab | 4 | 3849 | 2014 | 18608125 | 0.77 | 0.29-2.04 |
| Chloroquine | 4 | 1142 | 2014 | 18610832 | 67.45 | 25.20-180.54 |
| Apixaban | 4 | 13274 | 2014 | 18598700 | 0.27 | 0.10-0.72 |
| Stavudine | 4 | 16689 | 2014 | 18595285 | 32.28 | 12.08-86.24 |
| Valaciclovir | 4 | 48091 | 2014 | 18563883 | 2.81 | 1.05-7.49 |
| Hydrochlorothiazide | 4 | 8757 | 2014 | 18603217 | 4.54 | 1.70-12.12 |
| Glatiramer | 4 | 79773 | 2014 | 18532201 | 0.72 | 0.27-1.93 |
| Certolizumab pegol | 4 | 17636 | 2014 | 18594338 | 0.45 | 0.17-1.21 |
| Ciclosporin | 4 | 1145 | 2014 | 18610829 | 0.74 | 0.28-1.97 |
| Golimumab | 4 | 8914 | 2014 | 18603060 | 0.78 | 0.29-2.09 |
| Pomalidomide | 4 | 73296 | 2014 | 18538678 | 0.46 | 0.17-1.23 |
| Ustekinumab | 4 | 13147 | 2014 | 18598827 | 0.50 | 0.19-1.34 |
| Metronidazole | 4 | 24669 | 2014 | 18587305 | 2.21 | 0.83-5.90 |
| Fentanyl | 3 | 11270 | 2015 | 18600704 | 0.37 | 0.12-1.16 |
| Nitrofurantoin | 3 | 21898 | 2015 | 18590076 | 5.88 | 1.89-18.24 |
| Budesonide | 3 | 85178 | 2015 | 18526796 | 1.31 | 0.42-4.07 |
| Minoxidil | 3 | 23323 | 2015 | 18588651 | 0.47 | 0.15-1.46 |
| Atezolizumab | 3 | 21086 | 2015 | 18590888 | 1.26 | 0.41-3.92 |
| Cladribine | 3 | 139968 | 2015 | 18472006 | 4.79 | 1.54-14.87 |
| Cyclophosphamide | 3 | 5783 | 2015 | 18606191 | 0.93 | 0.30-2.88 |
| Cytarabine; Daunorubicin | 3 | 29837 | 2015 | 18582137 | 18.05 | 5.81-56.08 |
| Fludarabine | 3 | 1535 | 2015 | 18610439 | 3.47 | 1.12-10.78 |
| Trametinib | 3 | 17362 | 2015 | 18594612 | 2.89 | 0.93-8.97 |
| Fumaric Acid | 3 | 3835 | 2015 | 18608139 | 0.26 | 0.08-0.79 |
| Calcium Chloride; Glucose; Magnesium Chloride;Sodium Chloride; Sodium Lactate | 3 | 74008 | 2015 | 18537966 | 0.20 | 0.06-0.61 |
| Glycopyrronium; Indacaterol | 3 | 7977 | 2015 | 18603997 | 8.87 | 2.86-27.55 |
| Omalizumab | 3 | 107902 | 2015 | 18504072 | 0.48 | 0.16-1.50 |
| Immunoglobulin Human Normal | 3 | 3123 | 2015 | 18608851 | 0.40 | 0.13-1.23 |
| Interferon Beta-1A | 3 | 69550 | 2015 | 18542424 | 0.18 | 0.06-0.55 |
| Alemtuzumab | 3 | 155762 | 2015 | 18456212 | 2.46 | 0.79-7.63 |
| Belimumab | 3 | 189894 | 2015 | 18422080 | 1.19 | 0.38-3.68 |
| Natalizumab | 3 | 58771 | 2015 | 18553203 | 0.16 | 0.05-0.51 |
| Risankizumab | 3 | 167707 | 2015 | 18444267 | 0.54 | 0.17-1.67 |
| Thalidomide | 3 | 4715 | 2015 | 18607259 | 1.11 | 0.36-3.45 |
| Atorvastatin | 3 | 57168 | 2015 | 18554806 | 0.32 | 0.10-1.00 |
| Rosuvastatin | 3 | 51336 | 2015 | 18560638 | 0.62 | 0.20-1.93 |
| Diazepam | 3 | 44367 | 2015 | 18567607 | 1.59 | 0.51-4.95 |
| Ethinylestradiol; levonorgestrel | 3 | 24911 | 2015 | 18587063 | 7.22 | 2.33-22.43 |
| Levonorgestrel | 3 | 1867 | 2015 | 18610107 | 0.14 | 0.05-0.45 |
| Thiamazole | 3 | 9583 | 2015 | 18602391 | 14.84 | 4.78-46.10 |
| Pregabalin | 2 | 48865 | 2016 | 18563109 | 0.16 | 0.04-0.64 |
| Cefazolin | 2 | 10093 | 2016 | 18601881 | 9.25 | 2.31-37.04 |
| Cefuroxime | 2 | 631 | 2016 | 18611343 | 2.89 | 0.72-11.56 |
| Cilastatin; Imipenem | 2 | 35453 | 2016 | 18576521 | 4.45 | 1.11-17.79 |
| Daptomycin | 2 | 4944 | 2016 | 18607030 | 2.09 | 0.52-8.38 |
| Aciclovir | 2 | 68884 | 2016 | 18543090 | 1.83 | 0.46-7.32 |
| Beclometasone | 2 | 7164 | 2016 | 18604810 | 3.73 | 0.93-14.94 |
| Diclofenac; Misoprostol | 2 | 1996 | 2016 | 18609978 | 12.10 | 3.02-48.45 |
| Flurbiprofen | 2 | 6392 | 2016 | 18605582 | 67.39 | 16.76-271.00 |
| Ketorolac | 2 | 1938 | 2016 | 18610036 | 4.86 | 1.21-19.47 |
| Rifampicin | 2 | 20493 | 2016 | 18591481 | 2.81 | 0.70-11.25 |
| Bevacizumab | 2 | 4153 | 2016 | 18607821 | 0.27 | 0.07-1.07 |
| Brentuximab Vedotin | 2 | 2345 | 2016 | 18609629 | 2.58 | 0.64-10.31 |
| Cemiplimab | 2 | 3361 | 2016 | 18608613 | 9.53 | 2.38-38.15 |
| Cetuximab | 2 | 2604 | 2016 | 18609370 | 0.90 | 0.22-3.60 |
| Clofarabine | 2 | 66781 | 2016 | 18545193 | 7.87 | 1.97-31.52 |
| Colchicine | 2 | 27206 | 2016 | 18584768 | 5.49 | 1.37-21.99 |
| Fluorouracil | 2 | 8818 | 2016 | 18603156 | 0.94 | 0.23-3.75 |
| Gefitinib | 2 | 154664 | 2016 | 18457310 | 2.55 | 0.64-10.21 |
| Granulocyte Colony Stimulating Factor | 2 | 3691 | 2016 | 18608283 | 217.23 | 53.42-883.38 |
| Ivosidenib | 2 | 1526 | 2016 | 18610448 | 13.25 | 3.31-53.09 |
| Melphalan | 2 | 9547 | 2016 | 18602427 | 2.87 | 0.72-11.47 |
| Olaparib | 2 | 8782 | 2016 | 18603192 | 1.28 | 0.32-5.10 |
| Paclitaxel | 2 | 22208 | 2016 | 18589766 | 0.51 | 0.13-2.06 |
| Pemetrexed | 2 | 69436 | 2016 | 18542538 | 1.45 | 0.36-5.81 |
| Ponatinib | 2 | 82555 | 2016 | 18529419 | 2.03 | 0.51-8.13 |
| Quizartinib | 2 | 19686 | 2016 | 18592288 | 109.91 | 27.24-443.39 |
| Sunitinib | 2 | 274 | 2016 | 18611700 | 0.50 | 0.12-2.00 |
| Temsirolimus | 2 | 7238 | 2016 | 18604736 | 5.94 | 1.48-23.76 |
| Vorinostat | 2 | 85 | 2016 | 18611889 | 13.30 | 3.32-53.28 |
| Dabigatran | 2 | 311 | 2016 | 18611663 | 0.28 | 0.07-1.10 |
| Rivaroxaban | 2 | 1393 | 2016 | 18610581 | 0.15 | 0.04-0.60 |
| Lopinavir; Ritonavir | 2 | 3796 | 2016 | 18608178 | 2.93 | 0.73-11.71 |
| Telaprevir | 2 | 72873 | 2016 | 18539101 | 0.71 | 0.18-2.84 |
| Metoprolol | 2 | 6307 | 2016 | 18605667 | 0.70 | 0.17-2.78 |
| Teriparatide | 2 | 6438 | 2016 | 18605536 | 0.17 | 0.04-0.67 |
| Esomeprazole | 2 | 38892 | 2016 | 18573082 | 0.26 | 0.07-1.06 |
| Alendronic Acid | 2 | 26502 | 2016 | 18585472 | 0.52 | 0.13-2.08 |
| Denosumab | 2 | 47785 | 2016 | 18564189 | 0.12 | 0.03-0.47 |
| Risedronic Acid | 2 | 14467 | 2016 | 18597507 | 2.80 | 0.70-11.21 |
| Zoledronic Acid | 2 | 35819 | 2016 | 18576155 | 0.31 | 0.08-1.24 |
| Dapagliflozin | 2 | 12701 | 2016 | 18599273 | 0.68 | 0.17-2.71 |
| Leuprorelin | 2 | 499 | 2016 | 18611475 | 0.25 | 0.06-1.01 |
| Aldesleukin | 2 | 9089 | 2016 | 18602885 | 29.26 | 7.30-117.34 |
| Interferon Alfa | 2 | 115495 | 2016 | 18496479 | 59.37 | 14.77-238.61 |
| Plerixafor | 2 | 168 | 2016 | 18611806 | 37.00 | 9.22-148.47 |
| Fingolimod | 2 | 22833 | 2016 | 18589141 | 0.22 | 0.06-0.89 |
| Tofacitinib | 2 | 6569 | 2016 | 18605405 | 0.14 | 0.04-0.57 |
| Cyclobenzaprine | 2 | 6592 | 2016 | 18605382 | 7.09 | 1.77-28.38 |
| Ranibizumab | 2 | 121499 | 2016 | 18490475 | 0.81 | 0.20-3.23 |
| Tacrolimus | 2 | 36884 | 2016 | 18575090 | 0.31 | 0.08-1.23 |
| Varenicline | 2 | 59657 | 2016 | 18552317 | 0.23 | 0.06-0.92 |
| Desmopressin | 2 | 14327 | 2016 | 18597647 | 5.00 | 1.25-20.02 |
| Donepezil | 2 | 25966 | 2016 | 18586008 | 2.10 | 0.53-8.41 |
| Escitalopram | 2 | 3110 | 2016 | 18608864 | 0.83 | 0.21-3.32 |
| Methylphenidate | 2 | 109025 | 2016 | 18502949 | 0.47 | 0.12-1.90 |
| Olanzapine | 2 | 127849 | 2016 | 18484125 | 0.39 | 0.10-1.54 |
| Acetylsalicylic acid | 2 | 16961 | 2016 | 18595013 | 0.38 | 0.09-1.51 |
| Triamcinolone | 2 | 80213 | 2016 | 18531761 | 1.09 | 0.27-4.35 |
| Tamsulosin | 2 | 19774 | 2016 | 18592200 | 1.29 | 0.32-5.15 |
| Various | 2 | 1388 | 2016 | 18610586 | 0.93 | 0.23-3.73 |
| Diltiazem | 2 | 59398 | 2016 | 18552576 | 1.93 | 0.48-7.74 |
| Epoprostenol | 1 | 32533 | 2017 | 18579441 | 0.74 | 0.10-5.24 |
| Aliskiren | 1 | 2060 | 2017 | 18609914 | 1.65 | 0.23-11.72 |
| Olmesartan | 1 | 5593 | 2017 | 18606381 | 0.66 | 0.09-4.70 |
| Butalbital; Caffeine; Paracetamol | 1 | 7341 | 2017 | 18604633 | 10.86 | 1.53-77.19 |
| Erenumab | 1 | 5966 | 2017 | 18606008 | 0.20 | 0.03-1.41 |
| Lauromacrogol 400 | 1 | 4865 | 2017 | 18607109 | 7.32 | 1.03-52.05 |
| Dapsone | 1 | 1125 | 2017 | 18610849 | 6.14 | 0.86-43.63 |
| Cefotaxime | 1 | 126671 | 2017 | 18485303 | 8.88 | 1.25-63.14 |
| Dalfopristin; Quinupristin | 1 | 585 | 2017 | 18611389 | 161.89 | 22.41-1169.69 |
| Fosfomycin | 1 | 1646 | 2017 | 18610328 | 11.12 | 1.56-79.06 |
| Norfloxacin | 1 | 2094 | 2017 | 18609880 | 27.14 | 3.81-193.32 |
| Sulfamethoxazole | 1 | 83 | 2017 | 18611891 | 67.35 | 9.42-481.83 |
| Amikacin | 1 | 12474 | 2017 | 18599500 | 1.55 | 0.22-10.98 |
| Alpha-1-Antitrypsin | 1 | 753 | 2017 | 18611221 | 1.26 | 0.18-8.93 |
| Bismuth; Metronidazole; Tetracycline | 1 | 2959 | 2017 | 18609015 | 9.70 | 1.36-68.99 |
| Loperamide | 1 | 951 | 2017 | 18611023 | 0.69 | 0.10-4.90 |
| Gabapentin Enacarbil | 1 | 6643 | 2017 | 18605331 | 5.16 | 0.73-36.67 |
| Topiramate | 1 | 2849 | 2017 | 18609125 | 0.41 | 0.06-2.90 |
| Febuxostat | 1 | 40779 | 2017 | 18571195 | 1.91 | 0.27-13.59 |
| Pegloticase | 1 | 850 | 2017 | 18611124 | 3.27 | 0.46-23.24 |
| Eltrombopag | 1 | 3002 | 2017 | 18608972 | 0.43 | 0.06-3.05 |
| Fluticasone; Salmeterol | 1 | 23211 | 2017 | 18588763 | 0.20 | 0.03-1.44 |
| Ibuprofen; Pseudoephedrine | 1 | 13581 | 2017 | 18598393 | 8.05 | 1.13-57.24 |
| Rofecoxib | 1 | 1026 | 2017 | 18610948 | 0.26 | 0.04-1.83 |
| Valdecoxib | 1 | 1039 | 2017 | 18610935 | 1.01 | 0.14-7.17 |
| Isoniazid | 1 | 1034 | 2017 | 18610940 | 3.44 | 0.48-24.45 |
| Isoniazid; Rifampicin | 1 | 18569 | 2017 | 18593405 | 22.95 | 3.22-163.43 |
| Isavuconazole | 1 | 25020 | 2017 | 18586954 | 11.23 | 1.58-79.83 |
| Isavuconazonium | 1 | 880 | 2017 | 18611094 | 6.63 | 0.93-47.15 |
| Posaconazole | 1 | 2802 | 2017 | 18609172 | 2.57 | 0.36-18.24 |
| Voriconazole | 1 | 741 | 2017 | 18611233 | 0.75 | 0.11-5.33 |
| Belantamab Mafodotin | 1 | 57 | 2017 | 18611917 | 4.41 | 0.62-31.31 |
| Bexarotene | 1 | 1503 | 2017 | 18610471 | 12.25 | 1.72-87.15 |
| Binimetinib | 1 | 5 | 2017 | 18611969 | 3.12 | 0.44-22.15 |
| Bosutinib | 1 | 11284 | 2017 | 18600690 | 1.39 | 0.20-9.86 |
| Brigatinib | 1 | 5268 | 2017 | 18606706 | 3.24 | 0.46-23.01 |
| Carfilzomib | 1 | 5573 | 2017 | 18606401 | 0.68 | 0.10-4.82 |
| Cedazuridine; Decitabine | 1 | 11795 | 2017 | 18600179 | 8.99 | 1.26-63.94 |
| Chlorambucil | 1 | 7419 | 2017 | 18604555 | 8.92 | 1.26-63.44 |
| Cisplatin | 1 | 49653 | 2017 | 18562321 | 0.50 | 0.07-3.53 |
| Dacarbazine | 1 | 21462 | 2017 | 18590512 | 12.45 | 1.75-88.56 |
| Durvalumab | 1 | 2116 | 2017 | 18609858 | 0.78 | 0.11-5.55 |
| Encorafenib | 1 | 22086 | 2017 | 18589888 | 2.02 | 0.29-14.39 |
| Gemtuzumab Ozogamicin | 1 | 4556 | 2017 | 18607418 | 4.40 | 0.62-31.25 |
| Granulocyte Macrophage Colony Stimulating Factor | 1 | 1272 | 2017 | 18610702 | 10.11 | 1.42-71.86 |
| Idelalisib | 1 | 17837 | 2017 | 18594137 | 1.61 | 0.23-11.47 |
| Lenvatinib | 1 | 46428 | 2017 | 18565546 | 0.42 | 0.06-2.96 |
| Lorlatinib | 1 | 11859 | 2017 | 18600115 | 2.37 | 0.33-16.84 |
| Niraparib | 1 | 12492 | 2017 | 18599482 | 0.45 | 0.06-3.23 |
| Obinutuzumab | 1 | 46305 | 2017 | 18565669 | 1.19 | 0.17-8.45 |
| Ofatumumab | 1 | 53489 | 2017 | 18558485 | 0.35 | 0.05-2.47 |
| Osimertinib | 1 | 3716 | 2017 | 18608258 | 0.40 | 0.06-2.87 |
| Oxaliplatin | 1 | 4821 | 2017 | 18607153 | 0.29 | 0.04-2.07 |
| Palbociclib | 1 | 5881 | 2017 | 18606093 | 0.12 | 0.02-0.85 |
| Panitumumab | 1 | 12940 | 2017 | 18599034 | 0.93 | 0.13-6.60 |
| Pazopanib | 1 | 45416 | 2017 | 18566558 | 0.38 | 0.05-2.71 |
| Trastuzumab Emtansine | 1 | 830 | 2017 | 18611144 | 1.91 | 0.27-13.54 |
| Vincristine | 1 | 1788 | 2017 | 18610186 | 1.23 | 0.17-8.73 |
| Diphenhydramine | 1 | 2098 | 2017 | 18609876 | 0.82 | 0.12-5.81 |
| Promethazine | 1 | 5182 | 2017 | 18606792 | 2.62 | 0.37-18.60 |
| Enoxaparin | 1 | 913 | 2017 | 18611061 | 0.52 | 0.07-3.67 |
| Ticagrelor | 1 | 8539 | 2017 | 18603435 | 0.46 | 0.06-3.26 |
| Cobicistat; Elvitegravir; Emtricitabine; Tenofovir | 1 | 919 | 2017 | 18611055 | 10.49 | 1.47-74.56 |
| Dolutegravir | 1 | 1146 | 2017 | 18610828 | 1.75 | 0.25-12.44 |
| Emtricitabine; Tenofovir Disoproxil | 1 | 5715 | 2017 | 18606259 | 0.42 | 0.06-2.96 |
| Enfuvirtide | 1 | 405 | 2017 | 18611569 | 7.25 | 1.02-51.56 |
| Ledipasvir; Sofosbuvir | 1 | 29550 | 2017 | 18582424 | 0.42 | 0.06-2.97 |
| Oseltamivir | 1 | 77000 | 2017 | 18534974 | 0.61 | 0.09-4.37 |
| Ribavirin | 1 | 3095 | 2017 | 18608879 | 0.50 | 0.07-3.57 |
| Ritonavir | 1 | 42293 | 2017 | 18569681 | 1.38 | 0.19-9.80 |
| Zanamivir | 1 | 3242 | 2017 | 18608732 | 4.03 | 0.57-28.62 |
| Zidovudine | 1 | 6981 | 2017 | 18604993 | 3.15 | 0.44-22.37 |
| Calcium | 1 | 822 | 2017 | 18611152 | 3.07 | 0.43-21.84 |
| Nicardipine | 1 | 1391 | 2017 | 18610583 | 10.86 | 1.53-77.19 |
| Paricalcitol | 1 | 2681 | 2017 | 18609293 | 0.91 | 0.13-6.43 |
| Dronedarone | 1 | 402 | 2017 | 18611572 | 1.66 | 0.23-11.76 |
| Ivabradine | 1 | 2908 | 2017 | 18609066 | 3.17 | 0.45-22.54 |
| Iopamidol | 1 | 8170 | 2017 | 18603804 | 2.85 | 0.40-20.22 |
| Iopromide | 1 | 1260 | 2017 | 18610714 | 1.32 | 0.19-9.39 |
| Codeine | 1 | 22012 | 2017 | 18589962 | 3.29 | 0.46-23.40 |
| Bendroflumethiazide | 1 | 22141 | 2017 | 18589833 | 111.17 | 15.47-799.00 |
| Pantoprazole | 1 | 51480 | 2017 | 18560494 | 0.30 | 0.04-2.10 |
| Benralizumab | 1 | 32496 | 2017 | 18579478 | 0.74 | 0.10-5.25 |
| Montelukast | 1 | 13380 | 2017 | 18598594 | 0.42 | 0.06-3.01 |
| Canagliflozin | 1 | 3893 | 2017 | 18608081 | 0.40 | 0.06-2.82 |
| Empagliflozin; metformin | 1 | 14 | 2017 | 18611960 | 4.36 | 0.61-30.98 |
| Insulin aspart; insulin degludec | 1 | 21721 | 2017 | 18590253 | 22.78 | 3.20-162.22 |
| Insulin human | 1 | 850 | 2017 | 18611124 | 0.31 | 0.04-2.21 |
| Insulin lispro | 1 | 20272 | 2017 | 18591702 | 0.12 | 0.02-0.85 |
| Liraglutide | 1 | 340 | 2017 | 18611634 | 0.28 | 0.04-2.01 |
| Abiraterone | 1 | 7755 | 2017 | 18604219 | 0.28 | 0.04-2.01 |
| Enzalutamide | 1 | 26545 | 2017 | 18585429 | 0.20 | 0.03-1.41 |
| Goserelin | 1 | 13925 | 2017 | 18598049 | 1.78 | 0.25-12.65 |
| Interferon Alfa-2B | 1 | 15004 | 2017 | 18596970 | 2.98 | 0.42-21.18 |
| Anifrolumab | 1 | 22812 | 2017 | 18589162 | 8.20 | 1.15-58.31 |
| Apremilast | 1 | 31684 | 2017 | 18580290 | 0.07 | 0.01-0.51 |
| Basiliximab | 1 | 76597 | 2017 | 18535377 | 5.61 | 0.79-39.84 |
| Eculizumab | 1 | 9923 | 2017 | 18602051 | 0.19 | 0.03-1.32 |
| Sarilumab | 1 | 31215 | 2017 | 18580759 | 0.66 | 0.09-4.67 |
| Atorvastatin; ezetimibe | 1 | 10189 | 2017 | 18601785 | 15.77 | 2.22-112.22 |
| Ezetimibe; simvastatin | 1 | 24145 | 2017 | 18587829 | 2.48 | 0.35-17.64 |
| Fenofibrate | 1 | 2821 | 2017 | 18609153 | 1.57 | 0.22-11.14 |
| Simvastatin | 1 | 238 | 2017 | 18611736 | 0.33 | 0.05-2.33 |
| Tizanidine | 1 | 1847 | 2017 | 18610127 | 4.37 | 0.61-31.04 |
| Finasteride | 1 | 3593 | 2017 | 18608381 | 0.71 | 0.10-5.06 |
| Mequinol; tretinoin | 1 | 3524 | 2017 | 18608450 | 659.11 | 86.63-5014.82 |
| Pimecrolimus | 1 | 18321 | 2017 | 18593653 | 5.00 | 0.70-35.50 |
| Intrauterine contraceptive device | 1 | 6685 | 2017 | 18605289 | 0.22 | 0.03-1.55 |
| Tetrabenazine | 1 | 35827 | 2017 | 18576147 | 1.29 | 0.18-9.15 |
| Lanreotide | 1 | 14020 | 2017 | 18597954 | 1.13 | 0.16-8.02 |
| Amitriptyline | 1 | 51813 | 2017 | 18560161 | 1.90 | 0.27-13.47 |
| Bupropion | 1 | 28062 | 2017 | 18583912 | 0.23 | 0.03-1.60 |
| Citalopram | 1 | 137 | 2017 | 18611837 | 0.37 | 0.05-2.62 |
| Sertraline | 1 | 38132 | 2017 | 18573842 | 0.18 | 0.03-1.26 |
| Perphenazine | 1 | 7165 | 2017 | 18604809 | 38.77 | 5.44-276.51 |
| Zolpidem | 1 | 20060 | 2017 | 18591914 | 0.42 | 0.06-2.99 |
| Etonogestrel | 1 | 2112 | 2017 | 18609862 | 0.17 | 0.02-1.22 |
| Ulipristal | 1 | 22581 | 2017 | 18589393 | 12.82 | 1.80-91.15 |
| Diclofenac; linum usitatissimum; menthol; salicylic acid | 1 | 4841 | 2017 | 18607133 | 1845.51 | 215.51-15804.14 |
| Epinephrine | 1 | 720 | 2017 | 18611254 | 0.78 | 0.11-5.52 |
| Hydrocortisone | 1 | 9137 | 2017 | 18602837 | 1.08 | 0.15-7.67 |
| Ibritumomab tiuxetan | 1 | 7507 | 2017 | 18604467 | 10.04 | 1.41-71.39 |
| Levothyroxine | 1 | 12283 | 2017 | 18599691 | 0.18 | 0.03-1.27 |
| Alfuzosin | 1 | 2291 | 2017 | 18609683 | 4.48 | 0.63-31.83 |
| Dutasteride | 1 | 2930 | 2017 | 18609044 | 1.24 | 0.18-8.83 |
| Testosterone | 1 | 21929 | 2017 | 18590045 | 0.24 | 0.03-1.72 |

Blue bars indicate drugs with a positive signal, defined as ≥3 cases and a lower bound of the 95% confidence interval for the ROR > 1. FAERS, U.S. FOOD AND DRUG ADMINISTRATION Adverse Event Reporting System; ROR, reporting odds ratio; CI, confidence interval.

**Supplementary Table 4.** Disproportionality analysis of drugs reported in association with drug-induced Sweet’s syndrome in medical doctor–submitted cases from the FAERS database.

| **Drug** | **a** | **b** | **c** | **d** | **ROR** | **95%CI** |
| --- | --- | --- | --- | --- | --- | --- |
| Sulfamethoxazole;Trimethoprim | 52 | 2433 | 780 | 4156450 | 113.89 | 85.77-151.22 |
| Infliximab | 34 | 51819 | 798 | 4107064 | 3.38 | 2.40-4.76 |
| Adalimumab | 33 | 70974 | 799 | 4087909 | 2.38 | 1.68-3.37 |
| Azacitidine | 27 | 6148 | 805 | 4152735 | 22.66 | 15.43-33.27 |
| Bortezomib | 18 | 17635 | 814 | 4141248 | 5.19 | 3.25-8.29 |
| Lenalidomide | 18 | 66756 | 814 | 4092127 | 1.36 | 0.85-2.16 |
| Pegfilgrastim | 18 | 36425 | 814 | 4122458 | 2.5 | 1.57-3.99 |
| Azathioprine | 16 | 1444 | 816 | 4157439 | 56.45 | 34.33-92.84 |
| Hydralazine | 16 | 639 | 816 | 4158244 | 127.6 | 77.33-210.54 |
| Levofloxacin | 16 | 7514 | 816 | 4151369 | 10.83 | 6.60-17.78 |
| Mesalazine | 16 | 3307 | 816 | 4155576 | 24.64 | 15.01-40.46 |
| Minocycline | 15 | 1433 | 817 | 4157450 | 53.27 | 31.88-89.00 |
| Gabapentin | 14 | 15970 | 818 | 4142913 | 4.44 | 2.62-7.53 |
| Ciprofloxacin | 13 | 6581 | 819 | 4152302 | 10.02 | 5.79-17.33 |
| Filgrastim | 13 | 3789 | 819 | 4155094 | 17.41 | 10.05-30.13 |
| Clindamycin | 12 | 4666 | 820 | 4154217 | 13.03 | 7.36-23.05 |
| Docetaxel | 10 | 8971 | 822 | 4149912 | 5.63 | 3.02-10.50 |
| Etanercept | 10 | 223315 | 822 | 3935568 | 0.21 | 0.11-0.40 |
| Ibrutinib | 10 | 11161 | 822 | 4147722 | 4.52 | 2.42-8.44 |
| Carboplatin | 9 | 15354 | 823 | 4143529 | 2.95 | 1.53-5.69 |
| Furosemide | 9 | 7049 | 823 | 4151834 | 6.44 | 3.34-12.43 |
| Gilteritinib | 9 | 1361 | 823 | 4157522 | 33.41 | 17.28-64.57 |
| Venetoclax | 9 | 13853 | 823 | 4145030 | 3.27 | 1.70-6.31 |
| Amoxicillin | 8 | 6580 | 824 | 4152303 | 6.13 | 3.05-12.30 |
| Capecitabine | 8 | 14242 | 824 | 4144641 | 2.83 | 1.41-5.67 |
| Celecoxib | 8 | 7341 | 824 | 4151542 | 5.49 | 2.74-11.02 |
| Dupilumab | 8 | 179340 | 824 | 3979543 | 0.22 | 0.11-0.43 |
| Ibuprofen | 8 | 17131 | 824 | 4141752 | 2.35 | 1.17-4.71 |
| Azithromycin | 7 | 6968 | 825 | 4151915 | 5.06 | 2.40-10.64 |
| Decitabine | 7 | 935 | 825 | 4157948 | 37.73 | 17.88-79.62 |
| Methotrexate | 7 | 45099 | 825 | 4113784 | 0.77 | 0.37-1.63 |
| Paracetamol | 7 | 17768 | 825 | 4141115 | 1.98 | 0.94-4.16 |
| Anastrozole | 6 | 3370 | 826 | 4155513 | 8.96 | 4.01-20.01 |
| Codeine;Paracetamol | 6 | 549 | 826 | 4158334 | 55.02 | 24.54-123.36 |
| Cytarabine | 6 | 3800 | 826 | 4155083 | 7.94 | 3.56-17.74 |
| Ketoconazole | 6 | 524 | 826 | 4158359 | 57.65 | 25.70-129.27 |
| Ruxolitinib | 6 | 13924 | 826 | 4144959 | 2.16 | 0.97-4.83 |
| Amlodipine | 5 | 13995 | 827 | 4144888 | 1.79 | 0.74-4.31 |
| Bendamustine | 5 | 5022 | 827 | 4153861 | 5 | 2.08-12.05 |
| Dexamethasone | 5 | 16269 | 827 | 4142614 | 1.54 | 0.64-3.71 |
| Diclofenac | 5 | 7014 | 827 | 4151869 | 3.58 | 1.49-8.62 |
| Gemcitabine | 5 | 10322 | 827 | 4148561 | 2.43 | 1.01-5.85 |
| Ixazomib | 5 | 6260 | 827 | 4152623 | 4.01 | 1.66-9.66 |
| Lamotrigine | 5 | 11459 | 827 | 4147424 | 2.19 | 0.91-5.27 |
| Letrozole | 5 | 7668 | 827 | 4151215 | 3.27 | 1.36-7.89 |
| Tocilizumab | 5 | 17861 | 827 | 4141022 | 1.4 | 0.58-3.38 |
| Vancomycin | 5 | 5459 | 827 | 4153424 | 4.6 | 1.91-11.09 |
| Amoxicillin;Clavulanic Acid | 4 | 3779 | 828 | 4155104 | 5.31 | 1.99-14.19 |
| Darbepoetin Alfa | 4 | 5699 | 828 | 4153184 | 3.52 | 1.32-9.41 |
| Etoposide | 4 | 3437 | 828 | 4155446 | 5.84 | 2.19-15.61 |
| Imatinib | 4 | 15518 | 828 | 4143365 | 1.29 | 0.48-3.45 |
| Ipilimumab | 4 | 6012 | 828 | 4152871 | 3.34 | 1.25-8.92 |
| Mitoxantrone | 4 | 589 | 828 | 4158294 | 34.11 | 12.73-91.39 |
| Ofloxacin | 4 | 418 | 828 | 4158465 | 48.06 | 17.91-128.96 |
| Peginterferon Alfa-2A | 4 | 7413 | 828 | 4151470 | 2.71 | 1.01-7.23 |
| Prednisolone | 4 | 9572 | 828 | 4149311 | 2.09 | 0.78-5.59 |
| Prednisone | 4 | 8840 | 828 | 4150043 | 2.27 | 0.85-6.06 |
| Sulfasalazine | 4 | 2987 | 828 | 4155896 | 6.72 | 2.52-17.96 |
| Trastuzumab | 4 | 15085 | 828 | 4143798 | 1.33 | 0.50-3.54 |
| Abatacept | 3 | 8553 | 829 | 4150330 | 1.76 | 0.57-5.46 |
| Aripiprazole | 3 | 15127 | 829 | 4143756 | 0.99 | 0.32-3.08 |
| Bisoprolol | 3 | 3528 | 829 | 4155355 | 4.26 | 1.37-13.25 |
| Ceftriaxone | 3 | 3326 | 829 | 4155557 | 4.52 | 1.45-14.06 |
| Dabrafenib | 3 | 4403 | 829 | 4154480 | 3.41 | 1.10-10.61 |
| Dasatinib | 3 | 4171 | 829 | 4154712 | 3.6 | 1.16-11.20 |
| Efalizumab | 3 | 1008 | 829 | 4157875 | 14.93 | 4.80-46.46 |
| Everolimus | 3 | 11131 | 829 | 4147752 | 1.35 | 0.43-4.19 |
| Golimumab | 3 | 8307 | 829 | 4150576 | 1.81 | 0.58-5.62 |
| Hydroxychloroquine | 3 | 3720 | 829 | 4155163 | 4.04 | 1.30-12.56 |
| Interferon Beta-1B | 3 | 2930 | 829 | 4155953 | 5.13 | 1.65-15.96 |
| Isotretinoin | 3 | 17806 | 829 | 4141077 | 0.84 | 0.27-2.62 |
| Leflunomide | 3 | 5404 | 829 | 4153479 | 2.78 | 0.89-8.64 |
| Linezolid | 3 | 4449 | 829 | 4154434 | 3.38 | 1.09-10.50 |
| Mercaptopurine | 3 | 835 | 829 | 4158048 | 18.02 | 5.79-56.10 |
| Mycophenolic Acid | 3 | 17511 | 829 | 4141372 | 0.86 | 0.28-2.66 |
| Nivolumab | 3 | 19790 | 829 | 4139093 | 0.76 | 0.24-2.35 |
| Omalizumab | 3 | 19853 | 829 | 4139030 | 0.75 | 0.24-2.34 |
| Secukinumab | 3 | 17882 | 829 | 4141001 | 0.84 | 0.27-2.60 |
| Stavudine | 3 | 134 | 829 | 4158749 | 112.31 | 35.70-353.37 |
| Tretinoin | 3 | 598 | 829 | 4158285 | 25.16 | 8.08-78.41 |
| Vedolizumab | 3 | 17038 | 829 | 4141845 | 0.88 | 0.28-2.73 |
| Vemurafenib | 3 | 4380 | 829 | 4154503 | 3.43 | 1.10-10.67 |
| Alendronic Acid | 2 | 8790 | 830 | 4150093 | 1.14 | 0.28-4.56 |
| Atorvastatin | 2 | 18352 | 830 | 4140531 | 0.54 | 0.14-2.18 |
| Bevacizumab | 2 | 34667 | 830 | 4124216 | 0.29 | 0.07-1.15 |
| Cemiplimab | 2 | 1140 | 830 | 4157743 | 8.79 | 2.19-35.24 |
| Cilastatin;Imipenem | 2 | 898 | 830 | 4157985 | 11.16 | 2.78-44.76 |
| Clarithromycin | 2 | 4343 | 830 | 4154540 | 2.31 | 0.58-9.24 |
| Clozapine | 2 | 39240 | 830 | 4119643 | 0.25 | 0.06-1.01 |
| Denosumab | 2 | 65896 | 830 | 4092987 | 0.15 | 0.04-0.60 |
| Doxorubicin | 2 | 13498 | 830 | 4145385 | 0.74 | 0.18-2.96 |
| Enasidenib | 2 | 775 | 830 | 4158108 | 12.93 | 3.22-51.87 |
| Esomeprazole | 2 | 6534 | 830 | 4152349 | 1.53 | 0.38-6.13 |
| Fluorouracil | 2 | 6517 | 830 | 4152366 | 1.54 | 0.38-6.15 |
| Fumaric Acid | 2 | 11901 | 830 | 4146982 | 0.84 | 0.21-3.36 |
| Glatiramer | 2 | 5824 | 830 | 4153059 | 1.72 | 0.43-6.88 |
| Hydroxycarbamide | 2 | 990 | 830 | 4157893 | 10.12 | 2.52-40.59 |
| Idarubicin | 2 | 418 | 830 | 4158465 | 23.97 | 5.97-96.33 |
| Interferon Alfa | 2 | 158 | 830 | 4158725 | 63.42 | 15.70-256.26 |
| Ivosidenib | 2 | 360 | 830 | 4158523 | 27.83 | 6.92-111.91 |
| Lamivudine | 2 | 2850 | 830 | 4156033 | 3.51 | 0.88-14.08 |
| Leuprorelin | 2 | 10220 | 830 | 4148663 | 0.98 | 0.24-3.92 |
| Levetiracetam | 2 | 19852 | 830 | 4139031 | 0.5 | 0.13-2.01 |
| Lopinavir;Ritonavir | 2 | 2518 | 830 | 4156365 | 3.98 | 0.99-15.94 |
| Naproxen | 2 | 3472 | 830 | 4155411 | 2.88 | 0.72-11.56 |
| Plerixafor | 2 | 204 | 830 | 4158679 | 49.12 | 12.18-198.08 |
| Pregabalin | 2 | 26332 | 830 | 4132551 | 0.38 | 0.09-1.51 |
| Quizartinib | 2 | 44 | 830 | 4158839 | 227.76 | 55.12-941.04 |
| Ramipril | 2 | 5614 | 830 | 4153269 | 1.78 | 0.44-7.14 |
| Ranibizumab | 2 | 9807 | 830 | 4149076 | 1.02 | 0.25-4.08 |
| Rituximab | 2 | 41380 | 830 | 4117503 | 0.24 | 0.06-0.96 |
| Rivaroxaban | 2 | 36398 | 830 | 4122485 | 0.27 | 0.07-1.09 |
| Sorafenib | 2 | 7681 | 830 | 4151202 | 1.3 | 0.33-5.22 |
| Thalidomide | 2 | 10975 | 830 | 4147908 | 0.91 | 0.23-3.65 |
| Thiamazole | 2 | 443 | 830 | 4158440 | 22.62 | 5.63-90.88 |
| Tofacitinib | 2 | 17657 | 830 | 4141226 | 0.57 | 0.14-2.26 |
| Trametinib | 2 | 2470 | 830 | 4156413 | 4.05 | 1.01-16.25 |
| Upadacitinib | 2 | 6175 | 830 | 4152708 | 1.62 | 0.40-6.49 |
| Valaciclovir | 2 | 3700 | 830 | 4155183 | 2.71 | 0.68-10.84 |
| Abiraterone | 1 | 5800 | 831 | 4153083 | 0.86 | 0.12-6.13 |
| Acetylsalicylic Acid | 1 | 6974 | 831 | 4151909 | 0.72 | 0.10-5.09 |
| Aciclovir | 1 | 3059 | 831 | 4155824 | 1.63 | 0.23-11.62 |
| Alemtuzumab | 1 | 4198 | 831 | 4154685 | 1.19 | 0.17-8.47 |
| Alfuzosin | 1 | 476 | 831 | 4158407 | 10.51 | 1.48-74.88 |
| Allopurinol | 1 | 3340 | 831 | 4155543 | 1.5 | 0.21-10.64 |
| Anifrolumab | 1 | 556 | 831 | 4158327 | 9 | 1.26-64.08 |
| Atorvastatin;Ezetimibe | 1 | 259 | 831 | 4158624 | 19.32 | 2.71-137.85 |
| Belantamab Mafodotin | 1 | 1188 | 831 | 4157695 | 4.21 | 0.59-29.96 |
| Benralizumab | 1 | 3979 | 831 | 4154904 | 1.26 | 0.18-8.93 |
| Bexarotene | 1 | 178 | 831 | 4158705 | 28.11 | 3.93-200.93 |
| Bosutinib | 1 | 1762 | 831 | 4157121 | 2.84 | 0.40-20.19 |
| Budesonide | 1 | 3225 | 831 | 4155658 | 1.55 | 0.22-11.02 |
| Bupropion | 1 | 6020 | 831 | 4152863 | 0.83 | 0.12-5.90 |
| Butalbital;Caffeine;Paracetamol | 1 | 299 | 831 | 4158584 | 16.74 | 2.35-119.35 |
| Canagliflozin | 1 | 6912 | 831 | 4151971 | 0.72 | 0.10-5.14 |
| Carfilzomib | 1 | 7248 | 831 | 4151635 | 0.69 | 0.10-4.90 |
| Cefazolin | 1 | 524 | 831 | 4158359 | 9.55 | 1.34-68.00 |
| Cefuroxime | 1 | 1758 | 831 | 4157125 | 2.85 | 0.40-20.24 |
| Certolizumab Pegol | 1 | 12307 | 831 | 4146576 | 0.41 | 0.06-2.88 |
| Cetuximab | 1 | 2285 | 831 | 4156598 | 2.19 | 0.31-15.57 |
| Ciclosporin | 1 | 8388 | 831 | 4150495 | 0.6 | 0.08-4.23 |
| Cobicistat;Elvitegravir;Emtricitabine;Tenofovir | 1 | 243 | 831 | 4158640 | 20.59 | 2.89-146.97 |
| Codeine | 1 | 581 | 831 | 4158302 | 8.61 | 1.21-61.32 |
| Cyclobenzaprine | 1 | 721 | 831 | 4158162 | 6.94 | 0.98-49.40 |
| Cytarabine;Daunorubicin | 1 | 592 | 831 | 4158291 | 8.45 | 1.19-60.18 |
| Dapagliflozin | 1 | 9951 | 831 | 4148932 | 0.5 | 0.07-3.57 |
| Daptomycin | 1 | 3119 | 831 | 4155764 | 1.6 | 0.23-11.40 |
| Daunorubicin | 1 | 402 | 831 | 4158481 | 12.45 | 1.75-88.69 |
| Deferasirox | 1 | 5668 | 831 | 4153215 | 0.88 | 0.12-6.27 |
| Desmopressin | 1 | 559 | 831 | 4158324 | 8.95 | 1.26-63.74 |
| Diltiazem | 1 | 2662 | 831 | 4156221 | 1.88 | 0.26-13.36 |
| Diphenhydramine | 1 | 2482 | 831 | 4156401 | 2.02 | 0.28-14.33 |
| Doxycycline | 1 | 4385 | 831 | 4154498 | 1.14 | 0.16-8.11 |
| Durvalumab | 1 | 6938 | 831 | 4151945 | 0.72 | 0.10-5.12 |
| Eltrombopag | 1 | 4604 | 831 | 4154279 | 1.09 | 0.15-7.72 |
| Empagliflozin;Metformin | 1 | 862 | 831 | 4158021 | 5.8 | 0.82-41.30 |
| Enfuvirtide | 1 | 532 | 831 | 4158351 | 9.41 | 1.32-66.98 |
| Enoxaparin | 1 | 3758 | 831 | 4155125 | 1.33 | 0.19-9.46 |
| Finasteride | 1 | 2524 | 831 | 4156359 | 1.98 | 0.28-14.09 |
| Fingolimod | 1 | 18241 | 831 | 4140642 | 0.27 | 0.04-1.94 |
| Gabapentin Enacarbil | 1 | 631 | 831 | 4158252 | 7.93 | 1.11-56.45 |
| Gefitinib | 1 | 2674 | 831 | 4156209 | 1.87 | 0.26-13.30 |
| Gemtuzumab Ozogamicin | 1 | 1050 | 831 | 4157833 | 4.77 | 0.67-33.90 |
| Granulocyte Colony Stimulating Factor | 1 | 1 | 831 | 4158882 | 5004.67 | 312.76-80083.06 |
| Granulocyte Macrophage Colony Stimulating Factor | 1 | 212 | 831 | 4158671 | 23.61 | 3.31-168.56 |
| Ibuprofen;Pseudoephedrine | 1 | 42 | 831 | 4158841 | 119.16 | 16.38-866.80 |
| Insulin Aspart;Insulin Degludec | 1 | 75 | 831 | 4158808 | 66.73 | 9.27-480.49 |
| Interferon Alfa-2B | 1 | 1344 | 831 | 4157539 | 3.72 | 0.52-26.48 |
| Interferon Beta-1A | 1 | 25179 | 831 | 4133704 | 0.2 | 0.03-1.40 |
| Intrauterine Contraceptive Device | 1 | 8284 | 831 | 4150599 | 0.6 | 0.08-4.29 |
| Iopromide | 1 | 1027 | 831 | 4157856 | 4.87 | 0.68-34.66 |
| Isavuconazole | 1 | 187 | 831 | 4158696 | 26.76 | 3.75-191.21 |
| Ivabradine | 1 | 1395 | 831 | 4157488 | 3.59 | 0.50-25.51 |
| Lanreotide | 1 | 1272 | 831 | 4157611 | 3.93 | 0.55-27.98 |
| Lauromacrogol 400 | 1 | 320 | 831 | 4158563 | 15.64 | 2.19-111.49 |
| Ledipasvir;Sofosbuvir | 1 | 4112 | 831 | 4154771 | 1.22 | 0.17-8.64 |
| Lenvatinib | 1 | 13647 | 831 | 4145236 | 0.37 | 0.05-2.60 |
| Levonorgestrel | 1 | 42006 | 831 | 4116877 | 0.12 | 0.02-0.84 |
| Liraglutide | 1 | 7687 | 831 | 4151196 | 0.65 | 0.09-4.62 |
| Metformin | 1 | 18270 | 831 | 4140613 | 0.27 | 0.04-1.94 |
| Methylprednisolone | 1 | 6983 | 831 | 4151900 | 0.72 | 0.10-5.09 |
| Montelukast | 1 | 4981 | 831 | 4153902 | 1 | 0.14-7.13 |
| Nilotinib | 1 | 8771 | 831 | 4150112 | 0.57 | 0.08-4.05 |
| Nitrofurantoin | 1 | 1091 | 831 | 4157792 | 4.59 | 0.64-32.63 |
| Norfloxacin | 1 | 62 | 831 | 4158821 | 80.72 | 11.18-582.83 |
| Ofatumumab | 1 | 3530 | 831 | 4155353 | 1.42 | 0.20-10.07 |
| Olaparib | 1 | 4891 | 831 | 4153992 | 1.02 | 0.14-7.27 |
| Omeprazole | 1 | 6056 | 831 | 4152827 | 0.83 | 0.12-5.87 |
| Osimertinib | 1 | 6004 | 831 | 4152879 | 0.83 | 0.12-5.92 |
| Oxaliplatin | 1 | 11120 | 831 | 4147763 | 0.45 | 0.06-3.19 |
| Pegloticase | 1 | 826 | 831 | 4158057 | 6.06 | 0.85-43.11 |
| Pembrolizumab | 1 | 20411 | 831 | 4138472 | 0.24 | 0.03-1.73 |
| Perphenazine | 1 | 40 | 831 | 4158843 | 125.12 | 17.18-911.19 |
| Pomalidomide | 1 | 12664 | 831 | 4146219 | 0.39 | 0.06-2.80 |
| Posaconazole | 1 | 1123 | 831 | 4157760 | 4.46 | 0.63-31.69 |
| Propylthiouracil | 1 | 186 | 831 | 4158697 | 26.91 | 3.77-192.25 |
| Ribavirin | 1 | 5767 | 831 | 4153116 | 0.87 | 0.12-6.16 |
| Rifampicin | 1 | 1796 | 831 | 4157087 | 2.79 | 0.39-19.81 |
| Risankizumab | 1 | 3137 | 831 | 4155746 | 1.59 | 0.22-11.33 |
| Ritonavir | 1 | 1972 | 831 | 4156911 | 2.54 | 0.36-18.04 |
| Rofecoxib | 1 | 11705 | 831 | 4147178 | 0.43 | 0.06-3.03 |
| Rosuvastatin | 1 | 11848 | 831 | 4147035 | 0.42 | 0.06-2.99 |
| Sarilumab | 1 | 7509 | 831 | 4151374 | 0.67 | 0.09-4.73 |
| Sulfamethoxazole | 1 | 12 | 831 | 4158871 | 417.05 | 54.17-3211.15 |
| Sunitinib | 1 | 11183 | 831 | 4147700 | 0.45 | 0.06-3.17 |
| Temsirolimus | 1 | 1780 | 831 | 4157103 | 2.81 | 0.40-19.99 |
| Terbinafine | 1 | 2391 | 831 | 4156492 | 2.09 | 0.29-14.87 |
| Topiramate | 1 | 5965 | 831 | 4152918 | 0.84 | 0.12-5.96 |
| Topotecan | 1 | 1198 | 831 | 4157685 | 4.18 | 0.59-29.71 |
| Trastuzumab Emtansine | 1 | 2573 | 831 | 4156310 | 1.94 | 0.27-13.82 |
| Ustekinumab | 1 | 13529 | 831 | 4145354 | 0.37 | 0.05-2.62 |
| Valdecoxib | 1 | 927 | 831 | 4157956 | 5.4 | 0.76-38.40 |
| Various | 1 | 4631 | 831 | 4154252 | 1.08 | 0.15-7.67 |
| Vorinostat | 1 | 351 | 831 | 4158532 | 14.26 | 2.00-101.62 |
| Zanamivir | 1 | 975 | 831 | 4157908 | 5.13 | 0.72-36.51 |

Blue bars indicate drugs with a positive signal, defined as ≥3 cases and a lower bound of the 95% confidence interval for the ROR > 1. FAERS, U.S. FOOD AND DRUG ADMINISTRATION Adverse Event Reporting System; ROR, reporting odds ratio; CI, confidence interval.

**Supplementary Table 5.** ATC Level 1 and Level 2 classification of drugs reported in association with drug-induced Sweet’s syndrome.

| **Level 1 Category** | **Number of Cases** | **Included Level 2 Categories** | **Number of Cases** | **Drug Name** | **Number of Cases** |
| --- | --- | --- | --- | --- | --- |
| Alimentary tract and metabolism | 152 | Antidiarrheals, intestinal antiinflammatory/antiinfective agents | 56 | Mesalazine | 23 |
|  |  |  |  | Prednisone | 11 |
|  |  |  |  | Sulfasalazine | 8 |
|  |  |  |  | Vancomycin | 6 |
|  |  |  |  | Budesonide | 3 |
|  |  |  |  | Beclometasone | 2 |
|  |  |  |  | Alpha-1-Antitrypsin | 1 |
|  |  |  |  | Bismuth; Metronidazole; Tetracycline | 1 |
|  |  |  |  | Loperamide | 1 |
|  |  | Drugs for acid related disorders | 15 | Lansoprazole | 7 |
|  |  |  |  | Omeprazole | 5 |
|  |  |  |  | Esomeprazole | 2 |
|  |  |  |  | Pantoprazole | 1 |
|  |  | Drugs used in diabetes | 13 | Metformin | 5 |
|  |  |  |  | Dapagliflozin | 2 |
|  |  |  |  | Canagliflozin | 1 |
|  |  |  |  | Empagliflozin; metformin | 1 |
|  |  |  |  | Insulin aspart; insulin degludec | 1 |
|  |  |  |  | Insulin human | 1 |
|  |  |  |  | Insulin lispro | 1 |
|  |  |  |  | Liraglutide | 1 |
|  |  | Stomatological preparations | 68 | Minocycline | 23 |
|  |  |  |  | Dexamethasone | 14 |
|  |  |  |  | Doxycycline | 11 |
|  |  |  |  | Prednisolone | 9 |
|  |  |  |  | Metronidazole | 4 |
|  |  |  |  | Acetylsalicylic acid | 2 |
|  |  |  |  | Triamcinolone | 2 |
|  |  |  |  | Diclofenac; linum usitatissimum;menthol; salicylic acid | 1 |
|  |  |  |  | Epinephrine | 1 |
|  |  |  |  | Hydrocortisone | 1 |
| Antiinfectives for systemic use | 257 | Antibacterials for systemic use | 221 | Ciprofloxacin | 30 |
|  |  |  |  | Levofloxacin | 25 |
|  |  |  |  | Sulfamethoxazole; Trimethoprim | 79 |
|  |  |  |  | Amoxicillin | 18 |
|  |  |  |  | Azithromycin | 14 |
|  |  |  |  | Amoxicillin; Clavulanic Acid | 11 |
|  |  |  |  | Ofloxacin | 9 |
|  |  |  |  | Clarithromycin | 7 |
|  |  |  |  | Ceftriaxone | 4 |
|  |  |  |  | Linezolid | 4 |
|  |  |  |  | Piperacillin; Tazobactam | 4 |
|  |  |  |  | Nitrofurantoin | 3 |
|  |  |  |  | Cefazolin | 2 |
|  |  |  |  | Cefuroxime | 2 |
|  |  |  |  | Cilastatin; Imipenem | 2 |
|  |  |  |  | Daptomycin | 2 |
|  |  |  |  | Cefotaxime | 1 |
|  |  |  |  | Dalfopristin; Quinupristin | 1 |
|  |  |  |  | Fosfomycin | 1 |
|  |  |  |  | Norfloxacin | 1 |
|  |  |  |  | Sulfamethoxazole | 1 |
|  |  | Antimycobacterials | 4 | Rifampicin | 2 |
|  |  |  |  | Isoniazid | 1 |
|  |  |  |  | Isoniazid; Rifampicin | 1 |
|  |  | Antimycotics for systemic use | 4 | Isavuconazole | 1 |
|  |  |  |  | Isavuconazonium | 1 |
|  |  |  |  | Posaconazole | 1 |
|  |  |  |  | Voriconazole | 1 |
|  |  | Antivirals for systemic use | 28 | Lamivudine | 6 |
|  |  |  |  | Stavudine | 4 |
|  |  |  |  | Valaciclovir | 4 |
|  |  |  |  | Lopinavir; Ritonavir | 2 |
|  |  |  |  | Telaprevir | 2 |
|  |  |  |  | Cobicistat; Elvitegravir; Emtricitabine; Tenofovir | 1 |
|  |  |  |  | Dolutegravir | 1 |
|  |  |  |  | Emtricitabine; Tenofovir Disoproxil | 1 |
|  |  |  |  | Enfuvirtide | 1 |
|  |  |  |  | Ledipasvir; Sofosbuvir | 1 |
|  |  |  |  | Oseltamivir | 1 |
|  |  |  |  | Ribavirin | 1 |
|  |  |  |  | Ritonavir | 1 |
|  |  |  |  | Zanamivir | 1 |
|  |  |  |  | Zidovudine | 1 |
| Antineoplastic and immunomodulating agents | 1083 | Antineoplastic agents | 549 | Azacitidine | 69 |
|  |  |  |  | Methotrexate | 32 |
|  |  |  |  | Celecoxib | 28 |
|  |  |  |  | Bortezomib | 27 |
|  |  |  |  | Ibrutinib | 20 |
|  |  |  |  | Cytarabine | 18 |
|  |  |  |  | Gilteritinib | 18 |
|  |  |  |  | Carboplatin | 17 |
|  |  |  |  | Venetoclax | 17 |
|  |  |  |  | Topotecan | 15 |
|  |  |  |  | Decitabine | 14 |
|  |  |  |  | Docetaxel | 13 |
|  |  |  |  | Ipilimumab | 13 |
|  |  |  |  | Gemcitabine | 11 |
|  |  |  |  | Rituximab | 11 |
|  |  |  |  | Capecitabine | 10 |
|  |  |  |  | Dasatinib | 10 |
|  |  |  |  | Imatinib | 10 |
|  |  |  |  | Ixazomib | 9 |
|  |  |  |  | Erlotinib | 8 |
|  |  |  |  | Bendamustine | 7 |
|  |  |  |  | Dabrafenib | 7 |
|  |  |  |  | Enasidenib | 7 |
|  |  |  |  | Mercaptopurine | 7 |
|  |  |  |  | Etoposide | 6 |
|  |  |  |  | Nilotinib | 6 |
|  |  |  |  | Nivolumab | 6 |
|  |  |  |  | Sorafenib | 6 |
|  |  |  |  | Midostaurin | 5 |
|  |  |  |  | Mitoxantrone | 5 |
|  |  |  |  | Trastuzumab | 5 |
|  |  |  |  | Vemurafenib | 5 |
|  |  |  |  | Daunorubicin | 4 |
|  |  |  |  | Doxorubicin | 4 |
|  |  |  |  | Everolimus | 4 |
|  |  |  |  | Hydroxycarbamide | 4 |
|  |  |  |  | Idarubicin | 4 |
|  |  |  |  | Pembrolizumab | 4 |
|  |  |  |  | Atezolizumab | 3 |
|  |  |  |  | Cladribine | 3 |
|  |  |  |  | Cyclophosphamide | 3 |
|  |  |  |  | Cytarabine; Daunorubicin | 3 |
|  |  |  |  | Fludarabine | 3 |
|  |  |  |  | Trametinib | 3 |
|  |  |  |  | Bevacizumab | 2 |
|  |  |  |  | Brentuximab Vedotin | 2 |
|  |  |  |  | Cemiplimab | 2 |
|  |  |  |  | Cetuximab | 2 |
|  |  |  |  | Clofarabine | 2 |
|  |  |  |  | Colchicine | 2 |
|  |  |  |  | Fluorouracil | 2 |
|  |  |  |  | Gefitinib | 2 |
|  |  |  |  | Granulocyte Colony Stimulating Factor | 2 |
|  |  |  |  | Ivosidenib | 2 |
|  |  |  |  | Melphalan | 2 |
|  |  |  |  | Olaparib | 2 |
|  |  |  |  | Paclitaxel | 2 |
|  |  |  |  | Pemetrexed | 2 |
|  |  |  |  | Ponatinib | 2 |
|  |  |  |  | Quizartinib | 2 |
|  |  |  |  | Sunitinib | 2 |
|  |  |  |  | Temsirolimus | 2 |
|  |  |  |  | Vorinostat | 2 |
|  |  |  |  | Belantamab Mafodotin | 1 |
|  |  |  |  | Bexarotene | 1 |
|  |  |  |  | Binimetinib | 1 |
|  |  |  |  | Bosutinib | 1 |
|  |  |  |  | Brigatinib | 1 |
|  |  |  |  | Carfilzomib | 1 |
|  |  |  |  | Cedazuridine; Decitabine | 1 |
|  |  |  |  | Chlorambucil | 1 |
|  |  |  |  | Cisplatin | 1 |
|  |  |  |  | Dacarbazine | 1 |
|  |  |  |  | Durvalumab | 1 |
|  |  |  |  | Encorafenib | 1 |
|  |  |  |  | Gemtuzumab Ozogamicin | 1 |
|  |  |  |  | Granulocyte Macrophage Colony Stimulating Factor | 1 |
|  |  |  |  | Idelalisib | 1 |
|  |  |  |  | Lenvatinib | 1 |
|  |  |  |  | Lorlatinib | 1 |
|  |  |  |  | Niraparib | 1 |
|  |  |  |  | Obinutuzumab | 1 |
|  |  |  |  | Ofatumumab | 1 |
|  |  |  |  | Osimertinib | 1 |
|  |  |  |  | Oxaliplatin | 1 |
|  |  |  |  | Palbociclib | 1 |
|  |  |  |  | Panitumumab | 1 |
|  |  |  |  | Pazopanib | 1 |
|  |  |  |  | Trastuzumab Emtansine | 1 |
|  |  |  |  | Vincristine | 1 |
|  |  | Endocrine therapy | 31 | Letrozole | 18 |
|  |  |  |  | Anastrozole | 8 |
|  |  |  |  | Leuprorelin | 2 |
|  |  |  |  | Abiraterone | 1 |
|  |  |  |  | Enzalutamide | 1 |
|  |  |  |  | Goserelin | 1 |
|  |  | Immunostimulants | 98 | Filgrastim | 36 |
|  |  |  |  | Pegfilgrastim | 35 |
|  |  |  |  | Interferon Beta-1B | 5 |
|  |  |  |  | Peginterferon Alfa-2A | 5 |
|  |  |  |  | Glatiramer | 4 |
|  |  |  |  | Immunoglobulin Human Normal | 3 |
|  |  |  |  | Interferon Beta-1A | 3 |
|  |  |  |  | Aldesleukin | 2 |
|  |  |  |  | Interferon Alfa | 2 |
|  |  |  |  | Plerixafor | 2 |
|  |  |  |  | Interferon Alfa-2B | 1 |
|  |  | Immunosuppressants | 405 | Azathioprine | 84 |
|  |  |  |  | Adalimumab | 76 |
|  |  |  |  | Infliximab | 76 |
|  |  |  |  | Lenalidomide | 41 |
|  |  |  |  | Etanercept | 19 |
|  |  |  |  | Vedolizumab | 11 |
|  |  |  |  | Leflunomide | 10 |
|  |  |  |  | Tocilizumab | 10 |
|  |  |  |  | Mycophenolic acid | 9 |
|  |  |  |  | Abatacept | 5 |
|  |  |  |  | Anakinra | 5 |
|  |  |  |  | Efalizumab | 5 |
|  |  |  |  | Secukinumab | 5 |
|  |  |  |  | Upadacitinib | 5 |
|  |  |  |  | Certolizumab pegol | 4 |
|  |  |  |  | Ciclosporin | 4 |
|  |  |  |  | Golimumab | 4 |
|  |  |  |  | Pomalidomide | 4 |
|  |  |  |  | Ustekinumab | 4 |
|  |  |  |  | Alemtuzumab | 3 |
|  |  |  |  | Belimumab | 3 |
|  |  |  |  | Natalizumab | 3 |
|  |  |  |  | Risankizumab | 3 |
|  |  |  |  | Thalidomide | 3 |
|  |  |  |  | Fingolimod | 2 |
|  |  |  |  | Tofacitinib | 2 |
|  |  |  |  | Anifrolumab | 1 |
|  |  |  |  | Apremilast | 1 |
|  |  |  |  | Basiliximab | 1 |
|  |  |  |  | Eculizumab | 1 |
|  |  |  |  | Sarilumab | 1 |
| Antiparasitic products, insecticides and repellents | 20 | Antiprotozoals | 20 | Hydroxychloroquine | 16 |
|  |  |  |  | Chloroquine | 4 |
| Blood and blood-forming organs | 31 | Antianemic preparations | 5 | Darbepoetin Alfa | 5 |
|  |  | Antihemorrhagics | 1 | Eltrombopag | 1 |
|  |  | Antithrombotic agents | 25 | Epoprostenol | 1 |
|  |  |  |  | Clopidogrel | 14 |
|  |  |  |  | Apixaban | 4 |
|  |  |  |  | Dabigatran | 2 |
|  |  |  |  | Rivaroxaban | 2 |
|  |  |  |  | Enoxaparin | 1 |
|  |  |  |  | Ticagrelor | 1 |
| Cardiovascular system | 152 | Agents acting on the renin-angiotensin system | 14 | Enalapril | 4 |
|  |  |  |  | Ramipril | 4 |
|  |  |  |  | Valsartan | 4 |
|  |  |  |  | Aliskiren | 1 |
|  |  |  |  | Olmesartan | 1 |
|  |  | Antihypertensives | 53 | Hydralazine | 50 |
|  |  |  |  | Minoxidil | 3 |
|  |  | Beta blocking agents | 7 | Bisoprolol | 5 |
|  |  |  |  | Metoprolol | 2 |
|  |  | Calcium channel blockers | 8 | Amlodipine | 6 |
|  |  |  |  | Calcium | 1 |
|  |  |  |  | Nicardipine | 1 |
|  |  | Cardiac therapy | 31 | Ibuprofen | 17 |
|  |  |  |  | Amiodarone | 12 |
|  |  |  |  | Dronedarone | 1 |
|  |  |  |  | Ivabradine | 1 |
|  |  | Diuretics | 27 | Furosemide | 22 |
|  |  |  |  | Hydrochlorothiazide | 4 |
|  |  |  |  | Bendroflumethiazide | 1 |
|  |  | Lipid modifying agents | 10 | Atorvastatin | 3 |
|  |  |  |  | Rosuvastatin | 3 |
|  |  |  |  | Atorvastatin; ezetimibe | 1 |
|  |  |  |  | Ezetimibe; simvastatin | 1 |
|  |  |  |  | Fenofibrate | 1 |
|  |  |  |  | Simvastatin | 1 |
|  |  | Vasoprotectives | 2 | Diltiazem | 2 |
| Dermatologicals | 116 | Anti-acne preparations | 38 | Clindamycin | 19 |
|  |  |  |  | Isotretinoin | 9 |
|  |  |  |  | Tretinoin | 9 |
|  |  |  |  | Dapsone | 1 |
|  |  | Antibiotics and chemotherapeutics for dermatological use | 3 | Aciclovir | 2 |
|  |  |  |  | Amikacin | 1 |
|  |  | Antifungals for dermatological use | 17 | Fluconazole | 7 |
|  |  |  |  | Ketoconazole | 6 |
|  |  |  |  | Terbinafine | 4 |
|  |  | Antipruritics, incl. Antihistamines, anesthetics, etc. | 2 | Diphenhydramine | 1 |
|  |  |  |  | Promethazine | 1 |
|  |  | Antipsoriatics | 3 | Fumaric Acid | 3 |
|  |  | Corticosteroids, dermatological preparations | 6 | Methylprednisolone | 6 |
|  |  | Other dermatological preparations | 47 | Ruxolitinib | 17 |
|  |  |  |  | Diclofenac | 16 |
|  |  |  |  | Dupilumab | 9 |
|  |  |  |  | Tacrolimus | 2 |
|  |  |  |  | Finasteride | 1 |
|  |  |  |  | Mequinol;tretinoin | 1 |
|  |  |  |  | Pimecrolimus | 1 |
| Genito-urinary system and sex hormones | 20 | Other gynecologicals | 7 | Naproxen | 6 |
|  |  |  |  | Intrauterine contraceptive device | 1 |
|  |  | Sex hormones and modulators of the genital system | 8 | Ethinylestradiol; levonorgestrel | 3 |
|  |  |  |  | Levonorgestrel | 3 |
|  |  |  |  | Etonogestrel | 1 |
|  |  |  |  | Ulipristal | 1 |
|  |  | Urologicals | 5 | Tamsulosin | 2 |
|  |  |  |  | Alfuzosin | 1 |
|  |  |  |  | Dutasteride | 1 |
|  |  |  |  | Testosterone | 1 |
| Musculoskeletal system | 30 | Antigout preparations | 9 | Allopurinol | 7 |
|  |  |  |  | Febuxostat | 1 |
|  |  |  |  | Pegloticase | 1 |
|  |  | Antiinflammatory and antirheumatic products | 10 | Diclofenac; Misoprostol | 2 |
|  |  |  |  | Flurbiprofen | 2 |
|  |  |  |  | Ketorolac | 2 |
|  |  |  |  | Fluticasone; Salmeterol | 1 |
|  |  |  |  | Ibuprofen; Pseudoephedrine | 1 |
|  |  |  |  | Rofecoxib | 1 |
|  |  |  |  | Valdecoxib | 1 |
|  |  | Drugs for treatment of bone diseases | 8 | Alendronic Acid | 2 |
|  |  |  |  | Denosumab | 2 |
|  |  |  |  | Risedronic Acid | 2 |
|  |  |  |  | Zoledronic Acid | 2 |
|  |  | Muscle relaxants | 3 | Cyclobenzaprine | 2 |
|  |  |  |  | Tizanidine | 1 |
| Nervous system | 116 | Analgesics | 50 | Gabapentin | 21 |
|  |  |  |  | Paracetamol | 18 |
|  |  |  |  | Codeine; Paracetamol | 6 |
|  |  |  |  | Pregabalin | 2 |
|  |  |  |  | Butalbital; Caffeine; Paracetamol | 1 |
|  |  |  |  | Erenumab | 1 |
|  |  |  |  | Lauromacrogol 400 | 1 |
|  |  | Anesthetics | 3 | Fentanyl | 3 |
|  |  | Antiepileptics | 24 | Lamotrigine | 11 |
|  |  |  |  | Levetiracetam | 6 |
|  |  |  |  | Carbamazepine | 5 |
|  |  |  |  | Gabapentin Enacarbil | 1 |
|  |  |  |  | Topiramate | 1 |
|  |  | Other nervous system drugs | 3 | Varenicline | 2 |
|  |  |  |  | Tetrabenazine | 1 |
|  |  | Psychoanaleptics | 10 | Donepezil | 2 |
|  |  |  |  | Escitalopram | 2 |
|  |  |  |  | Methylphenidate | 2 |
|  |  |  |  | Amitriptyline | 1 |
|  |  |  |  | Bupropion | 1 |
|  |  |  |  | Citalopram | 1 |
|  |  |  |  | Sertraline | 1 |
|  |  | Psycholeptics | 26 | Aripiprazole | 10 |
|  |  |  |  | Clozapine | 9 |
|  |  |  |  | Diazepam | 3 |
|  |  |  |  | Olanzapine | 2 |
|  |  |  |  | Perphenazine | 1 |
|  |  |  |  | Zolpidem | 1 |
| Respiratory system | 9 | Cough and cold preparations | 1 | Codeine | 1 |
|  |  | Drugs for obstructive airway diseases | 8 | Glycopyrronium; Indacaterol | 3 |
|  |  |  |  | Omalizumab | 3 |
|  |  |  |  | Benralizumab | 1 |
|  |  |  |  | Montelukast | 1 |
| Sensory organs | 2 | Ophthalmologicals | 2 | Ranibizumab | 2 |
| Systemic hormonal preparations (excluding sex hormones and insulins) | 21 | Calcium homeostasis | 6 | Calcium Chloride; Glucose; Magnesium Chloride; Sodium Chloride; Sodium Lactate | 3 |
|  |  |  |  | Teriparatide | 2 |
|  |  |  |  | Paricalcitol | 1 |
|  |  | Pituitary and hypothalamic hormones and analogues | 3 | Desmopressin | 2 |
|  |  |  |  | Lanreotide | 1 |
|  |  | Thyroid therapy | 12 | Propylthiouracil | 8 |
|  |  |  |  | Thiamazole | 3 |
|  |  |  |  | Levothyroxine | 1 |
| Various | 9 | All other therapeutic products | 4 | Deferasirox | 4 |
|  |  | Contrast media | 2 | Iopamidol | 1 |
|  |  |  |  | Iopromide | 1 |
|  |  | Therapeutic radiopharmaceuticals | 1 | Ibritumomab tiuxetan | 1 |
|  |  | Various | 2 | Various | 2 |

ATC, Anatomical Therapeutic Chemical.

**Supplementary Table 6.** Sex-based distribution across 14 ATC drug classes associated with drug-induced Sweet’s syndrome.

| **ATC Level 1 Drug Class** | **Male** | **Female** |
| --- | --- | --- |
| Alimentary tract and metabolism | 0.718 | -0.718 |
| Antiinfectives for systemic use | -0.418 | 0.418 |
| Antineoplastic and immunomodulating agents | 0.554 | -0.554 |
| Antiparasitic products, insecticides and repellents | -3.351 | 3.351 |
| Blood and blood-forming organs | -2.058 | 2.058 |
| Cardiovascular system | -1.174 | 1.174 |
| Dermatologicals | 0.199 | -0.199 |
| Genito-urinary system and sex hormones | -0.985 | 0.985 |
| Musculoskeletal system | -1.0168 | 1.017 |
| Nervous system | 3.094 | -3.094 |
| Respiratory system | -0.0759 | 0.0759 |
| Sensory organs | 0.163 | -0.163 |
| Systemic hormonal preparations (excluding sex hormones and insulins) | -1.729 | 1.729 |
| Various | 1.906 | -1.906 |

Pearson’s chi-square test of independence was used to assess the association between sex (male vs. female) and ATC Level 1 drug class (χ² = 34.97, df = 13, *P* = 0.0009). Standardized residuals with an absolute value > 2.0 indicate significant deviations from the expected distribution.

**Supplementary Table 7.** Chi‑square analysis of age‑group distribution across 14 ATC Level 1 drug classes associated with drug-induced Sweet’s syndrome.

| **AgeATC Level 1 Drug Class** | **Age** | | | | | | | | | | | | | | | | | | | |
| --- | --- | --- | --- | --- | --- | --- | --- | --- | --- | --- | --- | --- | --- | --- | --- | --- | --- | --- | --- | --- |
|  | **0-5** | **5-10** | **10-15** | **15-20** | **20-25** | **25-30** | **30-35** | **35-40** | **40-45** | **45-50** | **50-55** | **55-60** | **60-65** | **65-70** | **70-75** | **75-80** | **80-85** | **85-90** | **90-95** |  |
| Alimentary tract and metabolism | 1.619 | -1.221 | 2.553 | -0.610 | 4.450 | -0.776 | -0.843 | 0.481 | -1.003 | 1.336 | 0.913 | -0.657 | -0.238 | -0.556 | -2.158 | -0.174 | -0.301 | 1.124 | -0.526 |  |
| Antiinfectives for systemic use | -1.596 | 1.216 | -1.596 | -1.266 | -1.923 | -3.067 | 2.850 | 1.797 | 3.944 | 0.722 | 3.114 | -0.589 | -0.600 | -0.562 | -2.004 | -2.660 | -2.229 | -2.189 | 2.582 |  |
| Antineoplastic and immunomodulating agents | 0.237 | 0.982 | 0.756 | 1.072 | -2.116 | 1.341 | -2.525 | 0.448 | -2.720 | -1.492 | 0.167 | 1.950 | 2.062 | -0.323 | -2.026 | 3.242 | 2.405 | -0.794 | -0.588 |  |
| Antiparasitic products, insecticides and repellents | -0.374 | -0.387 | -0.374 | -0.444 | -0.567 | -0.719 | 2.629 | 3.274 | 2.687 | -1.193 | -1.307 | 0.507 | -1.131 | -1.360 | -0.373 | -0.860 | -0.523 | -0.513 | -0.167 |  |
| Blood and blood-forming organs | -0.502 | -0.518 | -0.502 | -0.595 | -0.760 | -0.965 | -1.216 | -1.355 | -1.543 | -0.901 | -0.454 | -1.699 | -0.787 | 0.697 | 8.996 | -0.225 | -0.701 | -0.689 | -0.224 |  |
| Cardiovascular system | -1.131 | -1.168 | -1.131 | -1.341 | -1.713 | -1.656 | -2.739 | -2.672 | 1.980 | 3.013 | -0.259 | -0.991 | 0.040 | 2.156 | 2.826 | -1.280 | 0.520 | -0.128 | -0.504 |  |
| Dermatologicals | -1.002 | 1.047 | 1.148 | 4.274 | 7.104 | 2.092 | 2.688 | -1.014 | -1.945 | 0.845 | -2.476 | 0.808 | -0.726 | -0.330 | -1.976 | -1.327 | -1.399 | -1.375 | -0.446 |  |
| Genito-urinary system and sex hormones | -0.360 | -0.372 | -0.360 | -0.427 | -0.546 | -0.693 | -0.873 | 1.249 | -1.108 | 1.748 | -0.362 | -1.221 | -1.090 | 2.172 | -0.293 | 1.735 | -0.503 | -0.495 | -0.161 |  |
| Musculoskeletal system | -0.459 | -0.475 | -0.459 | -0.545 | -0.696 | 0.308 | -1.113 | -1.240 | 0.158 | -0.703 | -0.897 | -0.104 | -0.593 | 0.391 | 0.634 | -1.055 | 0.969 | 9.203 | -0.205 |  |
| Nervous system | -0.973 | -1.006 | -0.973 | -1.154 | -1.475 | 4.016 | 3.364 | -1.328 | 1.665 | -2.351 | -2.351 | -0.783 | -1.368 | 0.197 | 3.555 | -0.737 | -1.360 | 1.906 | -0.434 |  |
| Respiratory system | -0.264 | -0.273 | -0.264 | -0.313 | -0.400 | -0.507 | 1.023 | -0.713 | -0.812 | 0.472 | 2.741 | 0.358 | -0.798 | 0.225 | -0.891 | -0.606 | -0.369 | -0.362 | -0.118 |  |
| Sensory organs | -0.141 | -0.146 | -0.141 | -0.167 | -0.213 | -0.271 | -0.341 | -0.380 | -0.433 | -0.449 | -0.492 | -0.477 | -0.426 | -0.512 | 1.869 | -0.324 | 4.990 | -0.193 | -0.063 |  |
| Systemic hormonal preparations (excluding sex hormones and insulins) | 7.798 | -0.387 | -0.374 | -0.444 | -0.567 | -0.719 | -0.906 | 3.274 | -1.151 | -1.193 | -0.442 | -1.267 | 1.787 | -1.360 | -1.263 | 2.846 | -0.523 | -0.513 | -0.167 |  |
| Various | 2.739 | -0.342 | -0.331 | -0.393 | -0.502 | -0.637 | -0.803 | -0.895 | 0.062 | -1.057 | -0.182 | 0.877 | 0.095 | -1.204 | 1.890 | 0.631 | 1.756 | -0.455 | -0.148 |  |

Pearson’s chi-square test of independence was used to assess the association between age and ATC Level 1 drug class (χ² = 714.1, df = 234, *P* = 0.000). Standardized residuals with an absolute value > 2.0 indicate significant deviations from the expected distribution.

**Supplementary Table 8.** Linear regression trends (2004–2024) in drug-induced Sweet’s syndrome reports for 14 ATC Level 1 drug classes.

| **ATC Level 1 Drug Class** | **Slope** | ***P*** | **R squared** |
| --- | --- | --- | --- |
| Alimentary tract and metabolism | 0.498 | 0.000230 | 0.539 |
| Antiinfectives for systemic use | 0.689 | 0.00782 | 0.348 |
| Antineoplastic and immunomodulating agents | 2.870 | 0.00000198 | 0.704 |
| Antiparasitic products, insecticides and repellents | 0.0484 | 0.795 | 0.0189 |
| Blood and blood-forming organs | 0.113 | 0.674 | 0.0268 |
| Cardiovascular system | 0.688 | 0.007 | 0.325 |
| Dermatologicals | 0.166 | 0.238 | 0.0766 |
| Genito-urinary system and sex hormones | 0.0545 | 0.339 | 0.130 |
| Musculoskeletal system | 0.0335 | 0.607 | 0.0275 |
| Nervous system | 0.380 | 0.0914 | 0.190 |
| Respiratory system | 0.0111 | 0.688 | 0.0445 |
| Sensory organs | 0.000 | 0.000 | 0.000 |
| Systemic hormonal preparations (excluding sex hormones and insulins) | 0.0543 | 0.509 | 0.0758 |
| Various | 0.0316 | 0.438 | 0.103 |

R² indicates the proportion of variance explained by the model. A two-sided *P* < 0.05 was considered statistically significant

**Supplementary Table 9.** Frequently reported drugs by medical doctors without disproportionality signals in FAERS: supplementary assessment using VigiBase, published literature, and product labels.

| **Drug Name** | **Case Number** | **ROR** | **ROR 95% CI** | **Literature Support** | **Clinical Evidence Summary** | **Label Listing (FDA/EMA)** |
| --- | --- | --- | --- | --- | --- | --- |
| Lenalidomide | 51 | 4.24 | 2.89-6.23 | Yes (PMID: 21426467) | ·Chronic lymphocytic leukaemia ·39 days after drug use ·Confirmed DISS ·Without concomitant medication ·Case report | No |
| Etanercept | 19 | 0.40 | 0.24-0.65 | No literature available | None | No |
| Ruxolitinib | 18 | 0.39 | 0.24-0.65 | Yes (PMID: 25707420) | ·Post-essential thrombocythaemia myelofibrosis ·Not specified ·Unclear ·Without concomitant medication ·Case report | No |
| Dexamethasone | 16 | 1.37 | 0.76-2.47 | No literature available | None | No |
| Paracetamol | 13 | 0.27 | 0.15-0.46 | Yes (PMID: 29121137) | ·Post-op facial fracture ·7 days  ·Confirmed DISS ·Concomitant medication：codeine  ·Case report | No |
| Methotrexate | 12 | 0.85 | 0.46-1.54 | No literature available | None | No |
| Tocilizumab | 10 | 0.99 | 0.50-1.97 | Yes (PMID: 31921506) | · Polymyalgia rheumatica ·4 days after drug use ·Confirmed DISS  ·Without concomitant medication ·Case report | No |
| Lamotrigine | 10 | 0.99 | 0.50-1.97 | Yes (PMID: 32995432) | ·Epilepsy ·7 days ·Confirmed DISS  ·Concomitant medication：Clobazam ·Case report | No |
| Dupilumab | 9 | 0.36 | 0.18-0.71 | Yes (PMID: 37487126) | ·Severe eosinophilic asthma ·14 days  ·Confirmed DISS  ·Without concomitant medication ·Case report | No |
| Amlodipine | 6 | 0.42 | 0.18-0.98 | No literature available | None | No |

Note: Clinical evidence summary was list in the order of main disease, time-to-onset, confirmed DISS or comorbid disease, concomitant medication and study type. DISS, drug-induced Sweet’s syndrome; ROR, reporting odds ratio; CI, confidence interval; FDA, U.S. Food and Drug Administration; EMA, European Medicines Agency.

**Supplementary Table 10.** Disproportionality analysis results of candidate drugs in the main analysis.

| **Drug** | **a** | **b** | **c** | **d** | **ROR (95%CI)** |
| --- | --- | --- | --- | --- | --- |
| Sulfamethoxazole/Trimethoprim | 79 | 11891 | 1272 | 15552246 | 81.23(64.67-102.03) |
| Azacitidine | 56 | 10192 | 1295 | 15553945 | 65.99(50.47-86.3) |
| Hydralazine | 50 | 3521 | 1301 | 15560616 | 169.85(127.8-225.72) |
| Adalimumab | 46 | 308349 | 1305 | 15255788 | 1.74(1.3-2.34) |
| Infliximab | 44 | 102018 | 1307 | 15462119 | 5.10(3.78-6.89) |
| Azathioprine | 37 | 4400 | 1314 | 15559737 | 99.58(71.73-138.24) |
| Filgrastim | 35 | 11243 | 1316 | 15552894 | 36.79(26.29-51.49) |
| Pegfilgrastim | 34 | 78723 | 1317 | 15485414 | 5.08(3.61-7.14) |
| Ciprofloxacin | 30 | 37785 | 1321 | 15526352 | 9.33(6.5-13.4) |
| Celecoxib | 27 | 38459 | 1324 | 15525678 | 8.23(5.62-12.05) |
| Levofloxacin | 25 | 36950 | 1326 | 15527187 | 7.92(5.33-11.77) |
| Minocycline | 23 | 5252 | 1328 | 15558885 | 51.31(33.95-77.55) |
| Furosemide | 22 | 23528 | 1329 | 15540609 | 10.93(7.17-16.67) |
| Methotrexate | 22 | 73378 | 1329 | 15490759 | 3.49(2.29-5.33) |
| Gabapentin | 21 | 64345 | 1330 | 15499792 | 3.80(2.47-5.85) |
| Clindamycin | 19 | 14092 | 1332 | 15550045 | 15.74(10-24.76) |
| Amoxicillin | 18 | 23096 | 1333 | 15541041 | 9.09(5.71-14.47) |
| Paracetamol | 18 | 91522 | 1333 | 15472615 | 2.28(1.43-3.63) |
| Ibuprofen | 17 | 78717 | 1334 | 15485420 | 2.51(1.55-4.05) |
| Diclofenac | 16 | 74137 | 1335 | 15490000 | 2.50(1.53-4.10) |
| Ruxolitinib | 16 | 56641 | 1335 | 15507496 | 3.28(2.00-5.37) |
| Azithromycin | 14 | 24224 | 1337 | 15539913 | 6.72(3.97-11.37) |
| Clopidogrel | 14 | 39308 | 1337 | 15524829 | 4.14(2.44-7.00) |
| Lenalidomide | 13 | 55603 | 1338 | 15508534 | 2.71(1.57-4.68) |
| Amiodarone | 12 | 19940 | 1339 | 15544197 | 6.99(3.96-12.34) |
| Amoxicillin;Clavulanic Acid | 11 | 15337 | 1340 | 15548800 | 8.32(4.60-15.07) |
| Lamotrigine | 11 | 52053 | 1340 | 15512084 | 2.45(1.35-4.43) |
| Mesalazine | 11 | 7070 | 1340 | 15557067 | 18.06(9.97-32.71) |
| Decitabine | 10 | 1378 | 1341 | 15562759 | 84.22(45.11-157.24) |
| Hydroxychloroquine | 9 | 10553 | 1342 | 15553584 | 9.88(5.13-19.04) |
| Ofloxacin | 9 | 1722 | 1342 | 15562415 | 60.61(31.41-116.94) |
| Doxycycline | 8 | 15932 | 1343 | 15548205 | 5.81(2.90-11.65) |
| Leflunomide | 8 | 7879 | 1343 | 15556258 | 11.76(5.87-23.57) |
| Vedolizumab | 8 | 31381 | 1343 | 15532756 | 2.95(1.47-5.91) |
| Allopurinol | 7 | 9387 | 1344 | 15554750 | 8.63(4.11-18.14) |
| Clarithromycin | 7 | 16826 | 1344 | 15547311 | 4.81(2.29-10.12) |
| Fluconazole | 7 | 9776 | 1344 | 15554361 | 8.29(3.94-17.42) |
| Lansoprazole | 7 | 27413 | 1344 | 15536724 | 2.95(1.4-6.2) |
| Mercaptopurine | 7 | 1645 | 1344 | 15562492 | 49.27(23.41-103.72) |
| Prednisolone | 7 | 26133 | 1344 | 15538004 | 3.10(1.47-6.51) |
| Prednisone | 7 | 27732 | 1344 | 15536405 | 2.92(1.39-6.13) |
| Codeine;Paracetamol | 6 | 3113 | 1345 | 15561024 | 22.3(9.99-49.76) |
| Dexamethasone | 6 | 24489 | 1345 | 15539648 | 2.83(1.27-6.31) |
| Ketoconazole | 6 | 4647 | 1345 | 15559490 | 14.94(6.69-33.32) |
| Lamivudine | 6 | 10416 | 1345 | 15553721 | 6.66(2.99-14.86) |
| Sulfasalazine | 6 | 3823 | 1345 | 15560314 | 18.16(8.14-40.51) |
| Bisoprolol | 5 | 10973 | 1346 | 15553164 | 5.27(2.19-12.67) |
| Carbamazepine | 5 | 21280 | 1346 | 15542857 | 2.71(1.13-6.53) |
| Cytarabine | 5 | 5682 | 1346 | 15558455 | 10.17(4.23-24.49) |
| Efalizumab | 5 | 2576 | 1346 | 15561561 | 22.44(9.32-54.05) |
| Methylprednisolone | 5 | 22840 | 1346 | 15541297 | 2.53(1.05-6.08) |
| Vancomycin | 5 | 21373 | 1346 | 15542764 | 2.70(1.12-6.50) |
| Anakinra | 4 | 4210 | 1347 | 15559927 | 10.98(4.11-29.3) |
| Bortezomib | 4 | 7935 | 1347 | 15556202 | 5.82(2.18-15.54) |
| Ceftriaxone | 4 | 10289 | 1347 | 15553848 | 4.49(1.68-11.98) |
| Enalapril | 4 | 5014 | 1347 | 15559123 | 9.21(3.45-24.6) |
| Hydrochlorothiazide | 4 | 8123 | 1347 | 15556014 | 5.69(2.13-15.18) |
| Hydroxycarbamide | 4 | 3439 | 1347 | 15560698 | 13.44(5.03-35.87) |
| Linezolid | 4 | 13263 | 1347 | 15550874 | 3.48(1.3-9.29) |
| Metronidazole | 4 | 16615 | 1347 | 15547522 | 2.78(1.04-7.42) |
| Piperacillin;Tazobactam | 4 | 8737 | 1347 | 15555400 | 5.29(1.98-14.11) |
| Propylthiouracil | 4 | 408 | 1347 | 15563729 | 113.28(42.25-303.71) |
| Stavudine | 4 | 1145 | 1347 | 15562992 | 40.36(15.1-107.89) |
| Terbinafine | 4 | 8910 | 1347 | 15555227 | 5.18(1.94-13.84) |
| Valaciclovir | 4 | 13063 | 1347 | 15551074 | 3.54(1.32-9.43) |
| Ethinylestradiol;Levonorgestrel | 3 | 3835 | 1348 | 15560302 | 9.03(2.91-28.05) |
| Glycopyrronium;Indacaterol | 3 | 3112 | 1348 | 15561025 | 11.13(3.58-34.57) |
| Nitrofurantoin | 3 | 4711 | 1348 | 15559426 | 7.35(2.37-22.83) |
| Venetoclax | 3 | 9645 | 1348 | 15554492 | 3.59(1.16-11.14) |

ROR, reporting odds ratio; CI, confidence interval.

**Supplementary Table 11.** Main analysis (malignancy‑/immune‑related indications excluded): LASSO selections at λ_min and λ_1se.

| **Drugs** | **Coefficient (λmin)** | **Coefficient (λ1se)** |
| --- | --- | --- |
| Intercept | -10.551 | -9.909 |
| Sulfamethoxazole; Trimethoprim | 3.381 | 1.641 |
| Azacitidine | 5.412 | 4.742 |
| Hydralazine | - | - |
| Adalimumab | 1.462 | - |
| Infliximab | 2.096 | - |
| Azathioprine | 4.250 | 2.172 |
| Filgrastim | 5.098 | 4.254 |
| Pegfilgrastim | 2.298 | - |
| Ciprofloxacin | -1.554 | - |
| Celecoxib | -1.409 | - |
| Levofloxacin | 3.158 | 1.948 |
| Minocycline | -0.163 | - |
| Furosemide | -1.060 | - |
| Methotrexate | -1.350 | - |
| Gabapentin | -1.606 | - |
| Clindamycin | -0.881 | - |
| Amoxicillin | 3.122 | 1.402 |
| Paracetamol | 2.307 | - |
| Ibuprofen | 0.587 | - |
| Diclofenac | 2.939 | 0.442 |
| Ruxolitinib | 3.798 | 2.426 |
| Azithromycin | 2.625 | - |
| Clopidogrel | -1.360 | - |
| Lenalidomide | 1.430 | - |
| Amiodarone | -0.946 | - |
| Amoxicillin; Clavulanic acid | -0.911 | - |
| Lamotrigine | -1.245 | - |
| Mesalazine | 3.624 | - |
| Decitabine | 6.151 | 5.435 |
| Hydroxychloroquine | 4.552 | 3.334 |
| Ofloxacin | 5.588 | 4.783 |
| Doxycycline | -1.035 | - |
| Leflunomide | -0.131 | - |
| Vedolizumab | 3.397 | 0.988 |
| Allopurinol | -0.730 | - |
| Clarithromycin | -1.063 | - |
| Fluconazole | -0.682 | - |
| Lansoprazole | -0.942 | - |
| Mercaptopurine | - | - |
| Prednisolone | -0.825 | - |
| Prednisone | -0.816 | - |
| Codeine;paracetamol | -0.179 | - |
| Dexamethasone | 2.274 | - |
| Ketoconazole | -0.231 | - |
| Lamivudine | -0.200 | - |
| Sulfasalazine | - | - |
| Bisoprolol | -0.826 | - |
| Carbamazepine | -0.851 | - |
| Cytarabine | 2.621 | - |
| Efalizumab | - | - |
| Methylprednisolone | -1.046 | - |
| Vancomycin | 1.915 | - |
| Anakinra | - | - |
| Bortezomib | 3.427 | - |
| Ceftriaxone | 2.700 | - |
| Enalapril | -0.312 | - |
| Hydrochlorothiazide | -0.678 | - |
| Hydroxycarbamide | 4.744 | 3.620 |
| Linezolid | 3.335 | 1.554 |
| Metronidazole | 2.241 | - |
| Piperacillin; Tazobactam | -0.762 | - |
| Propylthiouracil | - | - |
| Stavudine | - | - |
| Terbinafine | -0.692 | - |
| Valaciclovir | 3.609 | 2.096 |
| Ethinylestradiol; Levonorgestrel | 3.544 | - |
| Glycopyrronium;indacaterol | - | - |
| Nitrofurantoin | -0.616 | - |
| Venetoclax | 3.492 | - |
| Sex | - | - |
| Age1 | 0.001 | - |

LASSO, least absolute shrinkage and selection operator; λmin, optimal regularization parameter minimizing cross-validated error; λ1se, one-standard-error rule; ‘.’, coefficient not selected (shrunken to zero by LASSO).

**Supplementary Table 12.** Main analysis (malignancy‑/immune‑related indications excluded): Multivariable logistic regression results for λ_min‑selected variables.

| **Drugs** | **OR** | **Lower 95% CI** | **Upper 95% CI** | **Adjusted *P*** |
| --- | --- | --- | --- | --- |
| Sulfamethoxazole; Trimethoprim | 29.896 | 7.291 | 80.799 | 0.000 |
| Azacitidine | 223.655 | 129.471 | 371.881 | 0.000 |
| Adalimumab | 4.463 | 1.556 | 10.086 | 0.001 |
| Infliximab | 8.350 | 3.450 | 17.214 | 0.000 |
| Azathioprine | 71.687 | 4.050 | 326.436 | 0.000 |
| Filgrastim | 164.998 | 57.506 | 373.259 | 0.000 |
| Pegfilgrastim | 10.121 | 3.897 | 21.661 | 0.000 |
| Ciprofloxacin | 0.000 | 0.000 | 0.000 | 0.985 |
| Celecoxib | 0.000 | 0.000 | 0.000 | 0.987 |
| Levofloxacin | 23.799 | 10.484 | 46.983 | 0.000 |
| Minocycline | 0.000 | 0.000 | 4477953665.896 | 0.996 |
| Furosemide | 0.000 | 0.000 | 0.000 | 0.991 |
| Methotrexate | 0.000 | 0.000 | 0.000 | 0.987 |
| Gabapentin | 0.000 | 0.000 | 0.000 | 0.984 |
| Clindamycin | 0.000 | 0.000 | 0.000 | 0.992 |
| Amoxicillin | 0.000 | 0.000 | 56.293 | 0.000 |
| Paracetamol | 10.292 | 3.589 | 23.260 | 0.000 |
| Ibuprofen | 1.998 | 0.113 | 9.078 | 0.493 |
| Diclofenac | 19.259 | 4.696 | 52.078 | 0.000 |
| Ruxolitinib | 44.942 | 10.914 | 122.541 | 0.000 |
| Azithromycin | 14.254 | 2.334 | 45.701 | 0.000 |
| Clopidogrel | 0.000 | 0.000 | 0.000 | 0.987 |
| Lenalidomide | 4.430 | 0.250 | 20.261 | 0.141 |
| Amiodarone | 0.000 | 0.000 | 0.000 | 0.992 |
| Amoxicillin;  Clavulanic acid | 0.000 | 0.000 | 0.000 | 0.992 |
| Lamotrigine | 0.000 | 0.000 | 0.000 | 0.988 |
| Mesalazine | 38.473 | 2.175 | 174.684 | 0.000 |
| Decitabine | 469.528 | 162.541 | 1074.386 | 0.000 |
| Hydroxychloroquine | 96.035 | 15.721 | 308.043 | 0.000 |
| Ofloxacin | 269.117 | 81.395 | 657.093 | 0.000 |
| Doxycycline | 0.000 | 0.000 | 0.000 | 0.991 |
| Leflunomide | 0.000 | 0.000 | 26272842353.412 | 0.996 |
| Vedolizumab | 30.627 | 5.012 | 98.319 | 0.000 |
| Allopurinol | 0.000 | 0.000 | 0.000 | 0.993 |
| Clarithromycin | 0.000 | 0.000 | 0.000 | 0.991 |
| Fluconazole | 0.000 | 0.000 | 0.000 | 0.994 |
| Lansoprazole | 0.000 | 0.000 | 0.000 | 0.992 |
| Prednisolone | 0.000 | 0.000 | 0.000 | 0.993 |
| Prednisone | 0.000 | 0.000 | 0.000 | 0.993 |
| Codeine; Paracetamol | 0.000 | 0.000 | 0.000 | 0.996 |
| Dexamethasone | 10.182 | 0.576 | 46.165 | 0.021 |
| Ketoconazole | 0.000 | 0.000 | 854816579.908 | 0.996 |
| Lamivudine | 0.000 | 0.000 | 0.000 | 0.996 |
| Bisoprolol | 0.000 | 0.000 | 0.000 | 0.993 |
| Carbamazepine | 0.000 | 0.000 | 0.000 | 0.992 |
| Cytarabine | 14.401 | 0.813 | 65.659 | 0.008 |
| Methylprednisolone | 0.000 | 0.000 | 0.000 | 0.991 |
| Vancomycin | 7.161 | 0.405 | 32.459 | 0.051 |
| Bortezomib | 31.546 | 1.783 | 143.266 | 0.001 |
| Ceftriaxone | 15.471 | 0.875 | 70.176 | 0.007 |
| Enalapril | 0.000 | 0.000 | 0.000 | 0.996 |
| Hydrochlorothiazide | 0.000 | 0.000 | 0.008 | 0.994 |
| Hydroxycarbamide | 115.792 | 18.923 | 372.926 | 0.000 |
| Linezolid | 28.532 | 6.956 | 77.171 | 0.000 |
| Metronidazole | 9.861 | 0.558 | 44.716 | 0.023 |
| Piperacillin; Tazobactam | 0.000 | 0.000 | 0.000 | 0.993 |
| Terbinafine | 0.000 | 0.000 | 0.000 | 0.993 |
| Valaciclovir | 37.361 | 9.097 | 101.308 | 0.000 |
| Ethinylestradiol; Levonorgestrel | 35.909 | 2.021 | 165.672 | 0.000 |
| Nitrofurantoin | 0.000 | 0.000 | 0.000 | 0.994 |
| Venetoclax | 33.620 | 1.900 | 152.784 | 0.000 |
| Age | 1.001 | 0.994 | 1.008 | 0.711 |

OR, odds ratio; CI, confidence interval; adjusted *P* < 0.05 was considered statistically significant.

**Supplementary Table 13.** Disproportionality analysis results of candidate drugs in the Sensitivity analysis A (excluding malignancy-related indications).

| **Drug** | **a** | **b** | **c** | **d** | **ROR (95%CI)** |
| --- | --- | --- | --- | --- | --- |
| Azathioprine | 84 | 5854 | 1495 | 17079004 | 163.93(131.39-204.52) |
| Sulfamethoxazole;Trimethoprim | 79 | 11925 | 1500 | 17072933 | 75.4(60.09-94.61) |
| Adalimumab | 76 | 614079 | 1503 | 16470779 | 1.36(1.08-1.71) |
| Infliximab | 76 | 175827 | 1503 | 16909031 | 4.86(3.86-6.12) |
| Azacitidine | 56 | 10194 | 1523 | 17074664 | 61.59(47.14-80.47) |
| Hydralazine | 50 | 3524 | 1529 | 17081334 | 158.51(119.37-210.48) |
| Filgrastim | 35 | 11248 | 1544 | 17073610 | 34.41(24.6-48.13) |
| Pegfilgrastim | 34 | 78725 | 1545 | 17006133 | 4.75(3.38-6.68) |
| Methotrexate | 31 | 122021 | 1548 | 16962837 | 2.78(1.95-3.97) |
| Ciprofloxacin | 30 | 37831 | 1549 | 17047027 | 8.73(6.08-12.53) |
| Celecoxib | 28 | 40441 | 1551 | 17044417 | 7.61(5.24-11.06) |
| Levofloxacin | 25 | 36973 | 1554 | 17047885 | 7.42(5.00-11.01) |
| Mesalazine | 23 | 12454 | 1556 | 17072404 | 20.26(13.42-30.6) |
| Minocycline | 23 | 5384 | 1556 | 17079474 | 46.89(31.04-70.84) |
| Furosemide | 22 | 23534 | 1557 | 17061324 | 10.24(6.72-15.61) |
| Gabapentin | 21 | 64547 | 1558 | 17020311 | 3.55(2.31-5.47) |
| Clindamycin | 19 | 14106 | 1560 | 17070752 | 14.74(9.37-23.18) |
| Amoxicillin | 18 | 23104 | 1561 | 17061754 | 8.52(5.35-13.55) |
| Paracetamol | 18 | 91722 | 1561 | 16993136 | 2.14(1.34-3.40) |
| Ibuprofen | 17 | 79310 | 1562 | 17005548 | 2.33(1.45-3.76) |
| Diclofenac | 16 | 75793 | 1563 | 17009065 | 2.3(1.40-3.76) |
| Hydroxychloroquine | 16 | 16081 | 1563 | 17068777 | 10.87(6.64-17.78) |
| Ruxolitinib | 16 | 59229 | 1563 | 17025629 | 2.94(1.80-4.82) |
| Azithromycin | 14 | 24237 | 1565 | 17060621 | 6.3(3.72-10.66) |
| Clopidogrel | 14 | 39316 | 1565 | 17045542 | 3.88(2.29-6.56) |
| Lenalidomide | 13 | 55766 | 1566 | 17029092 | 2.53(1.47-4.38) |
| Amiodarone | 12 | 19942 | 1567 | 17064916 | 6.55(3.71-11.57) |
| Amoxicillin;Clavulanic Acid | 11 | 15344 | 1568 | 17069514 | 7.8(4.31-14.12) |
| Doxycycline | 11 | 16142 | 1568 | 17068716 | 7.42(4.10-13.43) |
| Lamotrigine | 11 | 52066 | 1568 | 17032792 | 2.29(1.27-4.15) |
| Vedolizumab | 11 | 59728 | 1568 | 17025130 | 2.00(1.11-3.62) |
| Decitabine | 10 | 1378 | 1569 | 17083480 | 79.01(42.33-147.47) |
| Leflunomide | 10 | 15520 | 1569 | 17069338 | 7.01(3.76-13.06) |
| Prednisone | 10 | 31721 | 1569 | 17053137 | 3.43(1.84-6.38) |
| Tocilizumab | 10 | 56834 | 1569 | 17028024 | 1.91(1.03-3.56) |
| Ofloxacin | 9 | 1723 | 1570 | 17083135 | 56.84(29.47-109.63) |
| Prednisolone | 9 | 30446 | 1570 | 17054412 | 3.21(1.67-6.18) |
| Propylthiouracil | 8 | 619 | 1571 | 17084239 | 140.55(69.85-282.79) |
| Sulfasalazine | 8 | 7527 | 1571 | 17077331 | 11.55(5.77-23.15) |
| Allopurinol | 7 | 9474 | 1572 | 17075384 | 8.03(3.82-16.87) |
| Clarithromycin | 7 | 16844 | 1572 | 17068014 | 4.51(2.15-9.48) |
| Fluconazole | 7 | 9780 | 1572 | 17075078 | 7.77(3.70-16.34) |
| Lansoprazole | 7 | 27435 | 1572 | 17057423 | 2.77(1.32-5.82) |
| Mercaptopurine | 7 | 1891 | 1572 | 17082967 | 40.23(19.12-84.64) |
| Codeine;Paracetamol | 6 | 3130 | 1573 | 17081728 | 20.82(9.33-46.44) |
| Dexamethasone | 6 | 24651 | 1573 | 17060207 | 2.64(1.18-5.89) |
| Ketoconazole | 6 | 4651 | 1573 | 17080207 | 14.01(6.28-31.24) |
| Lamivudine | 6 | 10416 | 1573 | 17074442 | 6.25(2.80-13.94) |
| Methylprednisolone | 6 | 24708 | 1573 | 17060150 | 2.63(1.18-5.87) |
| Vancomycin | 6 | 21391 | 1573 | 17063467 | 3.04(1.36-6.78) |
| Anakinra | 5 | 7960 | 1574 | 17076898 | 6.81(2.83-16.4) |
| Bisoprolol | 5 | 10980 | 1574 | 17073878 | 4.94(2.05-11.89) |
| Carbamazepine | 5 | 21286 | 1574 | 17063572 | 2.55(1.06-6.13) |
| Cytarabine | 5 | 5682 | 1574 | 17079176 | 9.55(3.97-22.98) |
| Efalizumab | 5 | 2595 | 1574 | 17082263 | 20.91(8.68-50.35) |
| Bortezomib | 4 | 7969 | 1575 | 17076889 | 5.44(2.04-14.52) |
| Ceftriaxone | 4 | 10295 | 1575 | 17074563 | 4.21(1.58-11.24) |
| Chloroquine | 4 | 548 | 1575 | 17084310 | 79.18(29.57-211.98) |
| Enalapril | 4 | 5015 | 1575 | 17079843 | 8.65(3.24-23.08) |
| Hydrochlorothiazide | 4 | 8129 | 1575 | 17076729 | 5.34(2.00-14.24) |
| Hydroxycarbamide | 4 | 3440 | 1575 | 17081418 | 12.61(4.72-33.66) |
| Linezolid | 4 | 13266 | 1575 | 17071592 | 3.27(1.22-8.72) |
| Piperacillin;Tazobactam | 4 | 8747 | 1575 | 17076111 | 4.96(1.86-13.23) |
| Stavudine | 4 | 1145 | 1575 | 17083713 | 37.89(14.18-101.26) |
| Terbinafine | 4 | 8914 | 1575 | 17075944 | 4.87(1.82-12.98) |
| Valaciclovir | 4 | 13065 | 1575 | 17071793 | 3.32(1.24-8.85) |
| Ethinylestradiol;Levonorgestrel | 3 | 3835 | 1576 | 17081023 | 8.48(2.73-26.33) |
| Glycopyrronium;Indacaterol | 3 | 3112 | 1576 | 17081746 | 10.45(3.36-32.45) |
| Mitoxantrone | 3 | 1207 | 1576 | 17083651 | 26.94(8.67-83.75) |
| Nitrofurantoin | 3 | 4713 | 1576 | 17080145 | 6.90(2.22-21.42) |
| Thiamazole | 3 | 1867 | 1576 | 17082991 | 17.42(5.61-54.11) |
| Venetoclax | 3 | 9653 | 1576 | 17075205 | 3.37(1.08-10.45) |

ROR, reporting odds ratio; CI, confidence interval.

**Supplementary Table 14. S**ensitivity Analysis A (malignancy‑related indications excluded): LASSO selections at λ_min and λ_1se.

| **Drugs** | **Coefficient (λmin)** | **Coefficient (λ1se)** |
| --- | --- | --- |
| Intercept | -10.371 | -9.924 |
| Azathioprine | 4.463 | 3.685 |
| Sulfamethoxazole; Trimethoprim | 3.201 | 2.181 |
| Adalimumab | 1.456 | 0.364 |
| Infliximab | 1.969 | 1.040 |
| Azacitidine | 5.296 | 4.790 |
| Hydralazine | - | - |
| Filgrastim | 4.953 | 4.358 |
| Pegfilgrastim | 2.113 | 0.903 |
| Methotrexate | -0.155 | - |
| Ciprofloxacin | -0.100 | - |
| Celecoxib | - | - |
| Levofloxacin | 2.999 | 2.207 |
| Mesalazine | 2.620 | - |
| Minocycline | - | - |
| Furosemide | - | - |
| Gabapentin | -0.149 | - |
| Clindamycin | - | - |
| Amoxicillin | 2.937 | 1.926 |
| Paracetamol | 2.097 | 0.754 |
| Ibuprofen | - | - |
| Diclofenac | 2.712 | 1.440 |
| Hydroxychloroquine | 4.238 | 3.494 |
| Ruxolitinib | 3.636 | 2.761 |
| Azithromycin | 2.388 | 0.196 |
| Clopidogrel | - | - |
| Lenalidomide | 1.029 | - |
| Amiodarone | - | - |
| Amoxicillin; Clavulanic acid | - | - |
| Doxycycline | - | - |
| Lamotrigine | - | - |
| Vedolizumab | 2.485 | 0.506 |
| Decitabine | 6.029 | 5.498 |
| Leflunomide | - | - |
| Prednisone | - | - |
| Tocilizumab | 2.280 | 0.904 |
| Ofloxacin | 5.449 | 4.874 |
| Prednisolone | - | - |
| Propylthiouracil | - | - |
| Sulfasalazine | 3.031 | 0.694 |
| Allopurinol | - | - |
| Clarithromycin | - | - |
| Fluconazole | - | - |
| Lansoprazole | - | - |
| Mercaptopurine | - | - |
| Codeine; Paracetamol | - | - |
| Dexamethasone | 1.963 | - |
| Ketoconazole | - | - |
| Lamivudine | - | - |
| Methylprednisolone | - | - |
| Vancomycin | 1.568 | - |
| Anakinra | - | - |
| Bisoprolol | - | - |
| Carbamazepine | - | - |
| Cytarabine | 2.329 | - |
| Efalizumab | - | - |
| Bortezomib | 3.211 | 1.288 |
| Ceftriaxone | 2.428 | - |
| Chloroquine | - | - |
| Enalapril | - | - |
| Hydrochlorothiazide | - | - |
| Hydroxycarbamide | 4.594 | 3.842 |
| Linezolid | 3.158 | 2.121 |
| Piperacillin; Tazobactam | - | - |
| Stavudine | - | - |
| Terbinafine | - | - |
| Valaciclovir | 3.444 | 2.511 |
| Ethinylestradiol; Levonorgestrel | 3.301 | 1.524 |
| Glycopyrronium; Indacaterol | - | - |
| Mitoxantrone | - | - |
| Nitrofurantoin | - | - |
| Thiamazole | - | - |
| Venetoclax | 3.281 | 1.474 |
| Sex | - | - |
| Age | - | - |

LASSO, least absolute shrinkage and selection operator; λmin, optimal regularization parameter minimizing cross-validated error; λ1se, one-standard-error rule; ‘.’, coefficient not selected (shrunken to zero by LASSO).

**Supplementary Table 15.** Sensitivity Analysis A (malignancy‑related indications excluded): Multivariable logistic regression results for λ_min‑selected variables.

| **Drugs** | **OR** | **Lower 95% CI** | **Upper 95% CI** | **Adjusted *P*** |
| --- | --- | --- | --- | --- |
| Intercept | 0.000 | 0.000 | 0.000 | 0.000 |
| Azathioprine | 99.475 | 16.314 | 317.602 | 0.000 |
| Sulfamethoxazole; Trimethoprim | 28.841 | 7.052 | 77.520 | 0.000 |
| Adalimumab | 5.071 | 2.736 | 8.737 | 0.000 |
| Infliximab | 8.347 | 4.405 | 14.599 | 0.000 |
| Azacitidine | 220.810 | 130.497 | 357.193 | 0.000 |
| Hydralazine | 158.789 | 55.539 | 356.882 | 0.000 |
| Filgrastim | 9.862 | 3.818 | 20.910 | 0.000 |
| Pegfilgrastim | 0.000 | 0.000 | 0.000 | 0.975 |
| Methotrexate | 0.000 | 0.000 | 0.000 | 0.976 |
| Ciprofloxacin | 23.041 | 10.202 | 45.114 | 0.000 |
| Mesalazine | 17.677 | 1.001 | 79.851 | 0.004 |
| Gabapentin | 0.000 | 0.000 | 0.000 | 0.975 |
| Amoxicillin | 22.130 | 6.730 | 53.483 | 0.000 |
| Paracetamol | 9.799 | 3.432 | 21.973 | 0.000 |
| Diclofenac | 18.039 | 4.411 | 48.474 | 0.000 |
| Hydroxychloroquine | 79.107 | 19.332 | 212.856 | 0.000 |
| Ruxolitinib | 43.982 | 10.752 | 118.253 | 0.000 |
| Azithromycin | 13.571 | 2.228 | 43.223 | 0.000 |
| Lenalidomide | 4.373 | 0.248 | 19.741 | 0.143 |
| Vedolizumab | 14.863 | 2.440 | 47.340 | 0.000 |
| Decitabine | 461.284 | 160.904 | 1041.519 | 0.000 |
| Tocilizumab | 11.791 | 3.586 | 28.490 | 0.000 |
| Ofloxacin | 259.965 | 78.874 | 630.912 | 0.000 |
| Sulfasalazine | 25.881 | 1.465 | 116.956 | 0.001 |
| Dexamethasone | 9.753 | 0.552 | 44.041 | 0.024 |
| Vancomycin | 6.897 | 0.391 | 31.137 | 0.055 |
| Cytarabine | 13.558 | 0.768 | 61.231 | 0.010 |
| Bortezomib | 30.644 | 1.735 | 138.514 | 0.001 |
| Ceftriaxone | 14.835 | 0.840 | 67.002 | 0.007 |
| Hydroxycarbamide | 113.156 | 18.555 | 361.427 | 0.000 |
| Linezolid | 27.684 | 6.769 | 74.408 | 0.000 |
| Valaciclovir | 36.486 | 8.920 | 98.083 | 0.000 |
| Ethinylestradiol; Levonorgestrel | 33.364 | 1.888 | 150.825 | 0.001 |
| Venetoclax | 32.739 | 1.853 | 147.996 | 0.001 |

OR, odds ratio; CI, confidence interval; adjusted *P* < 0.05 was considered statistically significant.

**Supplementary Table 16.** Disproportionality analysis results of candidate drugs in the Sensitivity analysis B (excluding immune-related indications).

| **Drug** | **a** | **b** | **c** | **d** | **ROR (95%CI)** |
| --- | --- | --- | --- | --- | --- |
| Sulfamethoxazole; Trimethoprim | 79 | 11905 | 1711 | 17079348 | 66.24(52.83-83.06) |
| Azacitidine | 69 | 16277 | 1721 | 17074976 | 42.06(33.05-53.53) |
| Hydralazine | 50 | 3524 | 1740 | 17087729 | 139.34(104.99-184.93) |
| Adalimumab | 46 | 308369 | 1744 | 16782884 | 1.44(1.07-1.92) |
| Infliximab | 44 | 102082 | 1746 | 16989171 | 4.19(3.11-5.66) |
| Azathioprine | 37 | 4404 | 1753 | 17086849 | 81.89(59.05-113.56) |
| Filgrastim | 36 | 12181 | 1754 | 17079072 | 28.78(20.68-40.05) |
| Pegfilgrastim | 35 | 81311 | 1755 | 17009942 | 4.17(2.99-5.83) |
| Ciprofloxacin | 30 | 37805 | 1760 | 17053448 | 7.69(5.36-11.03) |
| Bortezomib | 27 | 32272 | 1763 | 17058981 | 8.10(5.53-11.84) |
| Celecoxib | 27 | 38624 | 1763 | 17052629 | 6.76(4.62-9.89) |
| Levofloxacin | 25 | 36968 | 1765 | 17054285 | 6.53(4.40-9.70) |
| Methotrexate | 23 | 81086 | 1767 | 17010167 | 2.73(1.81-4.12) |
| Minocycline | 23 | 5256 | 1767 | 17085997 | 42.31(28.02-63.9) |
| Furosemide | 22 | 23545 | 1768 | 17067708 | 9.02(5.92-13.74) |
| Gabapentin | 21 | 64374 | 1769 | 17026879 | 3.14(2.04-4.83) |
| Clindamycin | 19 | 14100 | 1771 | 17077153 | 12.99(8.27-20.43) |
| Amoxicillin | 18 | 23106 | 1772 | 17068147 | 7.50(4.72-11.94) |
| Cytarabine | 18 | 11936 | 1772 | 17079317 | 14.54(9.13-23.13) |
| Gilteritinib | 18 | 2625 | 1772 | 17088628 | 66.13(41.5-105.37) |
| Ibrutinib | 18 | 65857 | 1772 | 17025396 | 2.63(1.65-4.18) |
| Letrozole | 18 | 20053 | 1772 | 17071200 | 8.65(5.43-13.76) |
| Paracetamol | 18 | 91541 | 1772 | 16999712 | 1.89(1.19-3.00) |
| Carboplatin | 17 | 43559 | 1773 | 17047694 | 3.75(2.33-6.05) |
| Ibuprofen | 17 | 78722 | 1773 | 17012531 | 2.07(1.29-3.34) |
| Ruxolitinib | 17 | 58120 | 1773 | 17033133 | 2.81(1.74-4.53) |
| Venetoclax | 17 | 39916 | 1773 | 17051337 | 4.10(2.54-6.60) |
| Diclofenac | 16 | 74164 | 1774 | 17017089 | 2.07(1.26-3.39) |
| Topotecan | 15 | 3449 | 1775 | 17087804 | 41.87(25.16-69.67) |
| Azithromycin | 14 | 24235 | 1776 | 17067018 | 5.55(3.28-9.39) |
| Clopidogrel | 14 | 39316 | 1776 | 17051937 | 3.42(2.02-5.79) |
| Decitabine | 14 | 2577 | 1776 | 17088676 | 52.27(30.85-88.57) |
| Dexamethasone | 14 | 41806 | 1776 | 17049447 | 3.21(1.9-5.44) |
| Docetaxel | 13 | 51043 | 1777 | 17040210 | 2.44(1.42-4.21) |
| Ipilimumab | 13 | 17402 | 1777 | 17073851 | 7.18(4.16-12.39) |
| Amiodarone | 12 | 19940 | 1778 | 17071313 | 5.78(3.27-10.2) |
| Amoxicillin;Clavulanic Acid | 11 | 15340 | 1779 | 17075913 | 6.88(3.80-12.45) |
| Gemcitabine | 11 | 26301 | 1779 | 17064952 | 4.01(2.22-7.26) |
| Lamotrigine | 11 | 52057 | 1779 | 17039196 | 2.02(1.12-3.66) |
| Mesalazine | 11 | 7072 | 1779 | 17084181 | 14.94(8.25-27.03) |
| Dasatinib | 10 | 29178 | 1780 | 17062075 | 3.29(1.76-6.12) |
| Hydroxychloroquine | 9 | 10597 | 1781 | 17080656 | 8.15(4.23-15.68) |
| Ixazomib | 9 | 16055 | 1781 | 17075198 | 5.37(2.79-10.35) |
| Ofloxacin | 9 | 1723 | 1781 | 17089530 | 50.12(25.99-96.65) |
| Tretinoin | 9 | 2937 | 1781 | 17088316 | 29.4(15.26-56.66) |
| Anastrozole | 8 | 14510 | 1782 | 17076743 | 5.28(2.64-10.58) |
| Doxycycline | 8 | 15950 | 1782 | 17075303 | 4.81(2.40-9.63) |
| Leflunomide | 8 | 7883 | 1782 | 17083370 | 9.73(4.86-19.49) |
| Prednisone | 8 | 31267 | 1782 | 17059986 | 2.45(1.22-4.91) |
| Vedolizumab | 8 | 31388 | 1782 | 17059865 | 2.44(1.22-4.89) |
| Allopurinol | 7 | 9498 | 1783 | 17081755 | 7.06(3.36-14.84) |
| Bendamustine | 7 | 9475 | 1783 | 17081778 | 7.08(3.37-14.87) |
| Clarithromycin | 7 | 16866 | 1783 | 17074387 | 3.97(1.89-8.35) |
| Dabrafenib | 7 | 12904 | 1783 | 17078349 | 5.20(2.47-10.92) |
| Enasidenib | 7 | 3353 | 1783 | 17087900 | 20.01(9.52-42.06) |
| Fluconazole | 7 | 9797 | 1783 | 17081456 | 6.85(3.26-14.38) |
| Lansoprazole | 7 | 27432 | 1783 | 17063821 | 2.44(1.16-5.13) |
| Mercaptopurine | 7 | 2300 | 1783 | 17088953 | 29.17(13.87-61.35) |
| Prednisolone | 7 | 29151 | 1783 | 17062102 | 2.30(1.09-4.83) |
| Codeine;Paracetamol | 6 | 3115 | 1784 | 17088138 | 18.45(8.27-41.15) |
| Etoposide | 6 | 11857 | 1784 | 17079396 | 4.84(2.17-10.8) |
| Ketoconazole | 6 | 4740 | 1784 | 17086513 | 12.12(5.44-27.04) |
| Lamivudine | 6 | 10433 | 1784 | 17080820 | 5.51(2.47-12.28) |
| Sorafenib | 6 | 18791 | 1784 | 17072462 | 3.06(1.37-6.81) |
| Sulfasalazine | 6 | 3824 | 1784 | 17087429 | 15.03(6.74-33.52) |
| Bisoprolol | 5 | 10975 | 1785 | 17080278 | 4.36(1.81-10.49) |
| Efalizumab | 5 | 2576 | 1785 | 17088677 | 18.58(7.72-44.74) |
| Midostaurin | 5 | 2278 | 1785 | 17088975 | 21.01(8.73-50.6) |
| Vemurafenib | 5 | 9474 | 1785 | 17081779 | 5.05(2.10-12.15) |
| Anakinra | 4 | 4284 | 1786 | 17086969 | 8.93(3.35-23.84) |
| Ceftriaxone | 4 | 10297 | 1786 | 17080956 | 3.72(1.39-9.91) |
| Daunorubicin | 4 | 1146 | 1786 | 17090107 | 33.40(12.50-89.24) |
| Enalapril | 4 | 5016 | 1786 | 17086237 | 7.63(2.86-20.36) |
| Hydrochlorothiazide | 4 | 8129 | 1786 | 17083124 | 4.71(1.76-12.56) |
| Hydroxycarbamide | 4 | 3848 | 1786 | 17087405 | 9.95(3.73-26.54) |
| Idarubicin | 4 | 1142 | 1786 | 17090111 | 33.52(12.54-89.55) |
| Linezolid | 4 | 13271 | 1786 | 17077982 | 2.88(1.08-7.69) |
| Piperacillin;Tazobactam | 4 | 8747 | 1786 | 17082506 | 4.37(1.64-11.67) |
| Propylthiouracil | 4 | 408 | 1786 | 17090845 | 93.82(35.00-251.45) |
| Stavudine | 4 | 1145 | 1786 | 17090108 | 33.43(12.51-89.32) |
| Terbinafine | 4 | 8910 | 1786 | 17082343 | 4.29(1.61-11.46) |
| Valaciclovir | 4 | 13145 | 1786 | 17078108 | 2.91(1.09-7.76) |
| Cladribine | 3 | 5279 | 1787 | 17085974 | 5.43(1.75-16.87) |
| Cytarabine;Daunorubicin | 3 | 1535 | 1787 | 17089718 | 18.69(6.02-58.07) |
| Ethinylestradiol;Levonorgestrel | 3 | 3835 | 1787 | 17087418 | 7.48(2.41-23.23) |
| Fludarabine | 3 | 7971 | 1787 | 17083282 | 3.6(1.16-11.17) |
| Glycopyrronium;Indacaterol | 3 | 3123 | 1787 | 17088130 | 9.19(2.96-28.52) |
| Mitoxantrone | 3 | 1682 | 1787 | 17089571 | 17.06(5.49-52.99) |
| Nitrofurantoin | 3 | 4713 | 1787 | 17086540 | 6.09(1.96-18.9) |

ROR, reporting odds ratio; CI, confidence interval.

**Supplementary Table 17.** Sensitivity Analysis B (immune‑related indications excluded): LASSO selections at λ_min and λ_1se.

| **Drugs** | **Coefficient (λmin)** | **Coefficient (λ1se)** |
| --- | --- | --- |
| Intercept | -10.082 | -9.890 |
| Sulfamethoxazole; Trimethoprim | 2.964 | 2.412 |
| Azacitidine | 4.790 | 4.488 |
| Hydralazine | - | - |
| Adalimumab | 0.967 | - |
| Infliximab | 1.634 | 0.996 |
| Azathioprine | 3.808 | 3.214 |
| Filgrastim | 4.596 | 4.256 |
| Pegfilgrastim | 1.850 | 1.199 |
| Ciprofloxacin | -0.279 | - |
| Bortezomib | 3.122 | 2.692 |
| Celecoxib | -0.118 | - |
| Levofloxacin | 2.767 | 2.324 |
| Methotrexate | -0.182 | - |
| Minocycline | - | - |
| Furosemide | - | - |
| Gabapentin | -0.322 | - |
| Clindamycin | - | - |
| Amoxicillin | 2.694 | 2.153 |
| Cytarabine | 2.248 | 1.385 |
| Gilteritinib | 5.218 | 4.876 |
| Ibrutinib | 2.211 | 1.448 |
| Letrozole | - | - |
| Paracetamol | 1.856 | 1.176 |
| Carboplatin | -0.282 | - |
| Ibuprofen | - | - |
| Ruxolitinib | 3.388 | 2.884 |
| Venetoclax | 3.155 | 2.710 |
| Diclofenac | 2.516 | 1.860 |
| Topotecan | - | - |
| Azithromycin | 2.140 | 1.214 |
| Clopidogrel | -0.048 | - |
| Decitabine | 5.415 | 5.098 |
| Dexamethasone | 0.943 | - |
| Docetaxel | -0.416 | - |
| Ipilimumab | 3.290 | 2.846 |
| Amiodarone | - | - |
| Amoxicillin; Clavulanic acid | - | - |
| Gemcitabine | - | - |
| Lamotrigine | -0.005 | - |
| Mesalazine | 3.166 | 2.380 |
| Dasatinib | 3.374 | 2.883 |
| Hydroxychloroquine | 4.135 | 3.693 |
| Ixazomib | 2.848 | 2.157 |
| Ofloxacin | 5.217 | 4.893 |
| Tretinoin | 4.708 | 4.321 |
| Anastrozole | - | - |
| Doxycycline | - | - |
| Leflunomide | - | - |
| Prednisone | - | - |
| Vedolizumab | 2.946 | 2.314 |
| Allopurinol | - | - |
| Bendamustine | 3.321 | 2.812 |
| Clarithromycin | - | - |
| Dabrafenib | 3.024 | 2.395 |
| Enasidenib | - | - |
| Fluconazole | - | - |
| Lansoprazole | - | - |
| Mercaptopurine | - | - |
| Prednisolone | - | - |
| Codeine; Paracetamol | - | - |
| Etoposide | 2.964 | 2.341 |
| Ketoconazole | - | - |
| Lamivudine | - | - |
| Sorafenib | 1.352 | - |
| Sulfasalazine | - | - |
| Bisoprolol | - | - |
| Efalizumab | - | - |
| Midostaurin | 4.008 | 3.442 |
| Vemurafenib | 3.230 | 2.644 |
| Anakinra | - | - |
| Ceftriaxone | 2.180 | 0.700 |
| Daunorubicin | 4.714 | 4.342 |
| Enalapril | - | - |
| Hydrochlorothiazide | - | - |
| Hydroxycarbamide | 4.264 | 3.823 |
| Idarubicin | - | - |
| Linezolid | 2.926 | 2.361 |
| Piperacillin; Tazobactam | - | - |
| Propylthiouracil | - | - |
| Stavudine | - | - |
| Terbinafine | - | - |
| Valaciclovir | 3.210 | 2.689 |
| Cladribine | - | - |
| Cytarabine; Daunorubicin | - | - |
| Ethinylestradiol; Levonorgestrel | 3.036 | 2.229 |
| Fludarabine | - | - |
| Glycopyrronium; Indacaterol | - | - |
| Mitoxantrone | - | - |
| Nitrofurantoin | - | - |
| Sex | - | - |
| Age | -0.001 | - |

LASSO, least absolute shrinkage and selection operator; λmin, optimal regularization parameter minimizing cross-validated error; λ1se, one-standard-error rule; ‘.’, coefficient not selected (shrunken to zero by LASSO).

**Supplementary Table 18.** Sensitivity Analysis B (immune‑related indications excluded): Multivariable logistic regression results for λ_min‑selected variables.

| **Drugs** | **OR** | **Lower 95% CI** | **Upper 95% CI** | **Adjusted *P*** |
| --- | --- | --- | --- | --- |
| Intercept | 0.000 | 0.000 | 0.000 | 0.000 |
| Sulfamethoxazole;  Trimethoprim | 22.201 | 5.452 | 59.116 | 0.000 |
| Azacitidine | 132.412 | 82.311 | 205.734 | 0.000 |
| Adalimumab | 3.233 | 1.138 | 7.178 | 0.011 |
| Infliximab | 5.862 | 2.454 | 11.832 | 0.000 |
| Azathioprine | 51.481 | 2.917 | 231.902 | 0.000 |
| Filgrastim | 107.962 | 37.995 | 239.931 | 0.000 |
| Pegfilgrastim | 7.486 | 2.915 | 15.712 | 0.000 |
| Ciprofloxacin | 0.000 | 0.000 | 0.000 | 0.984 |
| Bortezomib | 25.596 | 10.740 | 51.465 | 0.000 |
| Celecoxib | 0.000 | 0.000 | 0.000 | 0.986 |
| Levofloxacin | 17.875 | 7.977 | 34.552 | 0.000 |
| Methotrexate | 0.000 | 0.000 | 0.000 | 0.986 |
| Gabapentin | 0.000 | 0.000 | 0.000 | 0.983 |
| Amoxicillin | 16.778 | 5.125 | 40.192 | 0.000 |
| Cytarabine | 11.192 | 1.839 | 35.574 | 0.001 |
| Gilteritinib | 202.968 | 49.700 | 543.672 | 0.000 |
| Ibrutinib | 11.076 | 2.711 | 29.695 | 0.000 |
| Paracetamol | 7.431 | 2.615 | 16.515 | 0.000 |
| Carboplatin | 0.000 | 0.000 | 0.000 | 0.984 |
| Ruxolitinib | 34.165 | 8.365 | 91.534 | 0.000 |
| Venetoclax | 26.655 | 10.353 | 56.159 | 0.000 |
| Diclofenac | 14.521 | 3.566 | 38.672 | 0.000 |
| Azithromycin | 10.223 | 1.682 | 32.363 | 0.001 |
| Clopidogrel | 0.000 | 0.000 | 0.000 | 0.987 |
| Decitabine | 247.452 | 95.883 | 523.423 | 0.000 |
| Dexamethasone | 3.890 | 0.221 | 17.447 | 0.177 |
| Docetaxel | 0.000 | 0.000 | 0.000 | 0.982 |
| Ipilimumab | 30.346 | 10.678 | 67.461 | 0.000 |
| Lamotrigine | 0.000 | 0.000 | 0.000 | 0.988 |
| Mesalazine | 28.178 | 1.598 | 126.585 | 0.001 |
| Dasatinib | 33.189 | 8.148 | 88.433 | 0.000 |
| Hydroxychloroquine | 69.880 | 11.499 | 221.246 | 0.000 |
| Ixazomib | 20.670 | 3.396 | 65.686 | 0.000 |
| Ofloxacin | 200.520 | 61.158 | 481.736 | 0.000 |
| Tretinoin | 121.906 | 20.041 | 386.858 | 0.000 |
| Vedolizumab | 21.873 | 3.599 | 69.278 | 0.000 |
| Bendamustine | 31.932 | 7.825 | 85.405 | 0.000 |
| Dabrafenib | 24.030 | 3.957 | 75.966 | 0.000 |
| Etoposide | 22.170 | 3.645 | 70.327 | 0.000 |
| Sorafenib | 5.556 | 0.315 | 24.972 | 0.088 |
| Midostaurin | 63.436 | 3.595 | 285.524 | 0.000 |
| Vemurafenib | 29.311 | 4.825 | 92.700 | 0.000 |
| Ceftriaxone | 11.278 | 0.640 | 50.625 | 0.016 |
| Daunorubicin | 120.348 | 19.734 | 384.239 | 0.000 |
| Hydroxycarbamide | 80.364 | 13.212 | 254.989 | 0.000 |
| Linezolid | 21.515 | 5.283 | 57.306 | 0.000 |
| Valaciclovir | 28.512 | 6.995 | 76.073 | 0.000 |
| Ethinylestradiol; Levonorgestrel | 24.167 | 1.366 | 109.794 | 0.002 |
| AGE | 0.998 | 0.991 | 1.004 | 0.462 |

OR, odds ratio; CI, confidence interval; adjusted *P* < 0.05 was considered statistically significant.

**Supplementary Table 19.** VigiBase-based disproportionality analysis of drugs identified in the main analysis.

| **Drug** | **a** | **b** | **c** | **d** | **ROR** | **Lower 95%CI** | **Upper 95%CI** |
| --- | --- | --- | --- | --- | --- | --- | --- |
| Levofloxacin | 5 | 479 | 453857 | 4410457 | 0.10 | 0.25 | 0.04 |
| Mesalazine | 22 | 104 | 65187 | 652949 | 2.12 | 1.34 | 3.36 |
| Amoxicillin | 16 | 468 | 351272 | 4513042 | 0.44 | 0.27 | 0.72 |
| Azithromycin | 9 | 475 | 200572 | 4663742 | 0.44 | 0.23 | 0.85 |
| Linezolid | 2 | 37 | 66288 | 1041926 | 0.85 | 0.21 | 3.53 |
| Ofloxacin | 6 | 69 | 55135 | 657230 | 1.04 | 0.45 | 2.39 |
| Sulfamethoxazole; Trimethoprim | 2 | 482 | 4145 | 4860169 | 4.87 | 1.21 | 19.52 |
| Valaciclovir | 4 | 35 | 42877 | 1065337 | 2.84 | 1.01 | 7.99 |
| Adalimumab | 61 | 423 | 2073437 | 2790877 | 0.19 | 0.15 | 0.25 |
| Azacitidine | 105 | 379 | 46698 | 4817616 | 28.58 | 23.02 | 35.49 |
| Azathioprine | 126 | 358 | 93139 | 4771175 | 18.03 | 14.72 | 22.09 |
| Bortezomib | 44 | 440 | 116586 | 4747728 | 4.07 | 2.99 | 5.55 |
| Decitabine | 25 | 101 | 14789 | 703347 | 11.77 | 7.60 | 18.24 |
| Filgrastim | 50 | 76 | 56635 | 661501 | 7.68 | 5.38 | 10.98 |
| Hydroxycarbamide | 7 | 32 | 29756 | 1078458 | 7.93 | 3.50 | 17.96 |
| Infliximab | 43 | 441 | 517383 | 4346931 | 0.82 | 0.60 | 1.12 |
| Pegfilgrastim | 50 | 434 | 147108 | 4717206 | 3.69 | 2.76 | 4.95 |
| Vedolizumab | 4 | 71 | 117243 | 595122 | 0.29 | 0.10 | 0.78 |
| Venetoclax | 21 | 18 | 110755 | 997459 | 10.51 | 5.60 | 19.72 |
| Hydroxychloroquine | 12 | 63 | 110345 | 602020 | 1.04 | 0.56 | 1.93 |
| Diclofenac | 11 | 115 | 368098 | 350038 | 0.09 | 0.05 | 0.17 |
| Ruxolitinib | 18 | 108 | 213427 | 504709 | 0.39 | 0.24 | 0.65 |
| Ethinylestradiol; Levonorgestrel | 1 | 38 | 657016 | 451198 | 0.02 | 0.00 | 0.13 |
| Paracetamol | 13 | 471 | 456063 | 4408251 | 0.27 | 0.15 | 0.46 |

Blue bars indicate drugs with a positive signal, defined as ≥3 cases and a lower bound of the 95% confidence interval for the ROR > 1. ROR, reporting odds ratio; CI, confidence interval.

**Supplementary Table 20.** Product‑label documentation of Sweet’s syndrome for drugs identified in the main analysis (FDA/EMA sources).

| **Drug Name** | **Listed as Sweet’s Syndrome in Product Label** | **Label Term** | **Data Source** |
| --- | --- | --- | --- |
| Sulfamethoxazole; Trimethoprim | No |  | FDA, EMA |
| Azacitidine | Yes | Sweet’s Syndrome | FDA |
| Adalimumab | No |  | FDA, EMA |
| Infliximab | Yes | Sweet’s Syndrome | FDA |
| Azathioprine | Yes | Sweet’s Syndrome | FDA |
| Filgrastim | No |  | FDA, EMA |
| Pegfilgrastim | Yes | Sweet’s Syndrome | FDA |
| Levofloxacin | No |  | FDA, EMA |
| Amoxicillin | No |  | FDA, EMA |
| Paracetamol | No |  | FDA, EMA |
| Diclofenac | No |  | FDA, EMA |
| Ruxolitinib | No |  | FDA, EMA |
| Azithromycin | Yes | Sweet’s Syndrome | FDA |
| Mesalazine | Yes | Sweet’s Syndrome | FDA |
| Decitabine | Yes | Sweet’s Syndrome | FDA |
| Hydroxychloroquine | Yes | Sweet’s Syndrome | FDA |
| Ofloxacin | No |  | FDA, EMA |
| Vedolizumab | No |  | FDA, EMA |
| Bortezomib | Yes | Sweet’s Syndrome | FDA |
| Hydroxycarbamide | No |  | EMA |
| Linezolid | No |  | FDA |
| Valaciclovir | No |  | EMA |
| Ethinylestradiol; Levonorgestrel | No |  | FDA, EMA |
| Venetoclax | No |  | FDA, EMA |

FDA, U.S. Food and Drug Administration; EMA, European Medicines Agency.
